# Supplementary figures and images for: Diversity in sea buckthorn (Hippophae rhamnoides L.) accessions with different origins based on morphological characteristics, oil traits, and microsatellite markers
Source: PLoS One. 2020 Mar 13;15(3):e0230356. doi: 10.1371/journal.pone.0230356 (PMC7069629; doi:10.1371/journal.pone.0230356)

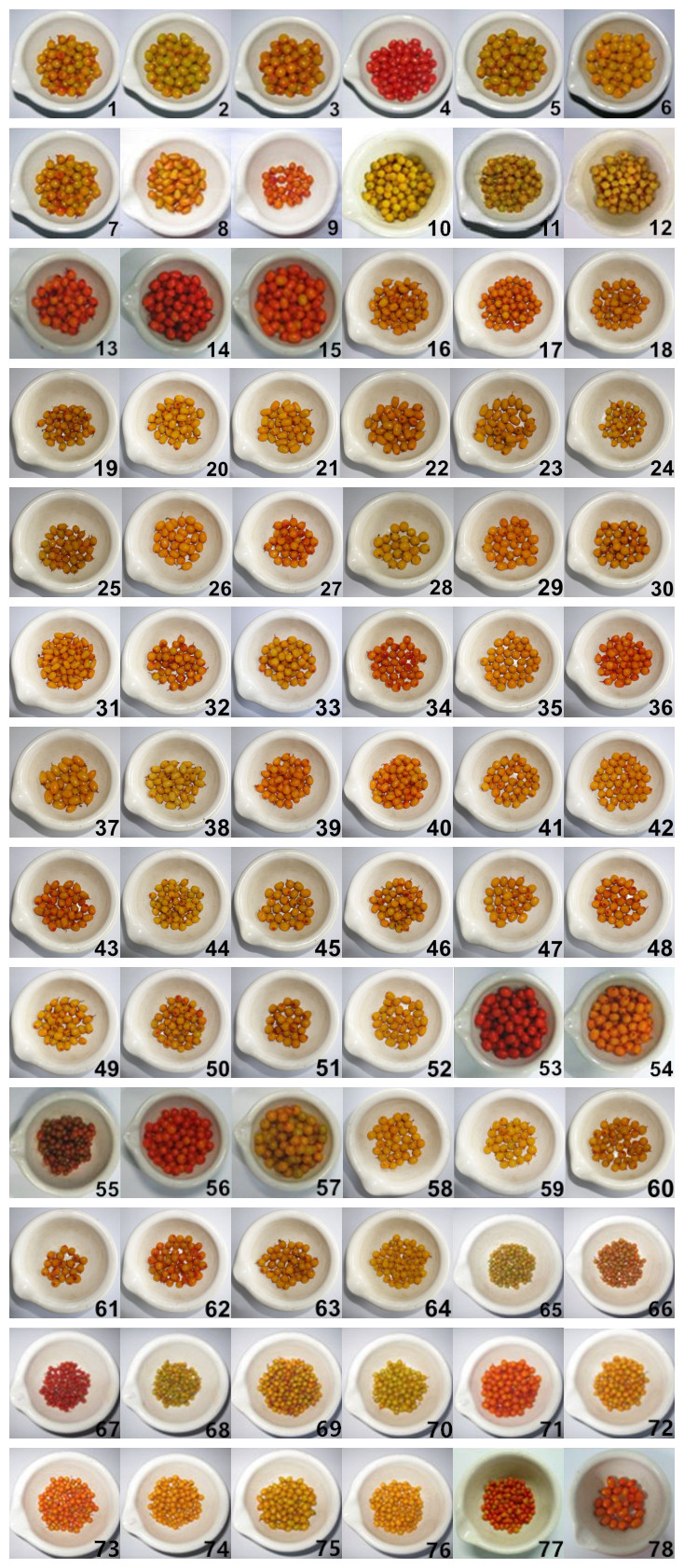

Supplement: S1 Fig — Numbers are the variety codes listed in Table 1. (TIF) [file pone.0230356.s001.tif]

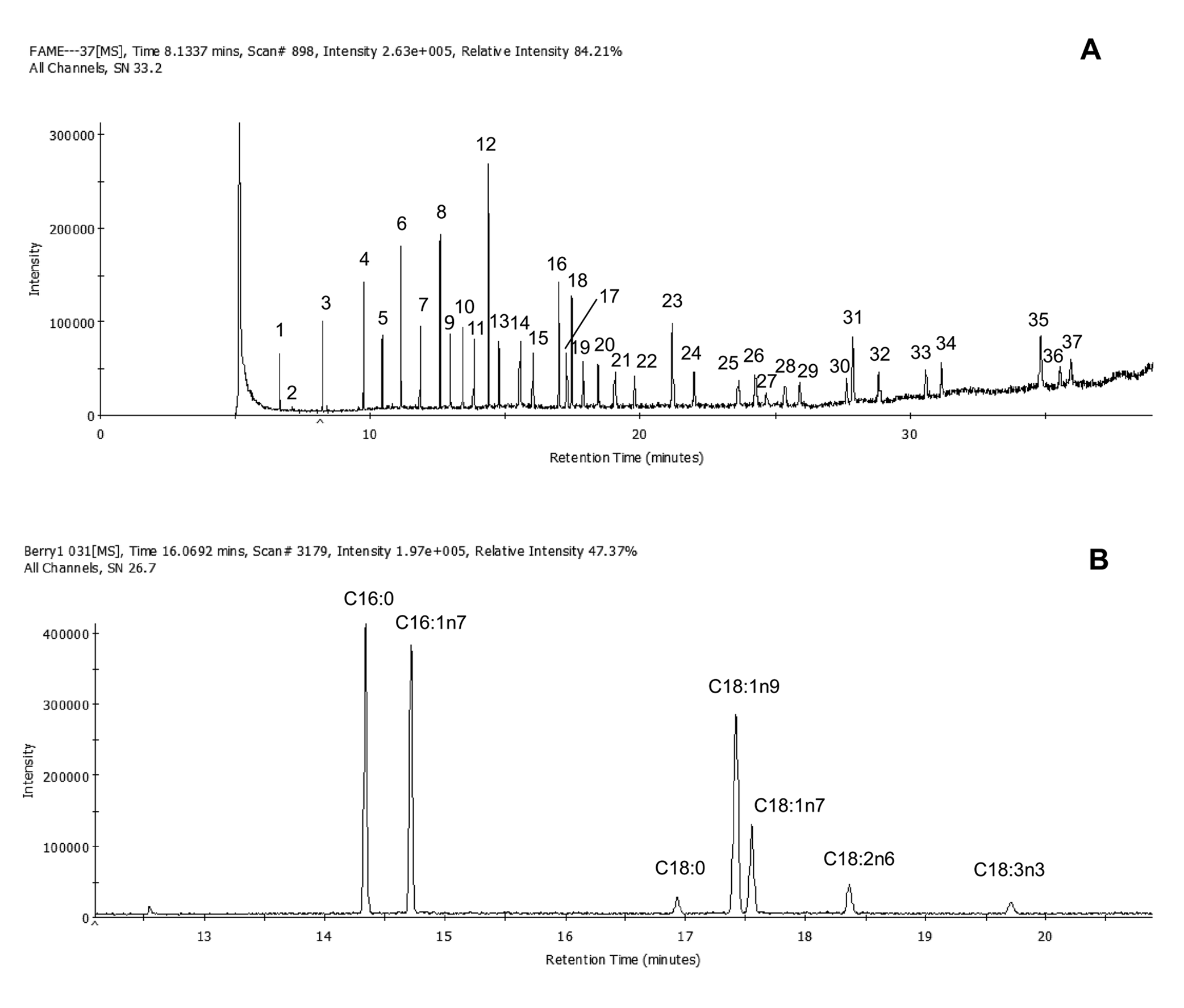

Supplement: S2 Fig — Total ion flow chromatography of 37 FAMEs Mix (A) and FAMEs in pulp oil in MHC (B). (TIF) [file pone.0230356.s002.tif]
