# Supplementary material for: Diversity in sea buckthorn (Hippophae rhamnoides L.) accessions with different origins based on morphological characteristics, oil traits, and microsatellite markers
Source: PLoS One. 2020 Mar 13;15(3):e0230356. doi: 10.1371/journal.pone.0230356 (PMC7069629; doi:10.1371/journal.pone.0230356)
Supplement: S1 Table — (DOCX) [file pone.0230356.s003.docx]

**S1 Table. Samples of sea buckthorn grouped according to different genetic backgrounds.**

| **Accession name (Abbreviation; Code)** | **Growth site in China** | **Origin** | **ssp.^a^** | **Pedigree/background information^b^** |
| --- | --- | --- | --- | --- |
| Zhuangyuanhuang (ZYH; 1); Wucifeng (WCF; 2); Liusha-1(LS1; 3); Siberia rumianes (SR; 4); Fangxiang (FX; 5); Yalishanda-12 (YLSD12; 6); Jiuyuehuang (JYH; 7); Nanren (NR; 8); Botanical garden (BG; 9) | Fuxin, Liaoning | Russia | M | selected from the seedlings or asexual reproduction materials of cultivars from Russia |
| Qiuyisike (QYSK; 78) | Qinghe, Xinjiang | Russia | M |  |
| MZ-14 (MZ14; 13); Shoudu (SD; 14); Fenlan (FL; 15) ; Aertaixin (AET; 16); Chengse (CS; 17); Chuyi (CY; 18); Hunjin (HJ; 19); Jinse (JS; 20); Juren (JR; 21); Xiangyang (XY; 22); Yousheng (YS; 23); Katuni (KTN, 24) | Suiling, Heilongjiang | Russia | M |  |
| Wulangemu (WLGM; 25) | Suiling, Heilongjiang | Mongolia | M | cultivar introduced from Mongolia |
| TF1 (TF1; 26); TF2-13 (TF2-13; 27); TF2-23 (TF2-23; 28); TF2-24 (TF2-24; 29); TF2-36 (TF2-36; 30); Suiji-1 (SJ-1; 31); Suiji-3 (SJ-3; 32); Suiji-4 (SJ-4; 33); HD-3 (HD3; 34); E10-6 (E10-6; 35); E10-34 (E10-34; 36); E10-42 (E10-42; 37); E10-47 (E10-47; 38); E13-00 (E13-00; 39); E13-10 (E13-10; 40); E13-11 (E13-11; 41); E13-14 (E13-14; 42); HS-1 (HS1; 43); HS-4 (HS4; 44); HS-9 (HS9; 45); HS-10 (HS10; 46); HS-12 (HS12; 47); HS-14 (HS14; 48); HS-18 (HS18; 49); HS-20 (HS20; 50); HS-22 (HS22; 51); Xin’e-1 (XE1; 52); Xin’e-2 (XE2; 53); Xin’e-3 (XE3; 54) | Suiling, Heilongjiang | China | M | seedling progenies of elite cultivars introduced from Russia |
| Zhongguoshaji (ZGSJ, 55) | Suiling, Heilongjiang | China | S | selected from the seedlings of ZGSJ*^n^* from different populations |
| Juda (JD; 65); Jianpingdahuang (JPDH; 66); Manhanci (MHC; 67); Zhongxiongyou (ZXY; 68) | Dongsheng, Inner Mongolia | China | S |  |
| Zhongguoshajiwild (ZGSJwild, 77) | Datong, Qinghai | China | S | domesticated from wild ZGSJ*^n^* |
| Zajiao-1 (ZJ1; 10); Zajiao-2 (ZJ2; 11); Zajiao-3 (ZJ3; 12) | Fuxin, Liaoning | China | H | hybrid progenies of elite cultivars introduced from Russia (♀) and native cultivars from ZGSJ*^n^* populations (♂) |
| EZ-4 (EZ4; 56); Za-56 (Za56; 57); Za1-2(Za1-2; 58); Za05-6 (Za05-6; 59); Za05-20 (Za05-20;60); Za05-21* (Za05-21; 61); Za4* (Za4; 62); Za13-19* (Za13-19; 63); Za13-25* (Za13-25; 64) | Suiling, Heilongjiang | China | H |  |
| Liaofuza (LFZ, 69); Zaciyou-1(ZCY1,70); Zaciyou-10 (ZCY10, 71); Zaciyou-12 (ZCY1, 72); Xinzaci-26 (XZC26, 73); Shiciyou-2 (SCY2, 74); Shiciyou-5 (SCY2, 75); Shiciyou-30 (SCY2, 76) | Dongsheng, Inner Mongolia | China | H |  |

*^a^* ssp., subspecies; M, ssp. *mongolica*; S, ssp. *sinensis*; H, hybrid (ssp. *mongolica* × ssp. *sinensis*).

*^b^* ZGSJ*^n^* = native Zhongguoshaji, representing all cultivars of ssp. *sinensis* in China.
