# Supplementary material for: Diversity in sea buckthorn (Hippophae rhamnoides L.) accessions with different origins based on morphological characteristics, oil traits, and microsatellite markers
Source: PLoS One. 2020 Mar 13;15(3):e0230356. doi: 10.1371/journal.pone.0230356 (PMC7069629; doi:10.1371/journal.pone.0230356)
Supplement: S2 Table — (DOCX) [file pone.0230356.s004.docx]

**S2 Table. Characterization of the hybrids of sea buckthorn accessions studied.**

| **Accession name** | **Abbrev.^a^** | **Collection site** | **Pedigree/Background information** | **Agronomic traits** |
| --- | --- | --- | --- | --- |
| Zajiao-1 | ZJ1 | Fuxin | Selected from hybrids of Qiuyisike (ssp. *mongolica*) × male plants of HF-88-02 (ssp. *sinensis*) | The crown is round and open without distinct trunk. No or few thorns, round yellow fruit, fruit stalk = 3.2 mm, weight of hundred-fruit = 45.5 g, fruit grain density = 70.4 grains/10cm, Vc = 80.4 mg/100g, β-carotene = 4.8 mg/100g, total flavone = 55.3 mg/100g, seed oil content = 9.4 %, susceptible to dried-shrink disease. |
| Zajiao-2 | ZJ2 | Fuxin | Selected from hybrids of Qiuyisike (ssp. *mongolica*) × male plants of HF-88-02 (ssp. *sinensis*) | The crown is open with distinct trunk. Height about 2.8 m, vigorous growth, no or few thorns, fruit stalk = 3.0 mm, round yellow fruit, weight of hundred-fruit = 58.0 g, fruit grain density = 74.6 grains/10cm, total flavone = 47.1 mg/100g, seed oil content = 10.6 %, mean fresh fruit yield per plant = 3.6 kg, resistance to dried-shrink disease. |
| Zajiao-3 | ZJ3 | Fuxin | Selected from hybrids of Qiuyisike (ssp. *mongolica*) × male plants of HF-88-02 (ssp. *sinensis*) | No or few thorns, round yellow fruit with red colour on top of it, weight of hundred-fruit = 37.6 g, fruit grain density = 75.0 grains/10 cm, Vc =81.2 mg/100g, seed oil content = 10.1 %, mean fresh fruit yield per plant = 4.2 kg, resistance to dried-shrink disease. |
| EZ-4 | EZ4 | Suiling | Suiji-1(ssp. *mongolica*) × male plants of MK-88-01 (ssp. *sinensis*) | Height about 2.0-2.5 m, few thorns, round yellow fruit, fruit stalk = 2.9 mm, fruit grain density = 65.0 grains/10cm, the number of thorns of annual branch = 0.3, weight of hundred-fruit = 52.1 g, mean fresh fruit yield per plant = 5.2 kg. |
| Za-56 | Za56 | Suiling | Alieyi (ssp. *mongolica*) × male plants of MK-88-01 (ssp. *sinensis*) | Height about 2.6 m, few thorns, round yellow fruit, fruit stalk = 3.8 mm, fruit grain density = 58.0 grains/10cm, the number of thorns of annual branch = 0.3, weight of hundred-fruit = 58.6 g, mean fresh fruit yield per plant = 5.5 kg. |
| Za1-2 | Za1-2 | Suiling | Wulangemu (ssp. *mongolica*) × male plants of Zhongguoshaji (Fengning population, ssp. *sinensis*) | Height about 2.5 m, few thorns, round yellow fruit, fruit stalk = 2.9 mm, fruit grain density = 58.4 grains/10cm, the number of thorns of annual branch = 0.3, weight of hundred-fruit = 42.8 g, mean fresh fruit yield per plant = 4.0 kg. |
| Za05-6 | Za05-6 | Suiling | Wulangemu (ssp. *mongolica*) × male plants of Zhongguoshaji (Fengning population, ssp. *sinensis*) | Height about 2.4 m, few thorns, round yellow fruit, fruit stalk = 2.7 mm, fruit grain density = 59.4 grains/10 cm the number of thorns of annual branch = 0.5, weight of hundred-fruit = 41.7 g, mean fresh fruit yield per plant = 4.2 kg. |
| Za05-20 | Za05-20 | Suiling | Wulangemu (ssp. *mongolica*) × male plants of Zhongguoshaji (Fengning population, ssp. *sinensis*) | Height about 2.5 m, few thorns, round yellow fruit with red color on top of it, fruit stalk = 3.2 mm, fruit grain density = 62.7 grains/10 cm the number of thorns of annual branch = 1.0, weight of hundred-fruit =49.6 g, mean fresh fruit yield per plant = 4.5 kg. |
| Za05-21 | Za05-21 | Suiling | Wulangemu (ssp. *mongolica*) × male plants of Zhongguoshaji (Fengning population, ssp. *sinensis*) | Height about 2.1 m, few thorns, round yellow fruit with red color on top of it, fruit stalk = 2.7 mm, fruit grain density = 65.4 grains/10 cm the number of thorns of annual branch = 0.4, weight of hundred-fruit = 42.8 g, mean fresh fruit yield per plant = 4.5 kg. |
| Za4 | Za4 | Suiling | Chuyi (ssp. *mongolica*) × male plants of MK-88-01 (ssp. *sinensis*) | Height about 4.3 m, the number of thorns of annual branch = 0.3, round orange fruit, weight of hundred-fruit = 46.3 g, mean fresh fruit yield per plant = 3.6 kg. |
| Za13-19 | Za13-19 | Suiling | Chuyi (ssp. *mongolica*) × male plants of MK-88-01 (ssp. *sinensis*) | Height about 2.8 m, the number of thorns of annual branch branch = 0.3, round yellow fruit, weight of hundred-fruit = 47.6 g, mean fresh fruit yield per plant = 3.3 kg. |
| Za13-25 | Za13-25 | Suiling | Chuyi (ssp. *mongolica*) × male plants of MK-88-01 (ssp. *sinensis*) | Height about 3.0 m, the number of thorns of annual branch = 0.9, round yellow fruit, weight of hundred-fruit = 49.3 g, mean fresh fruit yield per plant = 3.5 kg. |
| Liaofuza | LFZ | Dongsheng | Chuyi (ssp. *mongolica*) × male plants of Zhongguowucixiong (ssp. *sinensis*) | Few thorns, vigorous growth, round yellow fruit with red color on top of it, fruit grain density = 62-78 grains/10cm, the number of thorns of annual branch = 3, weight of hundred-fruit = 35.3 g, Vc = 178.6 mg/100g, β-carotene = 30.1 mg/100g, total flavone = 114.0 mg/100g, seed oil content = 12.0 %, resistance to dried-shrink disease. |
| Zaciyou-1 | ZCY1 | Dongsheng | Wulanshalin (ssp. *mongolica*) × male plants of Zhongguoshaji (Fengning population, ssp. *sinensis*) | Height about 1.7 m, few thorns, round yellow fruit, fruit stalk = 3.0 mm, the number of thorns of annual branch = 1.3, weight of hundred-fruit = 31.2 g, mean fresh fruit yield per plant = 3.6 kg. |
| Zaciyou-10 | ZCY10 | Dongsheng | Wulanshalin (ssp. *mongolica*) × male plants of Zhongguoshaji (Fengning population, ssp. *sinensis*) | Height about 2.4 m, few thorns, round orange fruit, fruit stalk = 2.7 mm, the number of thorns of annual branch = 0.3, weight of hundred-fruit = 30.8 g, mean fresh fruit yield per plant = 3.8 kg. |
| Zaciyou-12 | ZCY12 | Dongsheng | Wulanshalin (ssp. *mongolica*) × male plants of Zhongguoshaji (Fengning population, ssp. *sinensis*) | Height about 2.2 m, few thorns, round yellow fruit, fruit stalk = 2.0 mm, the number of thorns of annual branch = 0.9, weight of hundred-fruit = 30.6 g, mean fresh fruit yield per plant = 2.9 kg. |
| Xinzaci-26 | XZC26 | Dongsheng | Taiyang (ssp. *mongolica*) × male plants of Zhongguoshaji (Manhanshan population, ssp. *sinensis*) | Height about 1.7 m, few thorns, round orange fruit, fruit stalk = 1.6 mm, fruit grain density = 22.9 grains/10 cm, the number of thorns of annual branch = 0.7, weight of hundred-fruit = 25.4 g, mean fresh fruit yield per plant = 2.5 kg. |
| Shiciyou-2 | SCY2 | Dongsheng | Yousheng (ssp. *mongolica*) × male plants of Zhongguoshaji (Manhanshan population, ssp. *sinensis*) | Height about 1.6 m, few thorns, round yellow fruit, fruit stalk = 1.8 mm, fruit grain density = 30.0 grains/10 cm, the number of thorns of annual branch = 0.5, weight of hundred-fruit = 24.0 g, mean fresh fruit yield per plant = 2.6 kg. |
| Shiciyou-5 | SCY5 | Dongsheng | Yousheng (ssp. *mongolica*) × male plants of Zhongguoshaji (Manhanshan population, ssp. *sinensis*) | Height about 1.9 m, few thorns, round yellow fruit, fruit stalk = 2.7 mm, fruit grain density = 32.7 grains/10 cm the number of thorns of annual branch = 0.8, weight of hundred-fruit = 25.6 g, mean fresh fruit yield per plant = 2.5 kg. |
| Shiciyou-30 | SCY30 | Dongsheng | Chuyi (ssp. *mongolica*) × male plants of Zhongguoshaji (Manhanshan population, ssp. *sinensis*) | Height about 1.6 m, few thorns, round yellow fruit, fruit stalk = 1.8 mm, fruit grain density = 28.0 grains/10 cm the number of thorns of annual branch = 0.5, weight of hundred-fruit = 20.4 g, mean fresh fruit yield per plant = 2.0 kg. |

^a^ Abbrev., abbreviation.
