# Supplementary material for: Diversity in sea buckthorn (Hippophae rhamnoides L.) accessions with different origins based on morphological characteristics, oil traits, and microsatellite markers
Source: PLoS One. 2020 Mar 13;15(3):e0230356. doi: 10.1371/journal.pone.0230356 (PMC7069629; doi:10.1371/journal.pone.0230356)
Supplement: S3 Table — (DOCX) [file pone.0230356.s005.docx]

**S3 Table. Climatic conditions at different growth sites of sea buckthorn samples in China.**

| **Growth site** | **Latitude**  **(N)** | **Longitude**  **(E)** | **Altitude**  **(m)** | **Avg annual temp^a^ (°C)** | **Avg annual prec^b^ (mm)** | **Avg annual evap^c^ (mm)** | **Avg annual shin^d^ (h)** | **Annual frost-free days (no.)^e^** |
| --- | --- | --- | --- | --- | --- | --- | --- | --- |
| Fuxin, Liaoning | 42°26′ | 121°48′ | 380 | 7.2 | 497 | 1848 | 2550 | 144 |
| Suiling, Heilongjiang | 47°14′ | 127°06′ | 202 | 2.0 | 580 | 124 | 2656 | 127 |
| Dongsheng, Inner Mongolia | 39°40′ | 110°09′ | 1400 | 7.3 | 387.2 | 2257 | 3100 | 140 |
| Qinghe, Xinjiang | 46°40′ | 90°22′ | 1218 | 1.3 | 161 | 1495 | 2740 | 103 |
| Datong, Qinghai | 36°53′ | 101°35′ | 2800 | 4.9 | 523.3 | 1762.8 | 2553 | 120 |

^a^Avg annual temp = Annual average temperature.

^b^Avg annual prec = Annual average precipitation.

^c^ Avg annual evap = Annual average evaporation

^d^ Avg annual suns = Annual average sunshine hours

^e^Annual frost-free days (no.) = number of annual frost-free days.
