# Supplementary material for: Diversity in sea buckthorn (Hippophae rhamnoides L.) accessions with different origins based on morphological characteristics, oil traits, and microsatellite markers
Source: PLoS One. 2020 Mar 13;15(3):e0230356. doi: 10.1371/journal.pone.0230356 (PMC7069629; doi:10.1371/journal.pone.0230356)
Supplement: S4 Table — (DOCX) [file pone.0230356.s006.docx]

**S4 Table. Primer sequences, annealing temperature, and estimated allelic size of 23 SSR markers.**

| **Loci code** | **Forward primer (5′ to 3′)** | **Reverse primer (5′ to 3′)** | **Tm(°C)** | **Estimated allelic size (bp)** |
| --- | --- | --- | --- | --- |
| SB1 | CATGTAGCCCAGCAGTATTGAA | AGAGGGCAAGAAGGAGAAGAAG | 58 | 157 |
| SB2 | ATGTGGATTGGTTTAGCAACACT | ACTCGACAAAATCCCACTAAAGG | 56 | 159 |
| SB3 | GTCACAAGGGCTAATGTTACTGC | GCAATGGGTTTAAGGGTATGAGT | 60 | 131 |
| SB4 | ACCTCTAGGATTTCACTTTCTTGT | GGTCGTATCCATATGACACTCGT | 54 | 150 |
| SB5 | AGTCTTCGGATAGGTCGTCATC | CAAAGAACCTAACCCACTGCTAC | 60 | 125 |
| SB6 | GACAATTTTTCCCATGTTGTAGC | GAAATTTAAAATCCCAGTTCATGG | 56 | 152 |
| SB7 | TGGAAATAGTAACAATGGGCAGT | GTTCACGAATTTCATCTGCTTCT | 56 | 125 |
| SB8 | AGAGATAGTGGAGTAAGTCCCGC | CAAATCGAAGAAGTGATCAGGTTA | 60 | 147 |
| SB9 | CACCATCATAATACCCAAAATGG | TTGTATTTGTCGCATTGTTTCCT | 54 | 139 |
| SB10 | ATAAACAAATGGGCAAATCAGTG | TTCTTCTTCATCACCACAATCCT | 60 | 155 |
| SB11 | TGTATACTCCCCACCAACAACAT | GTGATGACATCGTATCCAGATCA | 58 | 124 |
| SB12 | AGAGATCGAGTCTTGGCTCTGTA | GAAGGACTCCACTTGGATGACTA | 60 | 154 |
| SB13 | AAGCTGGAGGAACTAGAGTAGCC | TGATATTTTTGATTCAGGGATCG | 60 | 135 |
| SB14 | GATTTGCTTTTCCTTAAAGCTGG | ATCAAAAGAAGGAAACCCAGAAG | 60 | 157 |
| SB15 | AGGTCTGATCATTGACATTTGCT | ATCACGGGACCCTATATCATACC | 60 | 154 |
| SB16 | TTGTTCAATCTAGGAGGCAGAAG | TGGTAGTGTTGGTTCAACAAGTG | 60 | 169 |
| SB17 | GTTTCGGATTCAAAGAACAAGAG | GCATATGCAACCTATGAACCAAC | 56 | 156 |
| SB18 | GAGTTAATCGATGGATAATGATTG | TTCCTTCATTTGAAACCTCATTC | 55 | 160 |
| SB19 | ATTATTTGCCCATGCTGACTAAC | CAGCTTCTTCTACACCCAAGCTA | 60 | 154 |
| SB20 | TGTTCTGCTAAGAACTTCAACCA | TTCAGAAATTACTGCAGACATGA | 58 | 157 |
| SB21 | CGGCTGAGATGTAGTAGAAGCTG | TCATCATCGTCATCCTCACC | 59 | 160 |
| SB22 | TCAGTCATATTACAATTCACACGG | TCACAACCTTAACATTTCAACCA | 59 | 152 |
| SB23 | TGAAGATCAACAACACTACCACC | AAGAATCTGTGCCTTGAACTTGA | 60 | 137 |
