# Supplementary material for: Diversity in sea buckthorn (Hippophae rhamnoides L.) accessions with different origins based on morphological characteristics, oil traits, and microsatellite markers
Source: PLoS One. 2020 Mar 13;15(3):e0230356. doi: 10.1371/journal.pone.0230356 (PMC7069629; doi:10.1371/journal.pone.0230356)
Supplement: S5 Table — (DOCX) [file pone.0230356.s007.docx]

**S5 Table. Descriptive statistics for morphological traits of berries and seeds among the sea buckthorn accessions studied.**

| **Character** | **Min**^a^ | **Max**^b^ | **Mean ± SD**^c^ | **CV**^d^ **(%)** |
| --- | --- | --- | --- | --- |
| HBW (g) | 8.52 | 69.74 | 40.68 **±** 15.92 | 39.12 |
| BTD (mm) | 5.54 | 10.80 | 7.85 **±** 1.20 | 15.25 |
| BLD (mm) | 4.83 | 14.25 | 9.76 **±** 2.23 | 22.87 |
| BSI (%) | 0.85 | 1.89 | 1.24 **±** 0.23 | 18.55 |
| HSW (g) | 0.61 | 2.19 | 1.45 **±** 0.35 | 24.33 |
| SL (mm) | 2.98 | 7.43 | 5.38 **±** 1.03 | 19.06 |
| SW (mm) | 2.00 | 3.49 | 2.65 **±** 0.30 | 11.37 |
| ST (mm) | 1.54 | 2.73 | 1.93 **±** 0.22 | 11.50 |

^a^ Minimum value.

^b^ Maximum value.

^c^ Standard deviation.

^d^ Coefficient of variation expressed in percentage.
