# Supplementary material for: Diversity in sea buckthorn (Hippophae rhamnoides L.) accessions with different origins based on morphological characteristics, oil traits, and microsatellite markers
Source: PLoS One. 2020 Mar 13;15(3):e0230356. doi: 10.1371/journal.pone.0230356 (PMC7069629; doi:10.1371/journal.pone.0230356)
Supplement: S7 Table — (DOCX) [file pone.0230356.s009.docx]

**S7 Table. Fruit traits and Vc contents of large berry accessions of sea buckthorn in two experimental fields (located in Suiling and Dengkou).**

|  | **HBW (g)** | | | **BLD (cm)** | | **BTD (cm)** | | **BSI** | | **Vc (mg·kg^-1^)** | |
| --- | --- | --- | --- | --- | --- | --- | --- | --- | --- | --- | --- |
| **Accession name** | **Russia** | **Suiling** | **Dengkou** | **Suiling** | **Dengkou** | **Suiling** | **Dengkou** | **Suiling** | **Dengkou** | **Russia** | **Suiling** |
| Aertai | 50 | 67.59 ± 7.47 | 42.25 ± 2.53 | 1.27 ± 0.09 | 0.96 ± 0.03 | 0.85 ± 0.03 | 0.72 ± 0.02 | 1.50 ± 0.05 | 1.33 ± 0.04 | 470 | 342.6 ± 28.35 |
| Chengse | 60 | 50.87 ± 2.87 | 38.34 ± 2.77 | 1.05 ± 0.04 | 0.97 ± 0.03 | 0.86 ± 0.06 | 0.79 ± 0.02 | 1.22 ± 0.04 | 1.23 ± 0.04 | 3300 | 1770.3 ± 216.73 |
| Chuyi | 90 | 65.17 ± 4.81 | 63.85 ± 5.38 | 1.28 ± 0.08 | 1.27 ± 0.05 | 0.86 ± 0.04 | 0.86 ± 0.03 | 1.49 ± 0.06 | 1.48 ± 0.05 | 1340 | 963.4 ± 128.55 |
| Hunjin | 70 | 49.2 ± 2.39 | 32.87 ± 1.94 | 1.06 ± 0.04 | 0.98 ± 0.04 | 0.82 ± 0.02 | 0.81 ± 0.04 | 1.30 ± 0.06 | 1.21 ± 0.05 | 1330 | 954.8 ± 134.69 |
| Jinse | 80 | 53.90 ± 2.87 | 51.71 ± 2.25 | 1.12 ± 0.06 | 1.26 ± 0.07 | 0.85 ± 0.03 | 0.83 ± 0.04 | 1.33 ± 0.05 | 1.52 ± 0.07 | 1150 | 716.6 ± 118.22 |
| Juren | 80 | 60.25 ± 4.83 | 45.25 ± 1.64 | 1.32 ± 0.07 | 1.29 ± 0.06 | 0.81 ± 0.03 | 0.67 ± 0.04 | 1.62 ± 0.07 | 1.93 ± 0.09 | 1570 | 993.9 ± 153.65 |
| Xiangyang | 50 | 65.35 ± 5.50 | 57.21 ± 4.26 | 1.42 ± 0.09 | 1.35 ±0.07 | 0.89 ± 0.04 | 0.87 ± 0.03 | 1.59 ± 0.05 | 1.55 ± 0.06 | 1255 | 896.3 ± 158.64 |
| Yousheng | 80 | 55.59 ± 3.65 | 42.35 ± 1.89 | 1.27 ± 0.05 | 1.02 ±0.05 | 0.79 ± 0.04 | 0.80 ± 0.02 | 1.61 ± 0.09 | 1.28 ± 0.04 | 1180 | 975.3 ± 170.25 |
| Katuni | 40 | 38.33 ± 1.80 | 38.35 ± 1.43 | 0.95 ± 0.03 | 0.73 ± 0.02 | 0.77 ± 0.03 | 0.76 ± 0.02 | 1.24 ± 0.05 | 0.96 ± 0.03 | 700 | 624.3 ± 125.77 |
| Wulangemu | 60 | 43.01 ± 3.19 | 41.04 ± 2.23 | 0.96 ± 0.03 | 1.12 ± 0.04 | 0.81 ± 0.03 | 0.80 ± 0.02 | 1.19 ± 0.07 | 1.40 ± 0.04 | 1180 | 820.3 ± 189.48 |
| Suiji-1 | – | 48.54 ± 2.53 | 42.27 ± 3.35 | 1.22 ± 0.05 | 1.24 ± 0.06 | 0.75 ± 0.02 | 0.81 ± 0.03 | 1.62 ± 0.05 | 1.53 ± 0.05 | – | 685.4 ± 143.78 |
| Zhongguoshaji  (control) | – | – | 18.83 ± 1.36 | – | 0.53 ± 0.02 | – | 0.60 ± 0.02 | – | 0.88 ± 0.03 | – | 11350 ± 1884.65 |

–, no information. Suiji-1 accession is the seedling progeny of Chuyi. Zhongguoshaji is native to China and grown in Dengkou.
