# Supplementary material for: Diversity in sea buckthorn (Hippophae rhamnoides L.) accessions with different origins based on morphological characteristics, oil traits, and microsatellite markers
Source: PLoS One. 2020 Mar 13;15(3):e0230356. doi: 10.1371/journal.pone.0230356 (PMC7069629; doi:10.1371/journal.pone.0230356)
Supplement: Attachment — Submitted filename: PONE-D-19-17567_reviewer.pdf [file pone.0230356.s011.pdf]

PLOS ONE

Diversity in sea buckthorn (Hippophae rhamnoides L.) varieties with different origins based on morphological characteristics, oil traits, and microsatellite markers

--Manuscript Draft--

Manuscript Number:	PONE-D-19-17567
Article Type:	Research Article
Full Title:	Diversity in sea buckthorn (Hippophae rhamnoides L.) varieties with different origins based on morphological characteristics, oil traits, and microsatellite markers
Short Title:	Diversity in sea buckthorn varieties based on morphological characteristics, oil traits, and SSR markers
Corresponding Author:	Chengjiang Ruan<br>Dalian Medical University<br>Dalian, CHINA
Keywords:	diversity analysis; morphological characteristics; SSR markers; biochemical traits; fatty acid composition
Abstract:	Sea buckthorn (Hippophae rhamnoides) is an ecologically and economically important species. Here, we assessed the diversity of 78 varieties cultivated in northern China using 8 agronomic characteristics, oil traits (including oil content and fatty acid composition) in seeds and pulp/peel, and SSR markers at 20 loci. The 78 varieties included 52 from ssp. mongolica, 6 from ssp. sinensis, and 20 hybrids. To assess the phenotypic diversity of these varieties, 8 agronomic fruit traits were recorded and analyzed using principal component analysis (PCA). The first two PCs accounted for approximately 78% of the variation among varieties. The oil contents were higher in pulp/peel (3.46-38.56%) than in seeds (3.88-8.82%), especially in ssp. mongolica varieties. The polyunsaturated fatty acids (PUFA) ratio was slightly lower in seed oil of hybrids (76.06%) than in ssp. mongolica (77.66%) and higher than in ssp. sinensis (72.22%). The monounsaturated fatty acids (MUFA) ratio of pulp/peel oil of ssp. sinensis (57.00%) was highest, and that of ssp. mongolica (51.00%) was approximately equal to the ratio in the hybrids (51.20%). Using canonical correspondence analysis (CCA), we examined the correlation between agronomic traits and oil characteristics in pulp/peel and seeds, respectively. Oil traits in pulp/peel from different origins were correlated with morphological groupings (r = 0.8725, p = 0.0000). To assess the genotypic diversity, 20 SSR markers were used among the 78 varieties with 59 polymorphic amplified fragments obtained and an average PIC value of 0.2725. All varieties were classified into two groups based on the UPGMA method. The varieties of ssp. sinensis and ssp. mongolica were genetically distant. Seven hybrid varieties were close to ssp. sinensis varieties whereas the others were close to ssp. mongolica varieties. The 8 agronomic traits, oil characters in seed and pulp/peel oils, and 20 SSR markers successfully distinguished the 78 varieties. These results will be valuable for cultivar identification and genetic diversity analysis in cultivated sea buckthorn.
Order of Authors:	He Li
	Chengjiang Ruan
	Jian Ding
	Jingbin Li
	Li Wang
	Xingjun Tian
Additional Information:	
Question	Response
Financial Disclosure	This research was financially supported by the Natural Science Foundation of China (NSFC)(Grant No. 31100489), which was received by He Li.

Powered by Editorial Manager® and ProduXion Manager® from Aries Systems Corporation

Enter a financial disclosure statement that describes the sources of funding for the work included in this submission. Review the submission guidelines for detailed requirements. View published research articles from *PLOS ONE* for specific examples.<br><br>This statement is required for submission and **will appear in the published article** if the submission is accepted. Please make sure it is accurate.<br><br>**Unfunded studies**<br>Enter: *The author(s) received no specific funding for this work.*<br><br>**Funded studies**<br>Enter a statement with the following details:<br>• Initials of the authors who received each award<br>• Grant numbers awarded to each author<br>• The full name of each funder<br>• URL of each funder website<br>• Did the sponsors or funders play any role in the study design, data collection and analysis, decision to publish, or preparation of the manuscript?<br>• **NO** - Include this sentence at the end of your statement: *The funders had no role in study design, data collection and analysis, decision to publish, or preparation of the manuscript.*<br>• **YES** - Specify the role(s) played.<br><br>* typeset	https://isisn.nsfc.gov.cn/egrantweb/
**Competing Interests**<br><br>Use the instructions below to enter a competing interest statement for this submission. On behalf of all authors, disclose any competing interests that could be perceived to bias this work—acknowledging all financial support and any other relevant financial or non-financial competing interests.<br><br>This statement **will appear in the published article** if the submission is accepted. Please make sure it is accurate. View published research articles from *PLOS ONE* for specific examples.	The authors have declared that no competing interests exist.

**NO authors have competing interests**<br><br>Enter: *The authors have declared that no competing interests exist.*<br><br>**Authors with competing interests**<br><br>Enter competing interest details beginning with this statement:<br><br>*I have read the journal's policy and the authors of this manuscript have the following competing interests: [insert competing interests here]*<br><br>* typeset	
**Ethics Statement**<br><br>Enter an ethics statement for this submission. This statement is required if the study involved:<br><br>• Human participants<br>• Human specimens or tissue<br>• Vertebrate animals or cephalopods<br>• Vertebrate embryos or tissues<br>• Field research<br><br>Write "N/A" if the submission does not require an ethics statement.<br><br>General guidance is provided below. Consult the submission guidelines for detailed instructions. **Make sure that all information entered here is included in the Methods section of the manuscript.**	N/A

**Format for specific study types**<br><br>**Human Subject Research (involving human participants and/or tissue)**<br>• Give the name of the institutional review board or ethics committee that approved the study<br>• Include the approval number and/or a statement indicating approval of this research<br>• Indicate the form of consent obtained (written/oral) or the reason that consent was not obtained (e.g. the data were analyzed anonymously)<br><br>**Animal Research (involving vertebrate animals, embryos or tissues)**<br>• Provide the name of the Institutional Animal Care and Use Committee (IACUC) or other relevant ethics board that reviewed the study protocol, and indicate whether they approved this research or granted a formal waiver of ethical approval<br>• Include an approval number if one was obtained<br>• If the study involved *non-human primates*, add *additional details* about animal welfare and steps taken to ameliorate suffering<br>• If anesthesia, euthanasia, or any kind of animal sacrifice is part of the study, include briefly which substances and/or methods were applied<br><br>**Field Research**<br><br>Include the following details if this study involves the collection of plant, animal, or other materials from a natural setting:<br>• Field permit number<br>• Name of the institution or relevant body that granted permission	
**Data Availability**<br><br>Authors are required to make all data underlying the findings described fully available, without restriction, and from the time of publication. PLOS allows rare exceptions to address legal and ethical concerns. See the PLOS Data Policy and FAQ for detailed information.	Yes - all data are fully available without restriction

A Data Availability Statement describing where the data can be found is required at submission. Your answers to this question constitute the Data Availability Statement and **will be published in the article**, if accepted.<br><br>**Important:** Stating 'data available on request from the author' is not sufficient. If your data are only available upon request, select 'No' for the first question and explain your exceptional situation in the text box.<br><br>Do the authors confirm that all data underlying the findings described in their manuscript are fully available without restriction?	
**Describe where the data may be found in full sentences. If you are copying our sample text, replace any instances of XXX with the appropriate details.**<br><br>• If the data are **held or will be held in a public repository**, include URLs, accession numbers or DOIs. If this information will only be available after acceptance, indicate this by ticking the box below. For example: *All XXX files are available from the XXX database (accession number(s) XXX, XXX.)*.<br>• If the data are all contained **within the manuscript and/or Supporting Information files**, enter the following: *All relevant data are within the manuscript and its Supporting Information files.*<br>• If neither of these applies but you are able to provide **details of access elsewhere**, with or without limitations, please do so. For example:<br><br>*Data cannot be shared publicly because of [XXX]. Data are available from the XXX Institutional Data Access / Ethics Committee (contact via XXX) for researchers who meet the criteria for access to confidential data.*<br><br>*The data underlying the results presented in the study are available from (include the name of the third party*	All relevant data are within the manuscript and its Supporting Information files.

and contact information or URL).<br>• This text is appropriate if the data are owned by a third party and authors do not have permission to share the data.<br><br>* typeset	
Additional data availability information:	

Powered by Editorial Manager® and ProduXion Manager® from Aries Systems Corporation

Manuscript
Click here to download Manuscript manuscript_He Li_20190620.docx

Diversity in sea buckthorn (*Hippophae rhamnoides* L.) varieties with different origins based on morphological characteristics, oil traits, and microsatellite markers

He Li[1,2], Chengjiang Ruan[2]*, Jian Ding[2], Jingbin Li[2], Li Wang[2], Xingjun Tian[1,3]*

[1] School of Life Science, Nanjing University, Nanjing, P.R. China

[2] Key Laboratory of Biotechnology and Bioresources Utilization, Dalian Minzu University, Dalian, P.R. China

[3] Co-Innovation Center for Sustainable Forestry in Southern China, Nanjing Forestry University, Nanjing, P.R. China

* Corresponding authors

E-mail: ruan@dlnu.edu.cn (CR); tianxj@nju.edu.cn (XT)

Abstract

Sea buckthorn (*Hippophae rhamnoides*) is an ecologically and economically important species. Here, we assessed the diversity of 78 varieties cultivated in northern China using 8 agronomic characteristics, oil traits (including oil content and fatty acid composition) in seeds and pulp/peel, and SSR markers at 20 loci. The 78 varieties included 52 from ssp. *mongolica*, 6 from ssp. *sinensis*, and 20 hybrids. To assess the phenotypic diversity of these varieties, 8 agronomic fruit traits were recorded and analyzed using principal component analysis (PCA). The first two PCs accounted for approximately 78% of the variation among varieties. The oil contents were higher in pulp/peel (3.46-38.56%) than in seeds (3.88-8.82%), especially in ssp. *mongolica* varieties. The polyunsaturated fatty acids (PUFA) ratio was slightly lower in seed oil of hybrids (76.06%) than in ssp. *mongolica* (77.66%) and higher than in ssp. *sinensis* (72.22%). The monounsaturated fatty acids (MUFA) ratio of pulp/peel oil of ssp. *sinensis* (57.00%) was highest, and that of ssp. *mongolica* (51.00%) was approximately equal to the ratio in the hybrids (51.20%). Using canonical correspondence analysis (CCA), we examined the correlation between agronomic traits and oil characteristics in pulp/peel and seeds, respectively. Oil traits in pulp/peel from different origins were correlated with morphological groupings (r = 0.8725, p = 0.0000). To assess the genotypic diversity, 20 SSR markers were used among the 78 varieties with 59 polymorphic amplified fragments obtained and an average PIC value of 0.2725. All varieties were classified into two groups based on the UPGMA method. The varieties of ssp. *sinensis* and ssp. *mongolica* were genetically distant. Seven hybrid varieties were

close to ssp. *sinensis* varieties whereas the others were close to ssp. *mongolica* varieties. The 8 agronomic traits, oil characters in seed and pulp/peel oils, and 20 SSR markers successfully distinguished the 78 varieties. These results will be valuable for cultivar identification and genetic diversity analysis in cultivated sea buckthorn.

Introduction

Sea buckthorn (*Hippophae rhamnoides* L.) is a winter hardy shrub that is naturally distributed throughout Asia and Europe. It is an economically valuable species, divided into eight subspecies. Of them, the ssp. *sinensis* and *mongolica* mainly distributed in Asia where they are abundant and commercially cultivated [1−2]. The fruits of sea buckthorn are rich in a variety of phytochemicals with physiological properties, such as lipids, carotenoids, ascorbic acid, tocopherols, and flavonoids [3−7]. The main applications for the fruits include food, cosmetics, and pharmaceutical products [8−10]. One of the most requested products for therapeutic practices is sea buckthorn oil, which is extracted from both seeds and pulp. The applications of sea buckthorn oils include healing of the skin, mucosa, and immune systems, especially in cancer and cardiovascular disease therapy [11−13].

Two important parameters in analyzing sea buckthorn oil quality are oil content and fatty acid composition (referred to here as ‘oil traits’ for simplicity). Sea buckthorn seed and pulp/peel oils are considered the most valuable products of the berries with a unique fatty acid (FA) composition [14]. The seed oil contains omega-3 (α-linolenic acid) and omega-6 (linoleic acid) FAs, and the pulp/peel oil is characterized by a high

concentration of FAs from the omega-7 group (e.g., palmitoleic acid). The seed oil is rich in unsaturated fatty acids (commonly 30-40% linoleic acid and 20-35% linolenic acid) [14]. The soft parts (pulp and peel) of the berries have a FA composition that differs from the seeds that is characterized by a high level of palmitoleic acid (16–54%), which is very uncommon in plants. The oil traits of sea buckthorn berries varies greatly according to their origin, based on the climatic and geological conditions of the growing areas [15].

Sea buckthorn is resistant to cold, drought, salt and alkali. The vigorous vegetative reproduction and the strong, complex root system with nitrogen-fixing nodules make it an optimal pioneer plant for soil and water conservation. For these reasons, sea buckthorn was cultivated widely in arid and semiarid areas of China [16]. Due to small berries and thorns of native cultivars (ssp. *sinensis*), which have little economic value, the breeding of sea buckthorn has undergone different stages of development in China, such as introduction, domestication, seedling selection and artificial hybridization for elite varieties. The cultivars of ssp. *mongolica* (introduced from Russia and Mongolia), ssp. *sinensis* (China origin) and hybrids (ssp. *mongolica* × ssp. *sinensis*) are abundant in northern China [17]. However, as a perennial woody plant, traditional cross breeding that takes a long time and has low efficiency cannot meet the needs of modern production in sea buckthorn. It is essential for economic production to utilize MAB (molecular marker-assisted breeding) in sea buckthorn, especially to breed those varieties associated with desirable oil traits. An essential step in this process is the genetic analysis of sea buckthorn germplasm. At present, molecular markers are mainly

used for the analysis of genetic diversity, the taxonomic and geographic origin of cultivars, sex determination and population genetic structure in sea buckthorn [17−19]. SSR (simple sequence repeat, microsatellite) markers, with 1- to 6-bp DNA regions repeated in tandem, have been used in these analysis for their advantages of codominance, random distribution throughout the genome, easy detection, and high polymorphism and reproducibility [20]. Currently, an increasing number of microsatellite markers are being developed in sea buckthorn using high-throughput sequencing techniques for transcriptome datasets (RNA-Seq), which have become valuable resources for SSR discovery [17, 21].

The aim of this study is to report the phenotypic characteristics and oil traits in pulp/peel and seeds, and genetic diversity of the 78 sea buckthorn varieties in northern China, providing the identification foundation for MAB in sea buckthorn.

Materials and methods

Plant materials

Berries and leaves of 78 sea buckthorn varieties were collected from the end of July to mid-September in 2015. Table 1 summarizes information on the plant materials. Three research institutes located in northern China, the Institute of Selection and Breeding of *Hippophae* (42°26′N, 121°28′E; 380 m) in Fuxin, the Research Institute of Berry (47°14′N, 127°06′E; 202 m) in Suiling and the Jiuchenggong Breeding Base of Sea Buckthorn (39°40′N, 110°09′E; 1400 m) in Dongsheng, provided 76 varieties (Fig 1). The other two elite varieties, Quyisike and Zhongguoshaji^{wild}, were harvested from

cultivated fields in Qinghe (46°40′N, 90°22′E; 1218 m) and Datong (36°53′N, 101°35′E; 2800 m) (Fig 1).

Fig 1. Five cultivated lands of the 78 sea buckthorn varieties used in this study.

Table 1. ~~Sampl~~[illegible] sea buckthorn grouped according to ~~different genetic backgrounds.~~

No.	Accession name	Abbrev.[a]	Origin	ssp.[b]	No.	Accession name	Abbrev.[a]	Origin	ssp.[b]
1	Zhuangyuanhuang	ZYH	Fuxin	M	40	E13-10	E13-10	Suiling	M
2	Wucifeng	WCF	Fuxin	M	41	E13-11	E13-11	Suiling	M
3	Liusha-1	LS1	Fuxin	M	42	E13-14	E13-14	Suiling	M
4	Siberia rumianes	SR	Fuxin	M	43	HS-1	HS1	Suiling	M
5	Fangxiang	FX	Fuxin	M	44	HS-4	HS4	Suiling	M
6	Yalishanda-12	YLSD12	Fuxin	M	45	HS-9	HS9	Suiling	M
7	Jiuyuehuang	JYH	Fuxin	M	46	HS-10	HS10	Suiling	M
8	Nanren	NR	Fuxin	M	47	HS-12	HS12	Suiling	M
9	Botanical garden	BG	Fuxin	M	48	HS-14	HS14	Suiling	M
10	Zajiao-1	ZJ1	Fuxin	H	49	HS-18	HS18	Suiling	M
11	Zajiao-2	ZJ2	Fuxin	H	50	HS-20	HS20	Suiling	M
12	Zajiao-3	ZJ3	Fuxin	H	51	HS-22	HS22	Suiling	M
13	MZ-14	MZ14	Suiling	M	52	Xin’e-1	XE1	Suiling	M
14	Shoudu	SD	Suiling	M	53	Xin’e-2	XE2	Suiling	M
15	Fenlan	FL	Suiling	M	54	Xin’e-3	XE3	Suiling	M

16	Aertai	AET	Suiling	M	55	Zhongguoshaji	ZGSJ	Suiling	S
17	Chengse	CS	Suiling	M	56	EZ-4	EZ4	Suiling	H
18	Chuyi	CY	Suiling	M	57	Za-56	Za56	Suiling	H
19	Hunjin	HJ	Suiling	M	58	Za1-2	Za1-2	Suiling	H
20	Jinse	JS	Suiling	M	59	Za05-6	Za05-6	Suiling	H
21	Juren	JR	Suiling	M	60	Za05-20	Za05-20	Suiling	H
22	Xiangyang	XY	Suiling	M	61	Za05-21	Za05-21	Suiling	H
23	Yousheng	YS	Suiling	M	62	Za4	Za4	Suiling	H
24	Katuni	KTN	Suiling	M	63	Za13-19	Za13-19	Suiling	H
25	Wulangemu	WLGM	Suiling	M	64	Za13-25	Za13-25	Suiling	H
26	TF1	TF1	Suiling	M	65	Juda	JD	Dongsheng	S
27	TF2-13	TF2-13	Suiling	M	66	Jianpingdahuang	JPDH	Dongsheng	S
28	TF2-23	TF2-23	Suiling	M	67	Manhanci	MHC	Dongsheng	S
29	TF2-24	TF2-24	Suiling	M	68	Zhongxiongyou	ZXY	Dongsheng	S
30	TF2-36	TF2- 36	Suiling	M	69	Liaofuza	LFZ	Dongsheng	H
31	Suiji-1	SJ-1	Suiling	M	70	Zaciyou-1	ZCY1	Dongsheng	H
32	Suiji-3	SJ-3	Suiling	M	71	Zaciyou-10	ZCY10	Dongsheng	H
33	Suiji-4	SJ-4	Suiling	M	72	Zaciyou-12	ZCY12	Dongsheng	H
34	HD-3	HD3	Suiling	M	73	Xinzaci-26	XZC26	Dongsheng	H
35	E10-06	E10-06	Suiling	M	74	Shiciyou-2	SCY2	Dongsheng	H
36	E10-34	E10-34	Suiling	M	75	Shiciyou-5	SCY5	Dongsheng	H
37	E10-42	E10-42	Suiling	M	76	Shiciyou-30	SCY30	Dongsheng	H

38	E10-47	E10-47	Suiling	M	77	Zhongguoshaji*wild*	ZGSJ*wild*	Datong	S
39	E13-00	E13-00	Suiling	M	78	Qiuyisike	QYSK	Qinghe	M

[a] Abbrev., abbreviation.

[b] ssp., subspecies; M, ssp. *mongolica*; S, ssp. *sinensis*; H, hybrid (ssp. *mongolica* × ssp. *sinensis*).

Morphological characteristics of fruit

Hundred berry weight (HBW) was the weight of 100 fresh berries after they were picked from bushes. Hundred seed weight (HSW) was the weight of 100 seeds after air drying at room temperature (25 °C) for 2 weeks [22]. There were three replicates for each measurement. The transverse and longitudinal diameters of berries (BTD and BLD) and the length, width and thickness of seeds (SL, SW and ST) were measured by micrometer calipers with over 20 measurements for each, on average. The berry shape indices (BSI) were estimated by the ratio of BLD to BTD.

Oil extraction and FA analysis in seeds and pulp/peel

The methods of lipid extraction, transesterification (methylation) and purification of methyl esters of the lipid extracts were described by Yang and Kallio [15]. Briefly, the seeds and pulp isolated from freeze-dried berries and lipids from the samples were extracted with chloroform/methanol (2:1, v/v) with mechanical homogenization of the tissues. The purified oils were filtered before the solvent was removed on a rotary evaporator. The lipids were weighed, and the oil contents (percentages) in seeds and pulp/peel were calculated. Each sample was analyzed three times. Lipids were stored

in chloroform at −20 °C until analysis.

The oil (10 mg) was transesterified by sodium methoxide catalysis [15, 23]. It was dissolved in sodium-dried diethyl ether (1ml) and methyl acetate (20 μl). Then 1 M sodium methoxide in dry methanol (20 μl) was added, and the solution was agitated briefly and set still for 5 min at room temperature. The reaction is stopped by adding a saturated solution of oxalic acid in diethyl ether (30 μl) with brief agitation. The mixture is centrifuged at 1500 g for 2 min and the supernatant was dried in a gentle stream of nitrogen. Fresh hexane (1 ml) was added and the solution was filtered with microporous filtering films (0.22 μm) for analysis.

FAMEs were analyzed with a gas chromatography-tandem mass spectrometry (GC/MS/MS) system (model AxION® iQT™, PekinElmer, Shelton, CT, USA). Chromatographic separation was achieved using a DB-23 capillary column (60 m × 0.25 mm × 0.25μm; Agilent Technologies, Santa Clara, CA, USA) with the following temperature program: initial temperature 50 °C, hold for 1 min, heated to 175 °C at 25 °C/min, then heated to 215 °C at 3 °C/min and hold for 10 min, heated to 230 °C at 3 °C/min and hold for 5 min. The inlet was operated in split mode (1:20) at a temperature of 250 °C with helium as the carrier gas at constant flow of 1.0 mL/min. The transfer line temperature was 215 °C, and the MS ion source was set to 230 °C. MS detection was carried out in electron impact (EI) ionization mode, scanning all masses from 45–400 amu. FAME components were identified based on mass spectral comparison with an external standard (Supelco 37 Component FAME Mix, Sigma-Aldrich, St. Louis, MO, USA) and previous studies [14−15]. The main fatty acid

composition was expressed as a weight percentage of the total fatty acids from three replicates.

Statistical analysis

The data analysis for morphological traits and oil characteristics were performed with SPSS® 24.0 (IBM®). The following parameters were evaluated: mean, minimum value, maximum value, standard deviation (SD) and coefficient of variation (CV%). One-way ANOVA was used in the comparison of all traits among subsp. of *sinensis*, subsp. of *mongolica* and hybrids. Pearson correlation coefficients were calculated to analyze the relationship between pairs of 8 agronomic traits. Principal component analysis (PCA) was used to determine relationships among the varieties. In addition, a canonical correspondence analysis (CCA) was applied to the data between morphological characteristics and oil traits in different tissues (seeds and pulp/peel).

DNA extraction and SSR analysis

Total genomic DNA was extracted from young leaves using the TaKaRa MiniBEST Plant Genomic DNA Extraction Kit (TaKaRa, Beijing, China) based on the manufacturer's protocol. Purity and quantity of extracted DNA were evaluated by gel electrophoresis and NanoDrop 2000 Spectrophotometer (Thermo Fisher Scientific, Waltham, MA, USA). Twenty polymorphic microsatellite loci (SSR) were evaluated, including previously published loci SB1-SB17 [17] and our newly developed loci SB18-SB20 using RNA-Seq (S2 Table). PCR amplification was performed in 20 μL

volumes containing 40 ng of DNA template, 1× PCR buffer, 1.5 mM $MgCL_2$, 0.15 mM of each dNTP (Takara, Dalian, China), 1.5 U of Taq polymerase (Takara, Dalian, China) and 0.5 μM of each primer. The PCR conditions included an initial denaturation at 94 °C for 2 min, 35 cycles of 30 s at 94 °C for denaturation, 30 s at 54–60 °C for annealing and 45 s at 72 °C for extension, with a final extension 7 min at 72 °C using a C1000 Touch™ Thermal Cycler (Bio-Rad, Berkeley, CA, USA). PCR products were electrophoresed on 8% nondenaturing polyacrylamide gels using a SE 600 Ruby Standard Dual Cooled Vertical Unit (GE Healthcare Life Sciences, Pittsburgh, PA, USA) and visualized by silver staining.

The microsatellites were scored as codominant markers for genetic diversity analysis. The number of alleles (Na), effective number of alleles (Ne), observed and expected heterozygosity (Ho and He), Shannon's information index (Is) and polymorphic information content (PIC) for each of the genic SSR markers were calculated using GenAlEx 6.5 [24–25] and PowerMarker version 3.25 [26] software packages. A genetic similarity matrix based on the proportion of shared alleles was generated, and a UPGMA tree was constructed using PowerMarker. The dendrogram was displayed using MEGA 6 software [27] to reveal genetic relationships between the 78 sea buckthorn varieties.

Results

Morphological characterization of berries and seeds

Descriptive statistics analysis of 8 agronomic fruit traits for the 78 sea buckthorn

varieties from different origins is shown in Table 2 and Table 3. Minimum, maximum, mean, standard deviation (SD), and coefficient of variation (CV%) were recorded. Relatively high CV values were observed in HBW, BLD, and HSW (> 20%). The highest coefficient of variation was observed in HBW (39.12%), which varied from 8.52 to 69.74 g. Analysis of variance (ANOVA, $p < 0.05$) showed that HBW of ssp. *mongolica* berries was 47.69 ± 11.03 g, which was much higher than ssp. *sinensis* berries (10.73 ± 1.54 g) and hybrids (31.44 ± 13.84 g). In hybrids, the HBW values were high in EZ4, Za56, Za1-2, Za05-6 and Za05-21(> 45 g), which were approximately the size of ssp. *mongolica* berries on average (S1 Table). BTD varied from 5.54 to 10.80 mm and BLD varied from 4.83 to 14.25 mm. In addition, BLD of berries from ssp. *mongolica* was higher than BTD, which was the opposite in berries of ssp. *sinensis*. According to BSI values, the berry shapes of the three groups were significantly different ($p = 0.000$): oblong berries of ssp. *mongolica* (1.35 ± 0.20), oblate for those of ssp. *sinensis* (0.90 ± 0.05) and circular for those of hybrids (1.08 ± 0.11). HSW varied from 0.61 to 2.19 g with an average of 1.45 g. Similar to HBW, there were significant differences for HSW among seeds from ssp. *mongolica*, ssp. *sinensis*, and hybrids ($p = 0.000$). SL varied from 2.00 to 3.49 mm and SW varied from 2.98 to 7.43 mm. ST varied from 1.54 to 2.73 mm with an average of 1.93 mm. Overall, the agronomic characters of seeds (HSW, SL, SW, and ST) showed relatively low coefficients of variation, ranging between 11.50–24.33%; however, the berries (HFW, FTD, FLD, and FSI) had high coefficients of variation.

Table 2. Descriptive statistics for morphological traits of berries and seeds among the sea buckthorn varieties studied.

Trait name	Abbrev. [a]	Min	Max	Mean	SD	CV(%)
Hundred berry weight (g)	HBW	8.52	69.74	40.68	15.92	39.12
Berry transverse diameter (mm)	BTD	5.54	10.80	7.85	1.20	15.25
Berry longitudinal diameter (mm)	BLD	4.83	14.25	9.76	2.23	22.87
Berry shape index	BSI	0.85	1.89	1.24	0.23	18.55
Hundred seed weight (g)	HSW	0.61	2.19	1.45	0.35	24.33
Seed length (mm)	SL	2.98	7.43	5.38	1.03	19.06
Seed width (mm)	SW	2.00	3.49	2.65	0.30	11.37
Seed thickness (mm)	ST	1.54	2.73	1.93	0.22	11.50

[a] Abbrev., Abbreviation

Table 3. Fruit traits of sea buckthorn berries of different origins[a].

Character	ssp. *mongolica*		ssp. *sinensis*		Hybrid	
	mean	SD	mean	SD	mean	SD
HBW (g)	47.69a	11.03	10.73c	1.54	31.44b	13.84
BTD (mm)	8.17a	0.99	5.84b	0.23	7.61a	1.24

BLD (mm)	10.90a	1.48	5.20c	0.19	8.15b	1.18
BSI (%)	1.35	0.20	0.90	0.05	1.08	0.11
HSW (g)	1.60a	0.28	0.79c	0.23	1.28b	0.25
SL (mm)	5.91a	0.68	3.31c	0.27	4.64b	0.56
SW (mm)	2.76a	0.27	2.18c	0.18	2.52b	0.22
ST (mm)	1.98a	0.18	1.67b	0.16	1.86a	0.26

[a] Values with different lower case letters (a–c) are significantly different at $p < 0.05$.

PCA was performed using fruit characteristics (Fig 2). The first two principal components explained 78.11% of the total morphological variance. The first principle component (PC) accounted for 41.74% of the variance. It was associated with BTD, HBW, ST, HSW, and SW in descending order. Therefore, these traits were important attributes for the classification of sea buckthorn varieties. The second PC accounted for 36.37%, which is correlated with BSI, SL, and BLD in descending order. The plot shows the distribution of 78 sea buckthorn varieties on PC1 and PC2 (Fig 2). The ssp. *mongol*ica varieties with bigger berries tended to cluster together, mainly positive on PC2. Six varieties of ssp. *sinensis* with the smallest berries were negative on both PC1 and PC2. The hybrids were largely distributed between the above two groups. Some hybrids (including ZCY1, ZCY10, ZCY12, XZC26, SCY2, and SCY5) were close to the varieties from ssp. *sinensis*.

Fig 2. Two-dimensional scatter plot for the first two principal components (PC1 and PC2) based on the agronomic fruit characteristics of 78 sea buckthorn varieties. Numbers associated with symbols are the variety codes as listed in Table 1. ▲ = ssp. *mongolica*; ● = ssp. *sinensis*; ◇ = hybrid.

Oil characterization in seeds and seedless parts

The oil characteristics of seeds and seedless parts (pulp and peel) among the 78 varieties of different origins are summarized in Tables 4 and Table 5. One special feature of sea buckthorn fruit was the high oil content in the pulp and peel (20.41%), in contrast to oil in seeds (8.82%). A higher coefficient of variation was observed in pulp/peel oil content (42.72%) and varied over a wide range, from 3.46 to 38.56%. The pulp/peel fraction of berries of ssp. *mongolica* had the highest oil content (24.68%) based on dry weight. The lowest pulp/peel oil content (7.10%) on average was found in the berries of ssp. *sinensis*. In hybrids, the berries of ZJ2 contained 27.22% pulp/peel oil, which slightly exceeded that of ssp. *mongolica* on average (S1 Table). Seed oil content varied from 3.88 to 12.75% with an average of 8.82%. The seeds of ssp. *mongolica* had the highest oil contents with an average of 9.46%, and those of the other two groups did not differ significantly.

Table 4. Oil characteristics of pulp/peel and seeds of 78 sea buckthorn varieties.

Character	Pulp/peel					Seed				
	Min	Max	Mean	SD	CV(%)	Min	Max	Mean	SD	CV(%)
oil content	3.46	38.56	20.41	8.72	42.72	3.88	12.75	8.82	1.86	21.08

16:0	24.52	53.08	36.26	4.83	13.32	3.84	11.77	6.55	1.39	21.16
16:1n7	17.93	57.75	35.12	7.64	21.76	tr[a]	tr[a]	tr[a]		
18:0	0.38	5.12	1.26	0.70	55.58	1.41	4.58	2.16	0.43	20.11
18:1n9	1.44	23.43	8.72	4.72	54.13	3.05	25.95	13.25	4.04	30.50
18:1n7	3.51	24.24	7.68	4.09	53.28	0.45	2.38	1.20	0.47	39.17
18:2n6	3.02	17.40	9.97	3.18	31.91	34.22	52.75	42.17	3.60	8.54
18:3n3	0.12	7.16	1.00	1.03	102.83	21.37	47.16	34.67	4.42	12.75

[a] tr, trace (< 0.5%).

Table 5. Oil content and fatty acid composition in seeds and the soft parts of sea buckthorn berries of different origins[a].

	Pulp/peel oil						Seed oil					
	ssp. *mongolica*		ssp. *sinensis*		Hybrid		ssp. *mongolica*		ssp. *sinensis*		Hybrid	
**Character**	**mean**	**SD**	**mean**	**SD**	**mean**	**SD**	**mean**	**SD**	**mean**	**SD**	**mean**	**SD**
oil content	24.68a	6.79	7.10c	3.28	13.34b	4.85	9.46a	1.56	6.70b	1.32	7.78b	1.84
16:0	37.68a	4.64	29.39b	3.71	34.62a	3.14	6.52	1.16	7.41	1.55	6.38	1.82
16:1n7	37.43a	7.09	23.65b	4.16	32.55a	5.84	tr[b]		tr[b]		tr[b]	
18:0	1.08b	0.69	1.73a	0.64	1.59ab	0.57	2.13	0.29	2.19	0.44	2.23	0.69
18:1n9	7.56b	3.97	16.67a	6.84	9.33b	3.40	12.62b	3.75	16.37a	3.77	13.96ab	4.46
18:1n7	6.01c	1.79	16.68a	6.20	9.32b	3.63	1.07b	0.37	1.80a	0.39	1.37b	0.55
18:2n6	9.55ab	2.76	8.34b	5.54	11.53a	2.92	42.10	3.08	40.44	4.06	42.87	4.62
18:3n3	0.69b	0.41	3.54a	2.09	1.07b	0.64	35.56a	4.13	31.78b	2.91	33.20ab	4.89

MUFA	51.00b	5.38	57.00a	9.46	51.20b	3.52	13.69b	3.93	18.18a	4.09	15.33ab	4.90
PUFA	10.24	2.98	11.89	7.54	12.60	3.37	77.66a	4.31	72.22b	5.54	76.06ab	6.23

[a] Values with different lowercase letters (a–c) are significantly different at $p < 0.05$.

[b] tr, trace (< 0.5%).

For sea buckthorn, the FA composition in seed oil differed significantly from that in pulp/peel oil. The proportions of linoleic (18:2n6), *α*-linolenic (18:3n3), oleic (18:1n9), palmitic (16:0), stearic (18:0) and vaccenic (18:1n7) acids were found from high to low in seed oil of most varieties (Table 4). Linoleic acid varied from 34.22 to 52.75% with an average of 42.17%. The proportion of *α*-linolenic acid varied from 21.37 to 47.16% with an average of 34.67%. High CV values were observed in oleic (30.50%) and vaccenic (39.17%) acids. Furthermore, the level of palmitoleic acid (16:1n7, < 0.5%) was extremely low in seed oil. The FA composition of sea buckthorn seeds were similar among berries of different origins. Small variations were found in the proportion of linoleic acid in seed oil (40.44 – 42.87%). Its proportion in hybrids were slightly higher than in ssp. *mongolica* (42.87% vs 42.10%), and had the highest value of the samples from three different origins. *α*-Linolenic acid showed a little variation with a bigger proportion in ssp. *mongolica* than in ssp. *sinensis* (35.56% vs 31.78%). A higher proportion of palmitic (7.41% vs 6.38%) and oleic (16.37% vs 13.96%) acids and a lower proportion of stearic acid (2.19% vs 2.23%) were discovered between the varieties of ssp. *sinensis* and hybrids. The polyunsaturated fatty acids (PUFA) ratio in hybrids (76.06%) was slightly lower than it was in ssp. *mongolica* (77.66%) and higher than it was in ssp. *sinensis* (72.22%). Some hybrids (including ZJ1, Za1-2, Za13-25, Za05-6, LFZ, and ZCY12) contained a high proportion of PUFA (> 80%) in seed oil, which was more than the average level of ssp. *mongolica* varieties (S1 Table).

In pulp/peel oil, the dominate FAs were palmitoleic, palmitic, linoleic, oleic, and vaccenic acids (Table 4). High deviations were identified in the proportion of palmitoleic (17.93-57.75%), oleic (1.44-23.43%) and vaccenic (3.51-24.24%) acids. The special feature of pulp/peel oil is high proportions (> 35%) of palmitoleic and palmitic acids. Compared to ssp. *sinensis*, ssp. *mongolica* contained a higher proportion of palmitoleic and palmitic acids in the berry pulp/peel ($p < 0.05$) (Table 5). In particular, the proportions of oleic and vaccenic acids were highest in ssp. *sinensis*, much higher than the other two origins. The relative levels of α-linolenic and stearic acids in pulp/peel of ssp. *sinensis* were higher than ssp. *mongolica* ($p < 0.05$) (Table 5). For hybrids, the proportions of most fatty acids were between ssp. *mongolica* and ssp. *sinensis* varieties, except for linoleic acid. Similar to the results in seed oils, the hybrids had the highest proportions of linoleic acid (11.53%) and PUFA (12.60%). The monounsaturated fatty acids (MUFA) ratio in pulp/peel oil of ssp. *sinensis* (57.00%) was highest and that of ssp. *mongolica* (51.00%) was almost equal to hybrids (51.20%). In hybrids, the pulp/peel oil of SCY2 contained 39.16% palmitoleic acid, and the content of MUFA was 60.77%, which was higher than it was in ssp. *sinensis* (S1 Table).

Correlations among the agronomic traits and oil characters

Canonical analyses allow direct comparisons of two data matrices. All sea buckthorn varieties were represented in a two-dimensional space using CCA between phenotypic traits and oil characteristics (Fig 3). For berries of different origins, phenotypic characters (BLD, HBW, BSI, and BTD) of berries and oil traits in pulp/peel showed

close correlation (*r* = 0.8725, *p* = 0.0000). Based on CCA, varieties of ssp. *mongolica* were clustered on the upper side (mainly positive on D1 and D2), those of ssp. *sinensis* on the other, and the hybrids in the middle in Fig 3A. The positioning of samples in the first dimension was mostly related to differences in their berry characteristics that were primarily provided by a marker of BLD. The second dimension indicated differences in oil contents and FA compositions of pulp/peel oil among sea buckthorn varieties. Differences between pulp/peel oil traits were primarily related to percentages of oil content, 16:0 and 16:1n7, which were highest in ssp. *mongolica*, followed by hybrids, and lowest in ssp. *sinensis*. For seeds of different origins, phenotypic characters (SL, SW, ST, and HSW) and seed oil traits were correlated (*r* = 0.7482, *p* = 0.0000). The positioning of samples was staggered (Fig 3B), which reflected that all seed samples from different origins had relatively little variation among phenotypic traits and oil characteristics. These results verified the previous analysis (Table 3 and Table 5).

Fig 3. Canonical correspondence analysis of phenotypic traits (A. berry; B. seed) and oil characteristics (A. pulp/peel oil; B. seed oil) of sea buckthorn germplasms. D1, Dimension 1; D2, Dimension 2. ▲ = ssp. *mongolica*; ● = ssp. *sinensis*; ◇ = hybrid.

SSR diversity

Twenty pairs of RNA-Seq SSR primers with good amplification and band stability were used in 78 varieties of sea buckthorn. All the primers amplified 59 bands, of which 51 were polymorphic, accounting for 86.44%. The number of amplified bands per locus

ranged from 2 to 5, averaging 2.9500, and the number of effective alleles (Ne) ranged from 1.0392 to 3.1049, averaging 1.6279 (Table 6). SB2, SB3, SB5, SB6, SB8, SB13 and SB16 were informative SSR loci, each revealing more than four effective alleles distributed among all of the varieties. Compared with the observed allele number (Na), the number of effective alleles and their average values were lower, which was caused by the uneven distribution of gene frequencies in SSR loci. In genetic diversity analysis, observed heterozygosity (Ho) ranged from 0.0385 to 0.7051, with an average of 0.2519; expected heterozygosity (He) ranged from 0.0377 to 0.6779, with an average of 0.3140, and the Shannon index (Is) ranged from 0.0950 to 1.2152, with an average of 0.5449. The value of polymorphism information content (PIC), regarded as discriminating power, varied from 0.0370 to 0.6174, with an average of 0.2725. Loci SB06 (PIC = 0.6174) and SB08 (PIC = 0.5820) showed higher effectiveness because of their high informativity, which could be used to construct the fingerprint map of sea buckthorn germplasm. The characteristics of these 20 loci in genetic diversity analysis of sea buckthorn germplasm are shown in Table 6.

Table 6. Characterization of 20 polymorphic SSR markers in the 78 sea buckthorn varieties.

Loci code	Na	Ne	Ho	He	PIC	Is
SB1	3	1.2745	0.2436	0.2154	0.2025	0.3956
SB2	4	1.1382	0.1282	0.1214	0.1166	0.2791
SB3	4	2.2372	0.4615	0.5530	0.4627	0.9090

SB4	2	1.5006	0.2692	0.3336	0.2779	0.5160
SB5	4	2.1129	0.3333	0.5267	0.4735	0.9288
SB6	4	3.1049	0.7051	0.6779	0.6174	1.2152
SB7	2	1.0799	0.0769	0.0740	0.0712	0.1630
SB8	5	2.8490	0.3846	0.6490	0.5820	1.1890
SB9	2	1.1509	0.1410	0.1311	0.1225	0.2550
SB10	3	1.5350	0.2949	0.3485	0.3114	0.6253
SB11	2	1.9287	0.1667	0.4815	0.3656	0.6745
SB12	3	1.2430	0.2179	0.1955	0.1753	0.3687
SB13	4	2.1644	0.4231	0.5380	0.4392	0.8687
SB14	2	1.9987	0.3077	0.4997	0.3750	0.6928
SB15	2	1.0662	0.0641	0.0620	0.0601	0.1418
SB16	4	1.4567	0.1923	0.3135	0.2956	0.6427
SB17	2	1.4175	0.3590	0.2945	0.2512	0.4706
SB18	2	1.0392	0.0385	0.0377	0.0370	0.0950
SB19	3	1.0804	0.0641	0.0744	0.0724	0.1804
SB20	2	1.1803	0.1667	0.1528	0.1411	0.2868

Na, observed number of alleles; Ne, effective number of alleles; Ho, observed heterozygosity; He,

expected heterozygosity; PIC, polymorphism information content; Is, Shannon's information index.

Genetic relationships among sea buckthorn germplasm

The sea buckthorn germplasm in this study originated from ssp. *mongolica* (52 varieties), ssp. *sinensis* (6 varieties) and hybrids (20 varieties). Using 20 polymorphic SSR markers, the UPGMA dendrogram based on the proportion of shared alleles was constructed to assess the genetic relationships between the 78 varieties (Fig 4). The results showed that all the varieties could be divided into two groups (I and II). The varieties of ssp. *sinensis* (JD, ZGSJ, MHC, ZGSJ^{wild}, JPDH and ZXY) and 7 hybrids (ZCY1, ZCY10, ZCY12, SCY2, SCY5, SCY30, and XZC26) from Dongsheng were clustered into group I. Among them, 6 cultivars of ssp. *sinensis* had closer relationships than the hybrids, despite great geographic differences. The second group was divided into 3 subgroups, namely, IIIa, IIIb, and IIIc. The other 13 hybrids (ZJ1, ZJ2, ZJ3, Za13-19, Za13-25, Za05-6, Za05-20, Za05-21, Za1-2, Za4, EZ4, Za56, and LFZ) from Fuxin and Suiling were clustered into IIa. Subgroup IIb and IIc contained 52 varieties of ssp. *mongolica* (introduced from Russia and Mongolia) without exception. In subgroup IIb, QYSK, LS1, WCF, FX and MZ14 were closely associated, and HS-9, HS12, HS14, HS18, HS20, HS22, WLGM and KTN clustered together. In subgroup IIc, ZYH, SD, NR, FL, JYH, YLSD12, SJ3, XE2 and XE3 were closely related, and the other 30 varieties from Suiling clustered together. Overall, the relationship between ssp. *mongolica* and ssp. *sinensis* was relatively distant, and that of hybrids was between the two. Some hybrids are close to ssp. *sinensis* and others to ssp. *mongolica*.

Fig. 4. UPGMA dendrogram of sea buckthorn germplasm based on SSR data (sample abbreviations described in Table 1). ▲ = ssp. *mongolica*; ● = ssp. *sinensis*; ◇ = hybrid.

Discussion

Morphological characteristics, biochemical traits, and microsatellite markers have been used for germplasm identification and genetic diversity analysis in many horticultural plants [28–29]. The diversity at morphological, biochemical, and molecular levels of 78 sea buckthorn varieties, composed of 52 from ssp. *mongolica*, 6 from ssp. *sinensis*, and 20 hybrids, were investigated.

The morphological characterization of plant materials with desired traits is an essential step for the effective use of germplasm [30]. Here, 8 important agronomic traits were measured among 78 sea buckthorn varieties, and a considerable amount of variation in morphological traits was found. The berry sizes of berries from three origins were significantly different according to the HBW value ($p = 0.000$). Compared to ssp. *sinensis* berries, ssp. *mongolica* berries were much bigger on average. Hybrids between ssp. *mongolica* and ssp. *sinensis* were intermediate. In PC analysis, we plotted 2D plots with PC1 and PC2 scores of phenotypes (Fig 2). PC1 was mainly related with BTD and HBW, which explained the largest portion of the variance in varieties. The distribution of 78 varieties on PC1 and PC2 was consistent with their agronomic characters (Fig 2). These results estimating morphological traits are valuable tools for identifying variation among plant germplasms [28].

For biochemical traits, oil content and FA composition in seeds and seedless parts were selected for their important roles in human health. The oil of sea buckthorn seems to be a good source of unsaturated fatty acids. The seed oil is rich in PUFA, including linoleic and α-linolenic acids. The proportion of PUFA did not differ significantly among berries from three origins, despite the differences in some morphological characteristics and in growth conditions. These results were consistent with the previous studies [14]. The results of the present study and previous investigations also suggested that the berries of ssp. *mongolica* were a good source of palmitic and palmitoleic acids in pulp/peel oil and those of ssp. *sinensis* were a good source of oleic acid, both in seeds and pulp/peel [31]. Although carefully selected for intersubspecies crosses, some hybrids displayed elite oil traits. For example, the proportion of MUFA in pulp/peel of SCY2 and of PUFA in seeds of 6 varieties (including ZJ1, Za1-2, Za13-25, Za05-6, LFZ, and ZCY12) exceeded the average level of varieties of ssp. *mongolica*, the subspecies of one of their parents. These results demonstrate the effectiveness of traditional cross breeding in the improvement of native varieties (ssp. *sinensis*), even though it is time-consuming and has low efficiency.

Previous studies found that berry size is a useful indicator of Vc, sugars and acids in comparisons of populations [22, 32]. The nutrients in the seedless fraction were more concentrated in the small berries of ssp. *sinensis* than in the large berries of ssp. *mongolica* [31]. In the present study, we analyzed the correlation between agronomic characteristics and oil traits at different levels (seed and pulp/peel) by CCA. The results showed phenotypic characteristics (BLD, HBW, BSI, and BTD) of berries and oil traits

in pulp/peel were positively correlated (r = 0.8725, p = 0.0000). BLD, as a promising marker, provided the primary difference in CCA. Our results illustrated that berry size had different correlations with various biochemical characteristics in sea buckthorn.

Variation of phenotypic traits among germplasms may be attributed to differences in genetic backgrounds, geographical location, climate, harvest period and berry maturity, while molecular markers are independent of environmental condition and growth stage [33]. Twenty polymorphic SSR markers were used to identify 78 sea buckthorn varieties of different origins. The selected 20 SSR markers detected 2–5 alleles, and their PIC values ranged from 0.0370 to 0.6174 and had an average of 0.2725. The PIC mean value was significantly lower than that of RAPD, ISSR and SRAP markers previously reported [18–19, 34], suggesting that the gene sequences of these SSR markers were conservative in sea buckthorn germplasm.

Based on UPGMA, the 78 varieties were classified into two groups. There is a large genetic distance between varieties of ssp. *sinensis* and ssp. *mongolica*. Seven varieties (ZCY1, ZCY10, ZCY12, XZC26, SCY2, SCY5, and SCY30) of hybrids were closely related to ssp. *sinensis* varieties and clustered into one group. Coincidentally, these hybrids were also closely related o ssp. *sinensis* varieties on the PCA plot based on 8 agronomic characters. This result illustrated that the diversity of morphological characters could reflect genetic diversity and be used as markers in agronomy. However, the other 13 hybrids were close related to ssp. *mongolica* varieties. The separation among hybrids may be because their parents belonged to subspecies *mongolica* and *sinensis*. Ruan et al. [18] assessed 14 Chinese, Russian and Mongolian sea buckthorn

varieties using RAPD markers and obtained similar results.

With the continuous development of high-throughput sequencing technology, transcriptome databases have become a powerful resource for SSR mining. More and more RNA-Seq SSRs have been developed and applied to the study of species genetic diversity and population genetic structure [35–36]. The SSRs obtained by transcriptomes are associated with many important quantitative traits [37].

Conclusion

In the present study, 8 phenotypic characteristics, oil traits in seeds and seedless parts, and 20 SSR markers successfully distinguished all 78 sea buckthorn varieties. In PC analysis, BTD and HBW in the first PC were the most important characteristics for distinguishing the varieties. The agronomic traits of berries were closely correlated with the oil content and FA composition in pulp/peel by CCA. This information will be valuable for cultivar identification and genotypic diversity analysis in *Hippophae rhamnoides*.

Supporting information

S1 Fig. 78 berry samples used in this study. Numbers are the variety codes as listed in Table 1.

(TIF)

S2 Fig. Total ion flow chromatography of 37 FAMEs Mix (A) and FAMEs in pulp/peel oil in MHC (B).

(TIF)

S1 Table. The morphological characteristics and oil traits of pulp and seeds of 78 sea buckthorn varieties studied.

(XLSX)

S2 Table. Primer sequences, annealing temperature, and estimated allelic size of 20 SSR markers.

S3 Table. Allele combinations obtained at the 20 microsatellite loci in 78 sea buckthorn varieties.

(TXT)

Acknowledgements

The authors are grateful to Hai Guo (Jiuchenggong Breeding Base of Sea Buckthorn) and Jun Zhang (Institute of Selection and Breeding of *Hippophae*) for the collection of plant materials.

References

1. N, Swenson U, Nybom H. Phylogeny of *Hippophae* (Elaeagnaceae) inferred from parsimony analysis of chloroplast DNA and morphology. Syst Bot. 2002; 27:41–54.
2. Swenson U andBartish IV. Taxonomic synopsis of *Hippophae* (Elaeagnaceae). Nord J Bot. 2002; 22:369–374.
3. Kallio H, Lassila M, Järvenpää E, Haraldsson GG, Jonsdottir S, Yang BR. Inositols and methylinositols in sea buckthorn (*Hippophaë rhamnoides*) berries. J Chromatogr B. 2009;

877:1426−1432.

4. Chen C, Xu XM, Chen Y, Yu MY, Wen FY, Zhang H. Identification, quantification and antioxidant activity of acylated flavonol glycosides from sea buckthorn (*Hippophae rhamnoides* ssp. *sinensis*). Food Chem. 2013; 141, 1573−1579.

5. Pop MR, Weesepoel Y, Socaciu C, Pintea A, Vincken JP, Gruppen H. Carotenoid composition of berries and leaves from six Romanian sea buckthorn (*Hippophae rhamnoides* L.) varieties. Food Chem. 2014; 147:1−9.

6. Teleszko M, Wojdyło A, Rudzińska M, Oszmiański J, Golis T. Analysis of Lipophilic and Hydrophilic Bioactive Compounds Content in Sea Buckthorn (*Hippophaë rhamnoides* L.) Berries. J Agric Food Chem. 2015; 63:4120−4129.

7. Raffo A, Paoletti F, Antonelli M. Changes in sugar, organic acid, flavonol and carotenoid composition during ripening of berries of three seabuckthorn (*Hippophae rhamnoides* L.) cultivars. Eur Food Res Technol. 2004; 219:360−368.

8. Bal ML, Meda V, Naik NS, Satya S. Sea buckthorn: A potential source of valuable nutrients for nutraceuticals and cosmoceuticals. Food Res Int. 2011; 44:1718−1727.

9. Suryakumar G, Gupta A. Medicinal and therapeutic potential of Sea buckthorn (*Hippophae rhamnoides* L.). J Ethnopharmacol. 2011; 138:268−278.

10. Patel C A, Divakar K, Santani D, Solanki H K, Thakkar JH. Remedial prospective of *Hippophaë rhamnoides* Linn. (sea buckthorn). ISRN Pharmacol. 2012; 2012:1−6.

11. Suomela JP, Ahotupa M, Yang B, Vasankari T, Kallio H. Absorption of flavonols derived from sea buckthorn (*Hippophae rhamnoides* L.) and their effect on emerging risk factors for cardiovascular disease in humans. J Agric Food Chem. 2006; 54:7364−7369.

12. Grey C, Widén C, Adlercreutz P, Rumpunen K, Duan RD. Antiproliferative effects of sea buckthorn (*Hippophae rhamnoides* L.) extracts on human colon and liver cancer cell lines. Food Chem.2010; 120: 1004−1010.

13. Xu YJ, Kaur M, Dhillon SR, Tappia SP, Dhalla SN. Health benefits of sea buckthorn for the prevention of cardiovascular diseases. J Func Foods. 2011; 3:2−12.

14. Yang B and Kallio H. Composition and Physiological Effects of Sea Buckthorn Lipids, Trends Food Sci Technol. 2002; 13:160–167.

15. Yang B and Kallio H. Fatty acid composition of lipids in sea buckthorn (*Hippophaë rhamnoides* L.) berries of different origins. J. Agric. Food Chem. 2001; 49:1939−1947.

16. Ruan CJ, Teixeira da Silva JA, Jin H, Li H, Li DQ. Research and biotechnology in sea buckthorn (*Hippophae* spp.). Medicinal and Aromatic Plant Science and Biotechnology. 2007; 1: 47−60.

17. Li H, Ruan CJ, Wang L, Ding J, Tian XJ. Development of RNA-Seq SSR markers and application to genetic relationship analysis among sea buckthorn germplasm. J Amer Soc Hort Sci. 2017; 142(3):200-208.

18. Ruan CJ. Genetic relationships among some sea buckthorn cultivars from China, Russia and Mongolia using RAPD markers. Sci Hort. 2004; 101:417–426.

19. Li H, Ruan CJ, Teixeira da Silva J, Liu BQ. Associations of SRAP markers with dried-shrink disease resistance in a germplasm collection of sea buckthorn (*Hippophae* L.). Genome. 2010; 53:447–457.

20. Kalia RK, Rai MK, Kalia S, Singh R, Dhawan AK. Microsatellite markers: An overview of the recent progress in plants. Euphytica. 2011; 177:309–334.

21. Jain A, Chaudhary S, Sharma PC. Mining of microsatellites using next generation sequencing of seabuckthorn (*Hippophae rhamnoides* L.) transcriptome. Physiol. Mol. Biol. Plants. 2014; 20:115–

123.

22. Tang X and Tigerstedt PMA. Variation of physical and chemical characters within an elite sea buckthorn (*Hippophae rhamnoides* L.) breeding population. Sci. Hort. 2001; 88(3):203–214.

23. Christie WW. A simple procedure for rapid transmethylation of glycerolipids and cholesteryl esters. J Lipid Res. 1982; 23:1072-1075.

24. Peakall R and Smouse PE. GENALEX 6: Genetic analysis in Excel. Population genetic software for teaching and research. Mol Ecol Notes. 2006; 6:288–295.

25. Peakall R and Smouse PE. GenAlEx 6.5: Genetic analysis in Excel. Population genetic software for teaching and research – An update. Bioinformatics. 2012; 28:2537–2539.

26. Liu K and Muse SV. PowerMarker: An integrated analysis environment for genetic marker analysis. Bioinformatics. 2005; 21:2128–2129.

27. Tamura SK, Peterson GD, Filipski A, Kumar S. MEGA6: Molecular evolutionary genetics analysis version 6.0. Mol Biol Evol. 2013; 30:2725–2729.

28. Lee ON and Park HY. Assessment of genetic diversity in cultivated radishes (*Raphanus sativus*) by agronomic traits and SSR markers. Sci Hort. 2017; 223:19-30.

29. Goodarzi S, Khadivi A, Abbasifar A, Akramian M. Phenotypic, pomological and chemical variations of the seedless barberry (*Berberis vulgaris* L. var. asperma). Sci Hort. 2018; 238:38–50.

30. Santos RC, Pires JL, Correa RX. Morphological characterization of leaf, flower, fruit and seed traits among Brazilian *Theobroma* L. species. Genet. Resour. Crop Evol. 2012; 59:327–345.

31. Kallio H, Yang B, Peippo P, Tahvonen R, Pan R. Triacylglycerols, glycerophospholipids, tocopherols and tocotrienols in sea buckthorn *Hippophae rhamnoides* L. ssp. *sinensis* and ssp. *mongolica* berries and seeds. J. Agric. Food Chem. 2002; 50:3004–3009.

32. Kallio H, Yang B, Peippo P. Effects of Different Origins and Harvesting Time on Vitamin C, Tocopherols, and Tocotrienols in Sea Buckthorn (*Hippophae1 rhamnoides*) Berries. J. Agric. Food Chem. 2002; 50:6136−6142.

33. Ali M, Rajewski J, Baenziger P, Gill K, Eskridge KM, Dweikat I. Assessment of genetic diversity and relationship among a collection of US sweet sorghum germplasm by SSR markers. Mol. Breed. 2008; 21:497–509.

34. Li, H., C.J. Ruan, and J.A. Teixeira da Silva. 2009. Identification and genetic relationship based on ISSR analysis in a germplasm collection of sea buckthorn (*Hippophae* L.) from China and other countries. Sci. Hort. 123:263–271.

35. Liu YL, Zhang PF, Song ML, Hou JL, Qing M, Wang WQ, Liu CS. Transcriptome analysis and development of SSR molecular markers in *Glycyrrhiza uralensis* Fisch. PLoS One. 2015; 10:e0143017. doi:10.1371/journal.pone.0143017

36. Zhang LW, Li YR, Tao AF, Fang PP, Qi JM. Development and characterization of 1,906 EST-SSR markers from unigenes in jute (*Corchorus* spp.), PLoS One. 2015; 10(10):e0140861. doi:10.1371/journal.pone.0140861

37. Ramchiary N, Nguyen VD, Li X, Hong CP, Dhandapani V, Choi SR, et al. Genic microsatellite markers in *Brassica rapa*: Development, characterization, mapping, and their utility in other cultivated and wild *Brassica* relatives. DNA Res. 2011; 18:305-320.

Figure

Click here to download Figure Fig_1.tif.tif

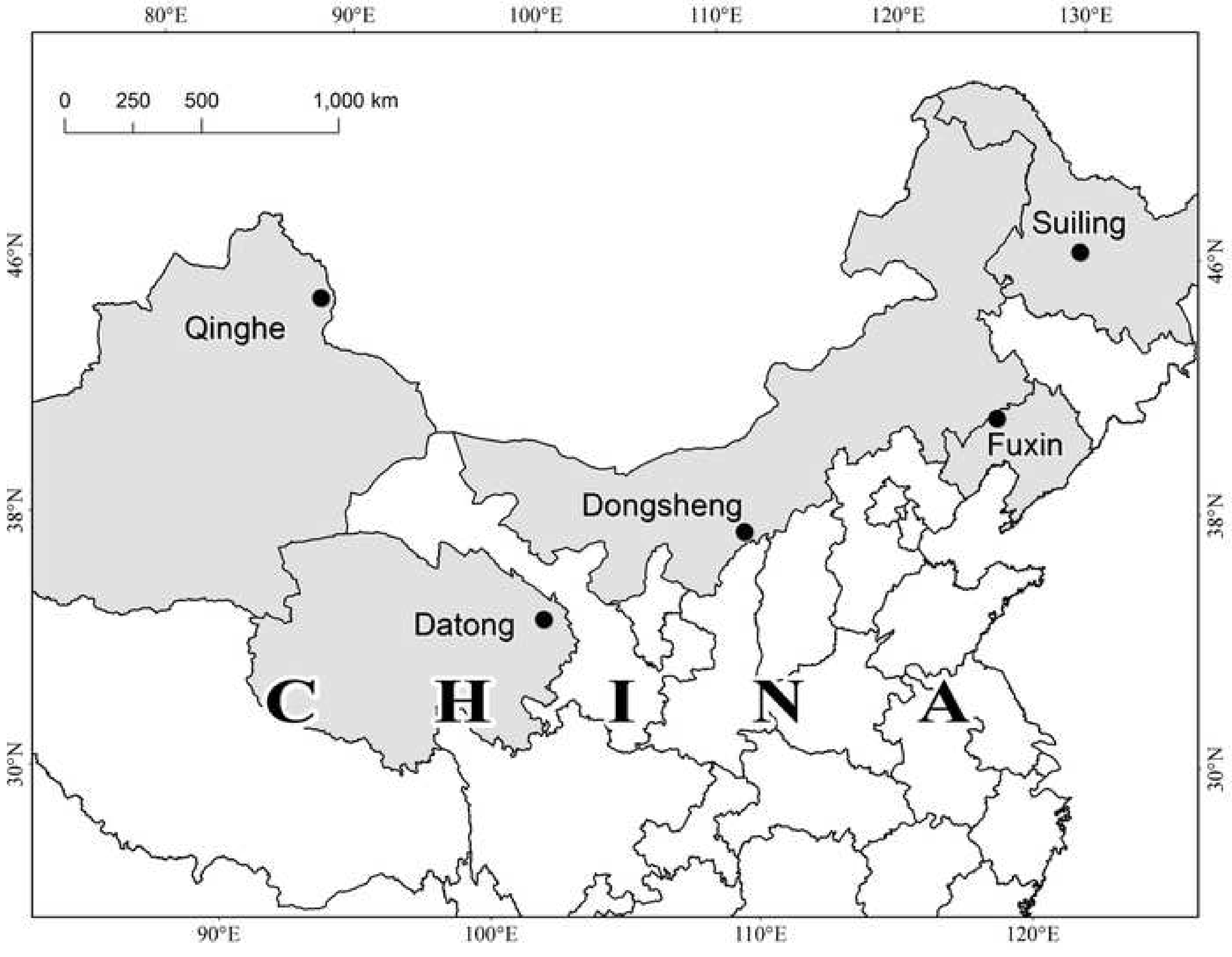

Figure

Click here to download Figure Fig_2.tif.tif

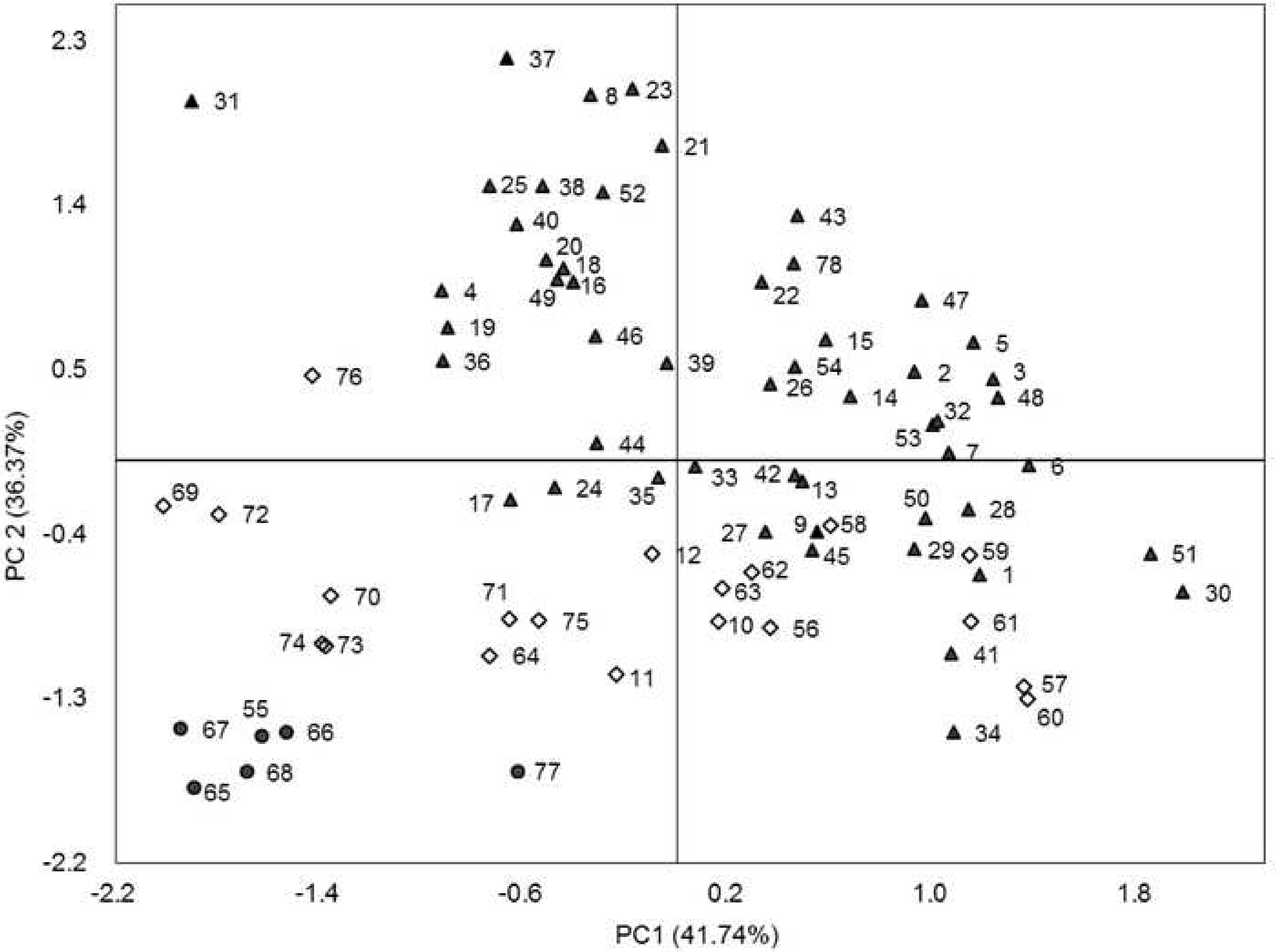

 Click here to download Figure Fig_3.tif.tif

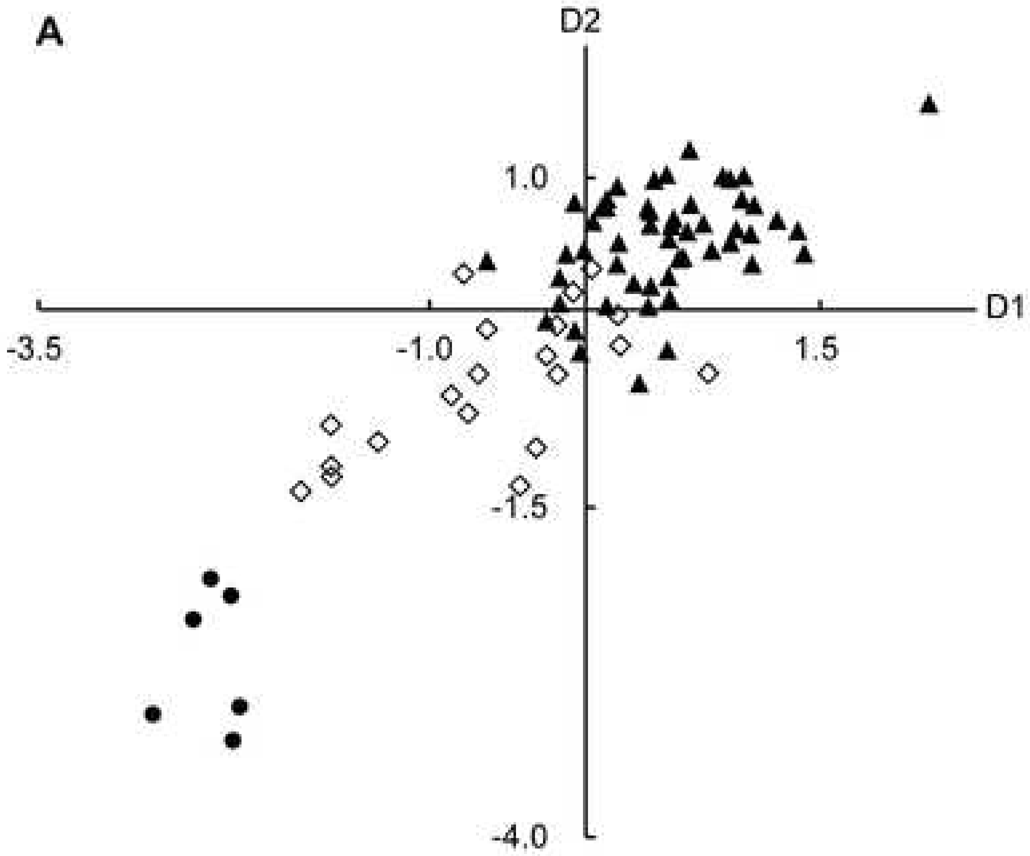

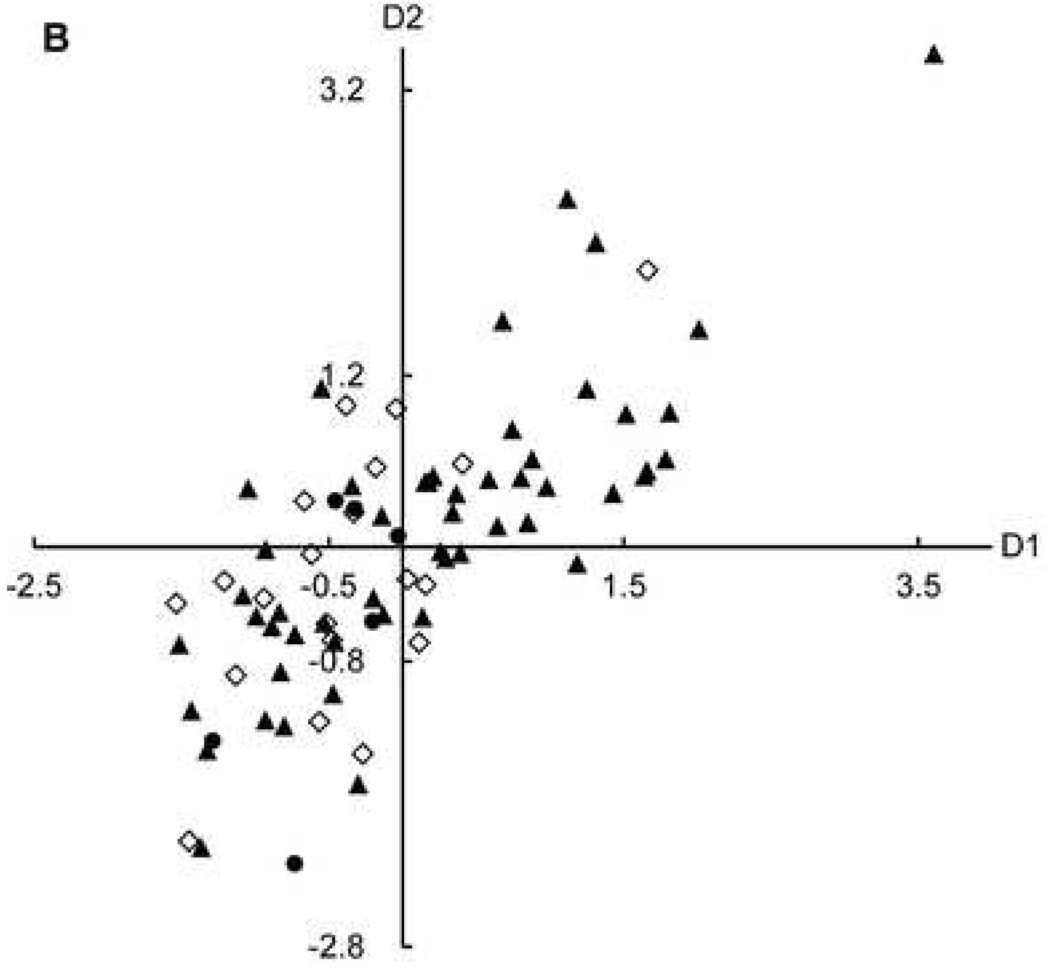

Figure

Click here to download Figure Fig_4.tif.tif

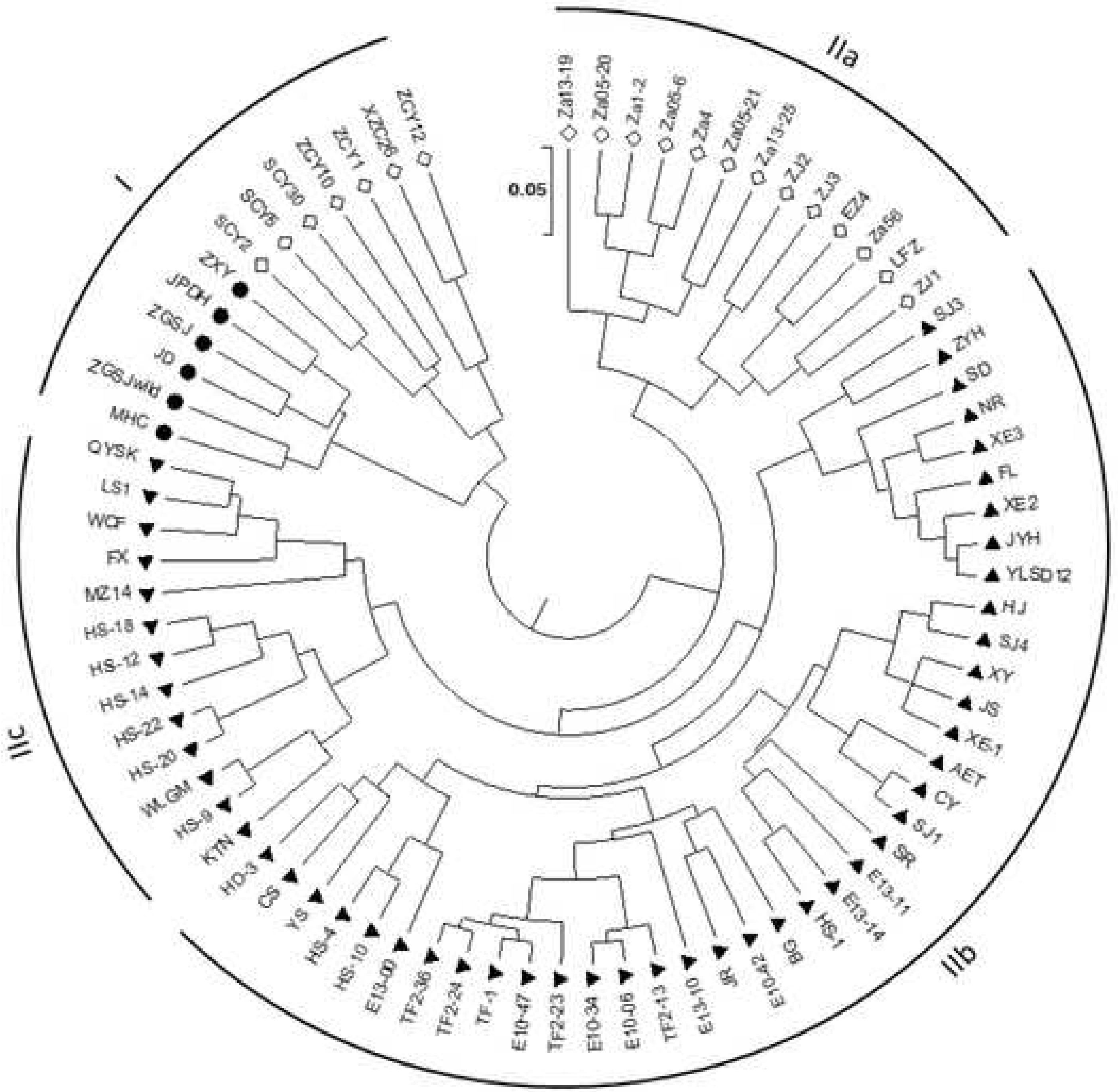

Click here to access/download
Supporting Information
S1_Fig.tif.tif

Click here to access/download
Supporting Information
S1_Table.xlsx.xlsx

Click here to access/download
Supporting Information
S2_Fig.tif.tif

Click here to access/download
Supporting Information
S2_Table.docx.docx

Click here to access/download
Supporting Information
S3_Table.txt.txt
