# Supplementary material for: Diversity in sea buckthorn (Hippophae rhamnoides L.) accessions with different origins based on morphological characteristics, oil traits, and microsatellite markers
Source: PLoS One. 2020 Mar 13;15(3):e0230356. doi: 10.1371/journal.pone.0230356 (PMC7069629; doi:10.1371/journal.pone.0230356)
Supplement: Attachment — Submitted filename: PONE-D-19-17567_R1-comments.pdf [file pone.0230356.s013.pdf]

PLOS ONE

Diversity in sea buckthorn (Hippophae rhamnoides L.) accessions with different origins based on morphological characteristics, oil traits, and microsatellite markers

--Manuscript Draft--

**Manuscript Number:**	PONE-D-19-17567R1
**Article Type:**	Research Article
**Full Title:**	Diversity in sea buckthorn (Hippophae rhamnoides L.) accessions with different origins based on morphological characteristics, oil traits, and microsatellite markers
**Short Title:**	Diversity in sea buckthorn accessions based on morphological characteristics, oil traits, and SSR markers
**Corresponding Author:**	Chengjiang Ruan<br>Dalian Medical University<br>Dalian, CHINA
**Keywords:**	diversity analysis; morphological characteristics; SSR markers; biochemical traits; fatty acid composition
**Abstract:**	Sea buckthorn (*Hippophae rhamnoides*) is an ecologically and economically important species. Here, we assessed the diversity of 78 accessions cultivated in northern China using 8 agronomic characteristics, oil traits (including oil content and fatty acid composition) in seeds and pulp, and SSR markers at 23 loci. The 78 accessions included 52 from ssp. *mongolica* , 6 from ssp. *sinensis* , and 20 hybrids. To assess the phenotypic diversity of these accessions, 8 agronomic fruit traits were recorded and analyzed using principal component analysis (PCA). The first two PCs accounted for approximately 78% of the variation among accessions. The oil contents were higher in pulp (3.46-38.56%) than in seeds (3.88-8.82%), especially in ssp. *mongolica* accessions. The polyunsaturated fatty acids (PUFA) ratio was slightly lower in seed oil of hybrids (76.06%) than in ssp. *mongolica* (77.66%) and higher than in ssp. *sinensis* (72.22%). The monounsaturated fatty acids (MUFA) ratio of pulp oil of ssp. *sinensis* (57.00%) was highest, and that of ssp. *mongolica* (51.00%) was approximately equal to the ratio in the hybrids (51.20%). Using canonical correspondence analysis (CCA), we examined the correlation between agronomic traits and oil characteristics in pulp and seeds, respectively. Oil traits in pulp from different origins were correlated with morphological groupings (r = 0.8725, p = 0.0000). To assess the genotypic diversity, 23 SSR markers (including 17 loci previously reported) were used among the 78 accessions with 69 polymorphic amplified fragments obtained and an average PIC value of 0.2845. All accessions were classified into two groups based on the UPGMA method. The accessions of ssp. *sinensis* and ssp. *mongolica* were genetically distant. The hybrid accessions were close to ssp. *mongolica* accessions. The 8 agronomic traits, oil characters in seed and pulp oils, and 23 SSR markers successfully distinguished the 78 accessions. These results will be valuable for cultivar identification and genetic diversity analysis in cultivated sea buckthorn.
**Order of Authors:**	He Li
	Chengjiang Ruan
	Jian Ding
	Jingbin Li
	Li Wang
	Xingjun Tian
**Response to Reviewers:**	Response to Reviewer' Comments letter to PLOS ONE<br>The authors thank the additional editor and two reviewers for their careful reading, comments and suggestion. We revised our manuscript in the best way as we could. Revised portions are marked in red in the revised manuscript. For the individual comments see our reply below.

Powered by Editorial Manager® and ProduXion Manager® from Aries Systems Corporation

Additional Editor Comments:

1) The use of term varieties, cultivars, subspecies and hybrids have been without much explanation. For example, what is the basis of assigning hybrid status to a particular cultivar? More clarity is required in explanation of the material. How these varieties were assigned varietal status?

Response: The Reviewer 1 gave the definition of ‘variety’ that "a variety must be recognizable by its characteristics, recognizably different from any other variety and remain unchanged through the process of propagation". The ‘cultivar’ refers to a variety of a plant developed from a natural species and maintained under cultivation. The authors accepted the reviewers’ advice that the term ‘accessions’ would be appropriate according to the plant materials in present study.
The hybrid accessions in this study generated by hybridization experiment in control between ssp. mongolica and ssp. sinensis at specialized experimental fields and selected for their desirable traits. After a complex process of identification of experts, some hybrids may became a new ‘cultivar’.

2) Generation of morphological dataset is also not mentioned clearly. You have 76 varieties growing at three locations. You need to provide environmental parameters for each location. Are all 76 growing at each location? If all the varieties are not growing at same location, many of morphological traits will be influenced by environmental factors. Did you do any multilocation trials to see the influence of environment on these traits? Did you try to collect data during different years and see if the data is consistent or showing variation. A statistical analysis of such data only will generate confidence in morphological data. Even a multilocation trial of a subset will provide information on reliability of data. Please include such data.

Response: The environmental parameters for each location were provided in the S2 Table of revised manuscript. All the accessions are not growing at same location. However, they could adapt to the environment of their cultivated lands well. We had performed some multi-location trials to see the influence of environment on berry characteristics before that was supplemented in the results of revised manuscript (S4 Table).

3) The sequencing data has been published earlier and 17 of SSR are coming from that data. Only 3 new markers have been used in the present study. This undermines the amount of data presented in this MS. You have to clearly mention these facts in the MS and the abstract. In my opinion more data needs to be generated. I suggest another 25-30 SSRs should be used for analysing the diversity.

Response: We have mentioned it in the MM and the abstract of the revised manuscript. We screened 3 new SSR loci (SB21-23) with polymorphism from 20 SSR primer pairs during the revision of the manuscript. These information has been supplemented in revised manuscript. It is difficult to develop more RNA-Seq SSRs. On one hand, the genic sequences used for developing SSR markers were highly conserved in sea buckthorn germplasm. On the other hand, the species and subspecies of sea buckthorn germplasm used in this study are limited to facilitate more polymorphism at SSR loci.

4) There is no comparison given between the varieties used in previous publication and the present one. Are you using common varities? If you are than SSR data must be same and must have been presented in previous MS already. This has not been mentioned in the MS.

Response: In previous publication, 31 accessions (common in the present one) were used for the validation of developed SSR markers. They included 6 accessions of ssp. sinensis, 14 accessions of ssp. mongolica and 11 hybrid accessions. They were selected according to their genetic origins and cultivated lands. In present study, the accessions were selected based on various fruit traits. The results of genetic relationship were different from that in the previous publication. That was supplemented in the discussion of revised manuscript.
‘In previous publication, the genetic relationship of 31 sea buckthorn accessions (also

contained in the 78 accessions) were analyzed based on 17 RNA-Seq SSRs [14]. However, the accessions of ssp. mongolica clustered in one group and those of ssp. sinensis and hybrid were in the other one. That revealed the genetic diversity is related on the genotypes and genetic backgrounds.'

Reviewer #1:

Specific comments
1. I think that in such bio-prospection studies sampling strategy is very crucial. The sampling method needs to explain that how these accessions were sourced. The MS needs elaboration on –
• How many individuals of a "variety" from each site were collected?
• Are these the random collections of registered varieties from the cultivated field in the five regions OR sampled from the wild?
• It is also not clear that how the hybrids were distinguished from parents while making collections.
• Do these sites differ in climatic conditions?
• What is the link of "origin" with oil content? Did you expect that there are bound to be differences because of differences in the climatic conditions of area of collection/cultivation of the same "variety/hybrid"?
Importantly instead of the term varieties the term accessions would be appropriate, as the authors have mentioned it in Table 1. According to the definition by The International Union for the Protection of New Varieties of Plants, "a variety must be recognizable by its characteristics, recognizably different from any other variety and remain unchanged through the process of propagation".
Do these two subspecies hybridize freely in nature and such hybrids have been characterized? This needs some population analysis like by using STRUCTURE, or at least there should be a note on the characterization of hybrids (including the features), even if they are procured form some Research Institute.

Response: The part of 'Plant materials' in original text was revised according to above advice.
• 235 individuals (2−5 ramet plants each accession) of 5−8 years in 5 growth sites were collected.
• These are registered accessions from the cultivated field and adapt to local environment.
• For the identification of the hybrid accessions, they are labelled and recorded with documents. Furthermore, most hybrid accessions and their parents are not in the same growth site. The parents of them are cultivated in the experimental field for hybridization.
• The growth sites differ in climatic conditions which are described in S2 Table.
• According to the results in this study, the oil contents in pulp and seeds are highest in ssp. mongolica accessions on average. That is the link of origin with oil content. In this study, we ignored the difference in the climatic conditions of cultivated fields for the sea buckthorn accessions we selected adapted local environment well.
• The authors agreed the opinion that the term accessions would be appropriate and all the term varieties were revised to the term accessions.
• These two subspecies hybridized by experiments in control which were performed in specialized experimental fields, And the hybrid accessions are characterized in the Research Institutes.

2. I don't understand the usage of term pulp/peel in the MS (also see page 15, line 251). As the entire fleshy region was separately used for extraction of oil from the "berries" (see Methods), the use of term pulp would be appropriate. One cannot expect to remove the epidermal peel especially during the mechanical homogenization process.
Response: The authors accepted the advice and the phrase 'pulp/peel' in the original text was revised to 'pulp' in the revised manuscript.

3. How the present study for the genetic diversity analysis of 78 cultivars is different from other previous studies? May be highlighted in the introduction. Authors may also highlight that trait: i.e. Oil yield was correlated with the "promising" accessions.
Response: The related content has been supplemented in the introduction of the

Powered by Editorial Manager® and ProduXion Manager® from Aries Systems Corporation

revised manuscript.

‘The diversity analysis helps understand the relationships between germplasm characters and genotype will improve the sea buckthorn germplasm to achieve higher production of higher quality for the important traits were correlated with the promising germplasm [19].
In present study, 78 accessions of sea buckthorn with large variation of fruit traits were selected as materials.’

4. Although attempt has been made of possible use of MAB in future, but it has not been justified with the discussion. For example, do the authors will depend on the same plants in the cultivated lands across the region or some mapping populations will be established. In former case GPS tagging of the individuals will be required for sourcing the material on regular basis and to establish the consistency of the trait.
Response: The results in present study yielded useful knowledge regarding the diversity and genetic relationships of sea buckthorn germplasm in northern China, and could therefore facilitates further studies, including selection of mapping populations and promising candidates, marker-trait association analysis based on establishing the consistency of the traits , and characterizing parents used in future breeding programs. The above information on possible use of MAB in future has been supplemented in the discussion of the revised manuscript.

Materials and Methods

5. Need to mention whether hundred-berry weight, hundred-seed weight and other dimensions were taken from mature or immature berries? In Supplementary figure 1 some samples are showing immature berries e.g. sample 65, 68 etc.
Response: The hundred-berry weight, hundred-seed weight and other dimensions should be taken from mature berries. So the berries of all accessions were collected from the end of July to mid-September, according to their ripening stages. But it is difficult to collected ripening fruits of 78 sea buckthorn accessions. The berries of several accession were harvested when they are approaching maturity. So the data error existed in the dimensions of several accessions. The authors admitted it and hope be understood at this point.

6. What do the ‘Berry Shape Indices’ refer to and what are its implications on the results/oil trait/ with genetic diversity. Provide any suitable reference if possible. (Page: 8, subsection: Morphological….)
Response: The berry shape index (BSI) is estimated by the ratio of BLD to BTD, also called length/width ratio in some studies, which indicates berry shape. According to the results in present study, the phenotypic characters (BLD, HBW, BSI, and BTD) of berries and oil traits in pulp showed close correlation ($r = 0.8725$, $p = 0.0000$) using CCA. The relevant literature is below. The results of it showed that the morphological traits established were consistent with those derived from the SSR markers in olive plant materials. The length/width ratio was one of the morphological traits of endocarp in that study.

Patricia RR, Carmen GB, Beatriz CG, Jesús SG, Isabel T. Genotypic and phenotypic identification of olive cultivars from northwestern Spain and characterization of their extra virgin olive oils in terms of fatty acid composition and minor compounds. Sci Hort. 2018; 232:269-279.

7. The usage of phrase ‘8 agronomic traits’ seems to be superfluous as these are the traits of berries itself. How the seed width is different from the seed thickness? The difference is not apparent. Table 2 and 3; as well as in text.
Response: For sea buckthorn, the traits of berries (including seeds) are very important for their economic value. The seed thickness could be regarded as the ‘height’ of seeds, which is a parameter of oilseed, e.g. olive.

8. The usage of abbreviation has not been followed see table 2 and 3. Table 2 is not necessary, may be omitted or shifted to Supplementary Data. In Tables SD is not mentioned.
Response: The authors accepted the advice. Table 2 was shifted to S4 Table. The data of ‘Mean

9. The reference is missing for the SB18-SB20 SSRs; in the text (Page 10, line 181).
Response: The SB18-SB20 SSRs were firstly reported in this study and no reference could be given for them.

Results

10. Results should be given in the format mean ± SD. Minimum and maximum can be given in supplementary tables.
Response: The authors accepted the advice and the results have been given in the format mean ± SD.

11. It is not clear from the table caption and content that whether values in the Table 4 is the minimum, maximum and mean values are representing the cumulative results of 78 varieties e.g. minimum in variety… and maximum in variety…. Need to mention in the results.
Response: The authors accepted the advice. The table caption in the Table 4 of original text is not clear because we want to use the abbreviation of 'minimum, maximum' but the notes were forgotten to give bellow the table. And these data have been mentioned in the results in the revised manuscript.

12. The results of CCA are driving a correlation between phenotypic traits and oil characteristics. The authors may use the information for total oil content (pulp+seed) or oil content in pulp and seeds separately for drawing any correlation. That would possibly help as a descriptor for the potential crop in identifying the elite/superior "variety" and further can be linked to genetic diversity.
Response: For the difference in the FAs composition between pulp oil and seed oil, the total oil content was not be used for drawing any correlation in this study. In practical production, the seed oil and pulp oil are separately extracted for their different functions. During the course of CCA, the factors in each data matrix would be analyzed by pairwise correlation analysis. So oil content in pulp and seeds separately for drawing any correlation is not necessary.

Discussion

13. Page:28, Line:449-453. The link of this part of discussion is lacking with the previous text.
Response: In the part of 'Introduction', the superiority of SSR markers was mentioned. The significance of developing SSR markers with RNA-Seq technique was also mentioned in it. The SSR markers used in this study are developed by RNA-Seq. All these description was the link of this part of discussion.

14. In conclusion part authors are concluding that this information may be useful for cultivar identification but initially they started their work for the varieties. Taxonomically these two are different entities.
Response: The authors agreed this opinion. The phrase 'cultivar identification' was revised to 'germplasm identification' and all the word 'varieties' were changed into 'accessions' in the revised manuscript according to the taxonomical definition.

Some suggestion:
1. The sequence of S1 and S2 table can be reversed as per the citation in the text.
Response: The good advice mentioned above is accepted by the authors. The tables were reversed in the revised manuscript.

2. Page:3, Line:54. Reference 1 is incorrect. The lead author here is Bartish I.V.
Response: The authors in reference 1 were corrected in the revised manuscript.

3. Page:3, Line:56-57. ….flavonoids [3-7]; ….products [8-10]. Here over-citation may be avoided.
Response: The authors accepted the advice and the references cited in the two sentences were cut down in the revised manuscript.

4. Page:3, Line:59. 'Sea buckthorn oil' instead of 'sea buckthorn oils'
Response: The phrase was corrected in the revised manuscript.

Powered by Editorial Manager® and ProduXion Manager® from Aries Systems Corporation

5. Page: 4, Line 74. Add a reference to the statement. The plant is able to avoid cold and is not resistant, because the leaves are shed under extreme cold condition in this plant. Even the species is not resistant to alkali too.
Response: The authors agreed this opinion and this sentence was revised to ‘Sea buckthorn adapts well to extreme conditions, including drought, salinity, alkalinity, and temperatures [12]’ in the revised manuscript.
12. Ruan CJ, Li H, Mopper S. Characterization and identification of ISSR markers associated with resistance to dried-shrink disease in sea buckthorn. Mol. Breeding. 2009; 24:255−268.

6. Page:4. Line:85. Use full form at first place ‘MAB’.
Response: The sentence was corrected in the revised manuscript and the full form ‘molecular marker-assisted breeding’ was used at first place ‘MAB’.

7. Page:5. Line:110. What was the premise of including two known elite varieties in the study? Any supportive reference(s) for the statement, and also mention the context in which these varieties are elite.
Response: The premise of elite varieties include high yield, good agronomic traits and strong adaptability to environment, etc. Some Chinese references support that Quyisike and Zhongguoshajiwild are elite cultivars. The word ‘elite’ in the sentence was deleted in the revised manuscript for no English reference supported it.

8. Page:12. Line:204-205. May be included in Material and Methods.
Response: The authors accepted the advice and the sentence ‘Minimum, maximum, mean, standard deviation (SD), and coefficient of variation (CV%) were recorded.’ was added in Material and Methods of the revised manuscript.

Reviewer #2:
1. The authors mention that 76 varieties were used. There is no mention of the different species they belonged to in M&M, although it has been mentioned later in the text and table. Incorporate that information in the M&M.
Response: The good advice mentioned above is accepted by the authors. The related information has been added in M&M of the revised manuscript.

2. Are these 76 different varieties or just different accessions? At many places they are being referred to as ‘cultivars’ also. Please correct accordingly in the text wherever mentioned.
Response: After careful consideration, the authors thought ‘accessions’ would be appropriate. The 'varieties' has been replaced into ‘accessions’ in the revised manuscript.

3. How variable are the climatic conditions of the three research institutes?
Response: The climatic conditions of different growth sites of sea buckthorn samples has been added in S2 Table of the revised manuscript, with the caption ‘Geographical and climatic conditions at different sample collection sites of sea buckthorn in northern China’.

4. Line 109: ‘………provided 76 varieties’. Does this mean that all the 76 were grown at all the 3 fields? There is no clarity on this aspect in the M&M. Most quantitative traits exhibit a huge variation across environments. To study the phenotypic variations it would have been much informative if all the 76 varieties were grown together across all the three fields. Why was that not considered?
Response: Among the 76 accessions of sea buckthorn samples, 12 were grown in the Institute of Selection and Breeding of Hippophae, 52 were grown in the Research Institute of Berry and 12 were grown in the Jiuchenggong Breeding Base of Sea Buckthorn. These accessions are able to adapt to local climate and screened to be excellent germplasm.
The authors agreed the opinion that most quantitative traits exhibit a huge variation across environments. We did the comparative analysis on fruit morphological traits of the same cultivars grown in different cultivated fields in our early studies and the data was complemented in the results (S4 Table) of the revised manuscript. The aim in this study is to further screen the elite accessions from the 78 accession with good adaption to the environments of cultivated fields and prepare for the next step of MAB.

In the follow-up study, the continuous observation of the environmental factors would be considered.

5. There is no mention of how these varieties were grown in the field, and data from how many plants were considered for the morphological and oil analysis. For eg. for hundred berry weight (HBW), berries were collected from how many different plants?
Response: The information has been supplemented in the introduction of the revised manuscript. The sea buckthorn samples in this study are collected from 235 individuals (2−5 ramet plants each accession) of 5−8 years in 5 growth sites. The berries of each accession were pooled and frozen as quickly as possible at −20 °C. When all plant materials were harvested, the berries were transferred to –50 °C for storage until analysis. The related information has been supplemented in the M&M and Table 1.

6. Line 137: For the oil extraction and FA analysis, the authors mention that 'each sample was analyzed three times'. Why weren't three biological replicates taken for this analysis?
Response: The authors are sorry for the incorrect expression. In this study, three biological replicates were taken for every analysis. The sentence is corrected in the revised manuscript.

7. Line 180-181: The authors have used 17 previously developed SSR markers and 3 newly developed SSR markers using RNA-Seq. What was the basis of selection of just 3 new markers from the RNA-Seq. Why weren't more markers deployed for the genetic characterization?
Response: The authors obtained many SSR sequences using RNA-Seq method and designed the primers to screen those SSR loci with polymorphism in sea buckthorn cultivars. We reported 17 developed SSR markers at first. In subsequent experiments, we screened 3 new SSR markers which also showed polymorphic amplification in sea buckthorn germplasm. RNA-Seq SSR loci with polymorphism in sea buckthorn germplasm were difficult to develop for that SSR markers derived from expressed region of genome showed high conservation to some extent in our study. That's why no more markers deployed for the genetic characterization in sea buckthorn at now.

8. Line 180: Please reframe the sentence. It appears that the authors have done RNA-seq to generate the 3 new SSR markers. Although, the RNA-Seq had been done in previous study from where the 17 SSR were also developed (Reference 17).
Response: The authors accepted the advice and the sentence has been changed into 'The Twenty polymorphic microsatellite loci (SSR) developed using RNA-Seq were evaluated and loci SB1-SB17 were previously published [17]' in the revised manuscript.

9. Instead of 'different origins' that has been used repeatedly by authors throughout the text and tables, I suggest use the two different species and hybrid accessions.
Response: The authors accepted the good advice. Some 'different origins' were changed into the 'two different subspecies and hybrid accessions' and the others were deleted in the revised manuscript.

10. Line 340: 'All the primers'. Reframe this line. All primers did not give 59 bands. A total of 59 bands were amplified.
Response: The sentence has been revised according to the advice in the revised manuscript.

11. Line 341: 'accounting for 86.44%' . Incomplete sentence, 86.44% of what??
Response: The sentence has been revised according to the advice in the revised manuscript.

12. Line 372: the 3 subgroups have been referred incorrectly. They are IIa, IIb and IIc.
Response: The names of 3 subgroups were corrected in the revised manuscript.

13. Line 421: 'in comparison of populations'. Statement not clear. Please reframe.
Response: The phrase has been changed to 'in population identification' in the revised manuscript.

14. Line 436: 'gene sequences'. Are all the SSR markers used genic in nature?

Powered by Editorial Manager® and ProduXion Manager® from Aries Systems Corporation

	Response: SSR can be divided into genomic SSRs and genic SSRs because of the resource of sequences used for SSR identification. Genic SSRs derived from transcriptome or expressed sequence tag sequences are located in expressed genes. These markers can be linked with important phenotypic characteristics through quantitative trait loci analysis. In this study, all SSR markers are genic SSRs.<br><br>15. Table 1: Could just be described as the 'Accessions of sea buckthorn used for the study'<br>Response: The authors accepted the good advice and the title of Table 1 was revised into the 'Accessions of sea buckthorn used for the study'.
**Additional Information:**	
**Question**	**Response**
**Financial Disclosure**<br><br>Enter a financial disclosure statement that describes the sources of funding for the work included in this submission. Review the submission guidelines for detailed requirements. View published research articles from *PLOS ONE* for specific examples.<br><br>This statement is required for submission and **will appear in the published article** if the submission is accepted. Please make sure it is accurate.<br><br>**Unfunded studies**<br>Enter: *The author(s) received no specific funding for this work.*<br><br>**Funded studies**<br>Enter a statement with the following details:<br>• Initials of the authors who received each award<br>• Grant numbers awarded to each author<br>• The full name of each funder<br>• URL of each funder website<br>• Did the sponsors or funders play any role in the study design, data collection and analysis, decision to publish, or preparation of the manuscript?<br>• **NO** - Include this sentence at the end of your statement: *The funders had no role in study design, data collection and analysis, decision to publish, or preparation of the manuscript.*<br>• **YES** - Specify the role(s) played.<br><br>* typeset	This research was financially supported by the Natural Science Foundation of China (NSFC)(Grant No. 31100489), which was received by He Li.<br>https://isisn.nsfc.gov.cn/egrantweb/
**Competing Interests**<br><br>Use the instructions below to enter a	The authors have declared that no competing interests exist.

competing interest statement for this submission. On behalf of all authors, disclose any competing interests that could be perceived to bias this work—acknowledging all financial support and any other relevant financial or non-financial competing interests.<br><br>This statement **will appear in the published article** if the submission is accepted. Please make sure it is accurate. View published research articles from *PLOS ONE* for specific examples.<br><br>**NO authors have competing interests**<br><br>Enter: *The authors have declared that no competing interests exist.*<br><br>**Authors with competing interests**<br><br>Enter competing interest details beginning with this statement:<br><br>*I have read the journal's policy and the authors of this manuscript have the following competing interests: [insert competing interests here]*<br><br>* typeset	
**Ethics Statement**<br><br>Enter an ethics statement for this submission. This statement is required if the study involved:<br><br>• Human participants<br>• Human specimens or tissue<br>• Vertebrate animals or cephalopods<br>• Vertebrate embryos or tissues<br>• Field research<br><br>Write "N/A" if the submission does not require an ethics statement.<br><br>General guidance is provided below. Consult the submission guidelines for detailed instructions. **Make sure that all information entered here is included in the**	N/A

**Methods section of the manuscript.**<br><br>**Format for specific study types**<br><br>**Human Subject Research (involving human participants and/or tissue)**<br>• Give the name of the institutional review board or ethics committee that approved the study<br>• Include the approval number and/or a statement indicating approval of this research<br>• Indicate the form of consent obtained (written/oral) or the reason that consent was not obtained (e.g. the data were analyzed anonymously)<br><br>**Animal Research (involving vertebrate animals, embryos or tissues)**<br>• Provide the name of the Institutional Animal Care and Use Committee (IACUC) or other relevant ethics board that reviewed the study protocol, and indicate whether they approved this research or granted a formal waiver of ethical approval<br>• Include an approval number if one was obtained<br>• If the study involved *non-human primates*, add *additional details* about animal welfare and steps taken to ameliorate suffering<br>• If anesthesia, euthanasia, or any kind of animal sacrifice is part of the study, include briefly which substances and/or methods were applied<br><br>**Field Research**<br><br>Include the following details if this study involves the collection of plant, animal, or other materials from a natural setting:<br>• Field permit number<br>• Name of the institution or relevant body that granted permission	
**Data Availability**<br><br>Authors are required to make all data underlying the findings described fully available, without restriction, and from the time of publication. PLOS allows rare exceptions to address legal and ethical concerns. See the PLOS Data Policy and FAQ for detailed information.	Yes - all data are fully available without restriction

A Data Availability Statement describing where the data can be found is required at submission. Your answers to this question constitute the Data Availability Statement and **will be published in the article**, if accepted.<br><br>**Important:** Stating 'data available on request from the author' is not sufficient. If your data are only available upon request, select 'No' for the first question and explain your exceptional situation in the text box.<br><br>Do the authors confirm that all data underlying the findings described in their manuscript are fully available without restriction?	
**Describe where the data may be found in full sentences. If you are copying our sample text, replace any instances of XXX with the appropriate details.**<br><br>• If the data are **held or will be held in a public repository**, include URLs, accession numbers or DOIs. If this information will only be available after acceptance, indicate this by ticking the box below. For example: *All XXX files are available from the XXX database (accession number(s) XXX, XXX.*).<br>• If the data are all contained **within the manuscript and/or Supporting Information files**, enter the following: *All relevant data are within the manuscript and its Supporting Information files.*<br>• If neither of these applies but you are able to provide **details of access elsewhere**, with or without limitations, please do so. For example:<br><br>*Data cannot be shared publicly because of [XXX]. Data are available from the XXX Institutional Data Access / Ethics Committee (contact via XXX) for researchers who meet the criteria for access to confidential data.*<br><br>*The data underlying the results*	All relevant data are within the manuscript and its Supporting Information files.

presented in the study are available from (include the name of the third party and contact information or URL).<br>• This text is appropriate if the data are owned by a third party and authors do not have permission to share the data.<br><br>* typeset	
Additional data availability information:	

Powered by Editorial Manager® and ProduXion Manager® from Aries Systems Corporation

Manuscript

Click here to access/download;Manuscript;Revised Manuscript without tracked changes_He Li_20190620.docx

Diversity in sea buckthorn (*Hippophae rhamnoides* L.) accessions with different origins based on morphological characteristics, oil traits, and microsatellite markers

He Li[1,2], Chengjiang Ruan[2]*, Jian Ding[2], Jingbin Li[2], Li Wang[2], Xingjun Tian[1,3]*

[1] School of Life Science, Nanjing University, Nanjing, P.R. China

[2] Key Laboratory of Biotechnology and Bioresources Utilization, Dalian Minzu University, Dalian, P.R. China

[3] Co-Innovation Center for Sustainable Forestry in Southern China, Nanjing Forestry University, Nanjing, P.R. China

* Corresponding authors

E-mail: ruan@dlnu.edu.cn (CR); tianxj@nju.edu.cn (XT)

Abstract

Sea buckthorn (*Hippophae rhamnoides*) is an ecologically and economically important species. Here, we assessed the diversity of 78 accessions cultivated in northern China using 8 agronomic characteristics, oil traits (including oil content and fatty acid composition) in seeds and pulp, and SSR markers at 23 loci. The 78 accessions included 52 from ssp. *mongolica*, 6 from ssp. *sinensis*, and 20 hybrids. To assess the phenotypic diversity of these accessions, 8 agronomic fruit traits were recorded and analyzed using principal component analysis (PCA). The first two PCs accounted for approximately 78% of the variation among accessions. The oil contents were higher in pulp (3.46-38.56%) than in seeds (3.88-8.82%), especially in ssp. *mongolica* accessions. The polyunsaturated fatty acids (PUFA) ratio was slightly lower in seed oil of hybrids (76.06%) than in ssp. *mongolica* (77.66%) and higher than in ssp. *sinensis* (72.22%). The monounsaturated fatty acids (MUFA) ratio of pulp oil of ssp. *sinensis* (57.00%) was highest, and that of ssp. *mongolica* (51.00%) was approximately equal to the ratio in the hybrids (51.20%). Using canonical correspondence analysis (CCA), we examined the correlation between agronomic traits and oil characteristics in pulp and seeds, respectively. Oil traits in pulp from different origins were correlated with morphological groupings (r = 0.8725, p = 0.0000). To assess the genotypic diversity, 23 SSR markers (including 17 loci previously reported) were used among the 78 accessions with 69 polymorphic amplified fragments obtained and an average PIC value of 0.2845. All accessions were classified into two groups based on the UPGMA method. The accessions of ssp. *sinensis* and ssp. *mongolica* were genetically distant.

The hybrid accessions were close to ssp. *mongolica* accessions. The 8 agronomic traits, oil characters in seed and pulp oils, and 23 SSR markers successfully distinguished the 78 accessions. These results will be valuable for cultivar identification and genetic diversity analysis in cultivated sea buckthorn.

Introduction

Sea buckthorn (*Hippophae rhamnoides* L.) is a winter hardy shrub that is naturally distributed throughout Asia and Europe. It is an economically valuable species, divided into eight subspecies. Of them, the ssp. *sinensis* and *mongolica* mainly distributed in Asia where they are abundant and commercially cultivated [1–2]. The fruits of sea buckthorn are rich in a variety of phytochemicals with physiological properties, such as lipids, carotenoids, ascorbic acid, tocopherols, and flavonoids [3–5]. The main applications for the fruits include food, cosmetics, and pharmaceutical products [6–7]. One of the most requested products for therapeutic practices is sea buckthorn oil, which is extracted from both seeds and pulp. The applications of sea buckthorn oil include healing of the skin, mucosa, and immune systems, especially in cancer and cardiovascular disease therapy [8–9].

Two important parameters in analyzing sea buckthorn oil quality are oil content and fatty acid composition (referred to here as ‘oil traits’ for simplicity). Sea buckthorn seed and pulp oils are considered the most valuable products of the berries with a unique fatty acid (FA) composition [10]. The seed oil contains omega-3 (α-linolenic acid) and omega-6 (linoleic acid) FAs, and the pulp oil is characterized by a high concentration

of FAs from the omega-7 group (e.g., palmitoleic acid). The seed oil is rich in unsaturated fatty acids (commonly 30-40% linoleic acid and 20-35% linolenic acid) [10]. The soft parts (pulp and peel) of the berries have a FA composition that differs from the seeds that is characterized by a high level of palmitoleic acid (16–54%), which is very uncommon in plants. The oil traits of sea buckthorn berries varies greatly according to their origin, based on the climatic and geological conditions of the growing areas [11].

Sea buckthorn adapts well to extreme conditions, including drought, salinity, alkalinity, and temperatures [12]. The vigorous vegetative reproduction and the strong, complex root system with nitrogen-fixing nodules make it an ~~optimal pioneer~~ plant for soil and water conservation. For these reasons, sea buckthorn was cultivated widely in arid and semiarid areas of China [13]. Due to small berries and thorns of native cultivars (ssp. *sinensis*), which have little economic value, the breeding of sea buckthorn has undergone different stages of development in China, such as introduction, domestication, seedling selection and artificial hybridization for elite accessions. The cultivars of ssp. *mongolica* (introduced from Russia and Mongolia), ssp. *sinensis* (China origin) and hybrids (ssp. *mongolica* × ssp. *sinensis*) are abundant in northern China [14]. However, as a perennial woody plant, traditional cross breeding that takes a long time and has low efficiency cannot meet the needs of modern production in sea buckthorn. It is essential for economic production to utilize molecular marker-assisted breeding (MAB) in sea buckthorn, especially to breed those accessions associated with desirable oil traits. An essential step in this process is the genetic analysis of sea

buckthorn germplasm. At present, molecular markers are mainly used for the analysis of genetic diversity, the taxonomic and geographic origin of cultivars, sex determination and population genetic structure in sea buckthorn [14–16]. SSR (simple sequence repeat, microsatellite) markers, with 1- to 6-bp DNA regions repeated in tandem, have been used in these analysis for their advantages of codominance, random distribution throughout the genome, easy detection, and high polymorphism and reproducibility [17]. Currently, an increasing number of microsatellite markers are being developed in sea buckthorn using high-throughput sequencing techniques for transcriptome datasets (RNA-Seq), which have become valuable resources for SSR discovery [14, 18].

The diversity analysis helps understand the relationships between germplasm characters and genotype will improve the sea buckthorn germplasm to achieve higher production with higher quality for the important traits were correlated with the promising germplasm [19].

In present study, 78 accessions of sea buckthorn with variation of fruit traits were selected as materials. The aim of this study is to report the phenotypic characteristics and oil traits in ~~pulp and seeds~~, and genetic diversity of the 78 sea buckthorn accessions in northern China, providing the identification foundation for MAB in sea buckthorn.

Materials and methods

Plant materials

Berries and leaves of 78 sea buckthorn accessions belong to ssp. *mongolica* (52 accessions), ssp. *sinensis* (6 accessions) and hybrids (ssp. *mongolica* × ssp. *sinensis*, 20

accessions) were collected from the end of July to mid-September in 2015. ~~These samples are from 235 individuals (2–5 ramet plants each accession) in different growth sites.~~ Table 1 summarizes information on the plant materials. Three research institutes located in northern China, the Institute of Selection and Breeding of *Hippophae* (42°26′N, 121°28′E; 380 m) in Fuxin, the Research Institute of Berry (47°14′N, 127°06′E; 202 m) in Suiling and the Jiuchenggong Breeding Base of Sea Buckthorn (39°40′N, 110°09′E; 1400 m) in Dongsheng, provided 76 accessions of sea buckthorn samples of all (Fig 1, S1 Table). The other two accessions, Quyisike and Zhongguoshaji*wild*, were harvested from cultivated fields in Qinghe (46°40′N, 90°22′E; 1218 m) and Datong (36°53′N, 101°35′E; 2800 m) (Fig 1, S1 Table, , S2 Table). These areas with various geographical and climatic conditions ranged between latitudes 36°53′N–47°14′N, longitudes 90°22′E–127°06′E, and altitudes 202–2800 m (S3 Table).

The young leaves of each plant were kept at −80 °C for use. The berries of each accession were pooled and frozen as quickly as possible at −20 °C. When all plant materials were harvested, the berries were transferred to –50 °C for storage until analysis.

Fig 1. Five cultivated lands of the 78 sea buckthorn accessions used in this study.

Table 1. Accessions of sea buckthorn usedc for the study.

No.	Accession name	Abbrev.[a]	Trees (no.)[b]	Collection site	ssp.[c]	No.	Accession name	Abbrev.[a]	Trees (no.)[b]	Collection site	ssp.[c]
1	Zhuangyuanhuang	ZYH	5	Fuxin	M	40	E13-10	E13-10	3	Suiling	M
2	Wucifeng	WCF	5	Fuxin	M	41	E13-11	E13-11	3	Suiling	M

3	Liusha-1	LS1	5	Fuxin	M	42	E13-14	E13-14	3	Suiling	M
4	Siberia rumianes	SR	4	Fuxin	M	43	HS-1	HS1	3	Suiling	M
5	Fangxiang	FX	2	Fuxin	M	44	HS-4	HS4	3	Suiling	M
6	Yalishanda-12	YLSD12	4	Fuxin	M	45	HS-9	HS9	3	Suiling	M
7	Jiuyuehuang	JYH	2	Fuxin	M	46	HS-10	HS10	3	Suiling	M
8	Nanren	NR	2	Fuxin	M	47	HS-12	HS12	3	Suiling	M
9	Botanical garden	BG	2	Fuxin	M	48	HS-14	HS14	3	Suiling	M
10	Zajiao-1	ZJ1	2	Fuxin	H	49	HS-18	HS18	3	Suiling	M
11	Zajiao-2	ZJ2	2	Fuxin	H	50	HS-20	HS20	3	Suiling	M
12	Zajiao-3	ZJ3	2	Fuxin	H	51	HS-22	HS22	3	Suiling	M
13	MZ-14	MZ14	3	Suiling	M	52	Xin’e-1	XE1	3	Suiling	M
14	Shoudu	SD	3	Suiling	M	53	Xin’e-2	XE2	3	Suiling	M
15	Fenlan	FL	3	Suiling	M	54	Xin’e-3	XE3	3	Suiling	M
16	Aertai	AET	3	Suiling	M	55	Zhongguoshaji	ZGSJ	3	Suiling	S
17	Chengse	CS	3	Suiling	M	56	EZ-4	EZ4	3	Suiling	H
18	Chuyi	CY	3	Suiling	M	57	Za-56	Za56	3	Suiling	H
19	Hunjin	HJ	3	Suiling	M	58	Za1-2	Za1-2	3	Suiling	H
20	Jinse	JS	3	Suiling	M	59	Za05-6	Za05-6	3	Suiling	H
21	Juren	JR	3	Suiling	M	60	Za05-20	Za05-20	3	Suiling	H
22	Xiangyang	XY	3	Suiling	M	61	Za05-21	Za05-21	3	Suiling	H
23	Yousheng	YS	3	Suiling	M	62	Za4	Za4	3	Suiling	H
24	Katuni	KTN	3	Suiling	M	63	Za13-19	Za13-19	3	Suiling	H
25	Wulangemu	WLGM	3	Suiling	M	64	Za13-25	Za13-25	3	Suiling	H
26	TF1	TF1	3	Suiling	M	65	Juda	JD	3	Dongsheng	S
27	TF2-13	TF2-13	3	Suiling	M	66	Jianpingdahuang	JPDH	3	Dongsheng	S
28	TF2-23	TF2-23	3	Suiling	M	67	Manhanci	MHC	3	Dongsheng	S
29	TF2-24	TF2-24	3	Suiling	M	68	Zhongxiongyou	ZXY	3	Dongsheng	S
30	TF2-36	TF2- 36	3	Suiling	M	69	Liaofuza	LFZ	3	Dongsheng	H
31	Suiji-1	SJ1	3	Suiling	M	70	Zaciyou-1	ZCY1	3	Dongsheng	H
32	Suiji-3	SJ3	3	Suiling	M	71	Zaciyou-10	ZCY10	3	Dongsheng	H

33	Suiji-4	SJ4	3	Suiling	M	72	Zaciyou-12	ZCY12	3	Dongsheng	H
34	HD-3	HD3	3	Suiling	M	73	Xinzaci-26	XZC26	3	Dongsheng	H
35	E10-06	E10-06	3	Suiling	M	74	Shiciyou-2	SCY2	3	Dongsheng	H
36	E10-34	E10-34	3	Suiling	M	75	Shiciyou-5	SCY5	3	Dongsheng	H
37	E10-42	E10-42	3	Suiling	M	76	Shiciyou-30	SCY30	3	Dongsheng	H
38	E10-47	E10-47	3	Suiling	M	77	Zhongguoshaji*wild*	ZGSJ*wild*	3	Datong	S
39	E13-00	E13-00	3	Suiling	M	78	Qiuyisike	QYSK	3	Qinghe	M

[a] Abbrev., abbreviation.

[b] Trees (no.) = number of trees.

[c] ssp., subspecies; M, ssp. *mongolica*; S, ssp. *sinensis*; H, hybrid (ssp. *mongolica* ♀ × ssp. *sinensis* ♂).

Morphological characteristics of fruit

Hundred berry weight (HBW) was the weight of 100 fresh berries after they were picked from bushes. Hundred seed weight (HSW) was the weight of 100 seeds after air drying at room temperature (25 °C) for 2 weeks [20]. There were three biological replicates for each measurement. The transverse and longitudinal diameters of berries (BTD and BLD) and the length, width and thickness of seeds (SL, SW and ST) were measured by micrometer calipers with over 20 measurements for each, on average. The berry shape indices (BSI) were estimated by the ratio of BLD to BTD. The data of minimum (Min), maximum (Max), mean ± SD (standard deviation), and coefficient of variation (CV%) were reported.

Oil extraction and FA analysis in seeds and ~~pulp~~

The methods of lipid extraction, transesterification (methylation) and purification of

methyl esters of the lipid extracts were described by Yang and Kallio [11]. Briefly, the seeds and pulp isolated from freeze-dried berries and lipids from the samples were extracted with chloroform/methanol (2:1, v/v) with mechanical homogenization of the tissues. The purified oils were filtered before the solvent was removed on a rotary evaporator. The lipids were weighed, and the oil contents (percentages) in seeds and pulp were calculated. Three biological replicates were taken for analysis. Lipids were stored in chloroform at −20 °C until analysis.

The oil (10 mg) was transesterified by sodium methoxide catalysis [11, 21]. It was dissolved in sodium-dried diethyl ether (1ml) and methyl acetate (20 μl). Then 1 M sodium methoxide in dry methanol (20 μl) was added, and the solution was agitated briefly and set still for 5 min at room temperature. The reaction ~~is~~ stopped by adding a saturated solution of oxalic acid in diethyl ether (30 μl) with brief agitation. The mixture ~~is~~ centrifuged at 1500 g for 2 min and the supernatant was dried in a gentle stream of nitrogen. Fresh hexane (1 ml) was added and the solution was filtered with microporous filtering films (0.22 μm) for analysis.

FAMEs were analyzed with ~~a~~ gas chromatography-tandem mass spectrometry (GC/MS/MS) system (model AxION® iQT™, PekinElmer, Shelton, CT, USA). Chromatographic separation was achieved using a DB-23 capillary column (60 m × 0.25 mm × 0.25μm; Agilent Technologies, Santa Clara, CA, USA) with the following temperature program: initial temperature 50 °C, hold for 1 min, heated to 175 °C at 25 °C/min, then heated to 215 °C at 3 °C/min and hold for 10 min, heated to 230 °C at 3 °C/min and hold for 5 min. The inlet was operated in split mode (1:20) at a

temperature of 250 °C with helium as the carrier gas at constant flow of 1.0 mL/min. The transfer line temperature was 215 °C, and the MS ion source was set to 230 °C. MS detection was carried out in electron impact (EI) ionization mode, scanning all masses from 45–400 amu. FAME components were identified based on mass spectral comparison with an external standard (Supelco 37 Component FAME Mix, Sigma-Aldrich, St. Louis, MO, USA) and previous studies [10−11]. The main fatty acid composition was expressed as a weight percentage of the total fatty acids from three replicates. The data of minimum, maximum, mean ± SD, and coefficient of variation were reported.

Statistical analysis

The data analysis for morphological traits and oil characteristics were performed with SPSS® 24.0 (IBM®). The following parameters were evaluated: mean, minimum value, maximum value, standard deviation (SD) and coefficient of variation (CV%). One-way ANOVA was used in the comparison of all traits among subsp. of *sinensis*, subsp. of *mongolica* and hybrids. Pearson correlation coefficients were calculated to analyze the relationship between pairs of 8 agronomic traits. Principal component analysis (PCA) was used to determine relationships among the accessions. In addition, a canonical correspondence analysis (CCA) was applied to the data between morphological characteristics and oil traits in different tissues (seeds and pulp).

DNA extraction and SSR analysis

Total genomic DNA was extracted from young leaves using the TaKaRa MiniBEST Plant Genomic DNA Extraction Kit (TaKaRa, Beijing, China) based on the manufacturer's protocol. Purity and quantity of extracted DNA were evaluated by gel electrophoresis and NanoDrop 2000 Spectrophotometer (Thermo Fisher Scientific, Waltham, MA, USA). Twenty-three polymorphic microsatellite loci (SSR) developed using RNA-Seq were evaluated and loci SB1-SB17 were previously reported [14] (S4 Table). PCR amplification was performed in 20 μL volumes containing 40 ng of DNA template, 1× PCR buffer, 1.5 mM $MgCL_2$, 0.15 mM of each dNTP (Takara, Dalian, China), 1.5 U of Taq polymerase (Takara, Dalian, China) and 0.5 μM of each primer. The PCR conditions included an initial denaturation at 94 °C for 2 min, 35 cycles of 30 s at 94 °C for denaturation, 30 s at 54–60 °C for annealing and 45 s at 72 °C for extension, with a final extension 7 min at 72 °C using a C1000 Touch™ Thermal Cycler (Bio-Rad, Berkeley, CA, USA). PCR products were electrophoresed on 8% non-denaturing polyacrylamide gels using a SE 600 Ruby Standard Dual Cooled Vertical Unit (GE Healthcare Life Sciences, Pittsburgh, PA, USA) and visualized by silver staining.

The microsatellites were scored as codominant markers for genetic diversity analysis. The number of alleles (Na), effective number of alleles (Ne), observed and expected heterozygosity (Ho and He), Shannon's information index (Is) and polymorphic information content (PIC) for each of the genic SSR markers were calculated using GenAlEx 6.5 [22–23] and PowerMarker version 3.25 [24] software packages. A genetic similarity matrix based on the proportion of shared alleles was

generated, and a UPGMA tree was constructed using PowerMarker. The dendrogram was displayed using MEGA 6 software [25] to reveal genetic relationships between the 78 sea buckthorn accessions.

Results

Morphological characterization of berries and seeds

Descriptive statistics analysis of 8 agronomic fruit traits for the 78 sea buckthorn accessions is shown in Table 2, S5 and S6 ~~Table~~. Relatively high CV values were observed in HBW, BLD, and HSW (> 20%). The highest coefficient of variation was observed in HBW (39.12%), which varied from 8.52 to 69.74 g. Analysis of variance (ANOVA, $p < 0.05$) showed that HBW of ssp. *mongolica* berries was 47.69 ± 11.03 g, which was much higher than ssp. *sinensis* berries (10.73 ± 1.54 g) and hybrids (31.44 ± 13.84 g). In hybrids, the HBW values were high in EZ4, Za56, Za1-2, Za05-6 and Za05-21(> 45 g), which were approximately the size of ssp. *mongolica* berries on average (~~S6 Table~~). BTD varied from 5.54 to 10.80 mm and BLD varied from 4.83 to 14.25 mm. In addition, BLD of berries from ssp. *mongolica* was higher than BTD, which was the opposite in berries of ssp. *sinensis*. According to BSI values, the berry shapes of the three groups were significantly different ($p = 0.000$): oblong berries of ssp. *mongolica* (1.35 ± 0.20), oblate for those of ssp. *sinensis* (0.90 ± 0.05) and circular for those of hybrids (1.08 ± 0.11). HSW varied from 0.61 to 2.19 g with an average of 1.45 g. Similar to HBW, there were significant differences for HSW among seeds from ssp. *mongolica*, ssp. *sinensis*, and hybrids ($p = 0.000$). SL varied from 2.00 to 3.49 mm

and SW varied from 2.98 to 7.43 mm. ST varied from 1.54 to 2.73 mm with an average of 1.93 mm. Overall, the agronomic characters of seeds (HSW, SL, SW, and ST) showed relatively low coefficients of variation, ranging between 11.50–24.33%; however, the berries (HBW, BTD, BLD, and BSI) had high coefficients of variation.

Table 2. Fruit traits of sea buckthorn berries of two different subspecies and hybrid accessions[a].

**Trait name**	**Abbrev.[b]**	**ssp. *mongolica***	**ssp. *sinensis***	**Hybrid**
Hundred berry weight (g)	HBW (g)	47.69 ±11.03a	10.73 ± 1.54c	31.44 ±13.84b
Berry transverse diameter (mm)	BTD (mm)	8.17 ± 0.99a	5.84 ± 0.23b	7.61 ± 1.24a
Berry longitudinal diameter (mm)	BLD (mm)	10.90 ± 1.48a	5.20 ± 0.19c	8.15 ± 1.18b
Berry shape index (%)	BSI (%)	1.35 ± 0.20	0.90 ± 0.05	1.08 ± 0.11
Hundred seed weight (g)	HSW (g)	1.60 ± 0.28a	0.79 ± 0.23c	1.28 ± 0.25b
Seed length (mm)	SL (mm)	5.91 ± 0.68a	3.31 ± 0.27c	4.64 ± 0.56b
Seed width (mm)	SW (mm)	2.76 ± 0.27a	2.18 ± 0.18c	2.52 ± 0.22b
Seed thickness (mm)	ST (mm)	1.98 ±0.18a	1.67 ± 0.16 b	1.86 ± 0.26a

[a] Values with different lower case letters (a–c) are significantly different at $p < 0.05$.

[b] Abbrev., Abbreviation.

In previous mutilocation trials in Suiling (47°14′N, 127°06′E; 202 m) and Dengkou (40°43′N, 106°30′E; 1053m, Inner Mongolia), the fruit characteristics of 11 large

berry accessions (AET, CS, CY, HJ, JS, JR, XY, YS, KTN, WLGM and SJ1) were comparatively analyzed (S7 Table), The HBWs of them in Suiling (38.33–67.59 g) were higher than those in Dengkou (32.87–63.85). For all the introduced cultivars, the HBWs in two experimental fields were lower than those in their country of origin, Russia. The phenotypic characteristics of sea buckthorn berries showed differences due to their origins, berry parts analyzed, climate and growing conditions. In this study, the 78 accessions were selected for their adaptabilities to growth sites.

PCA was performed using fruit characteristics (Fig 2). The first two principal components explained 78.11% of the total morphological variance. The first principle component (PC) accounted for 41.74% of the variance. It was associated with BTD, HBW, ST, HSW, and SW in descending order. Therefore, these traits were important attributes for the classification of sea buckthorn accessions. The second PC accounted for 36.37%, which is correlated with BSI, SL, and BLD in descending order. The plot shows the distribution of 78 sea buckthorn accessions on PC1 and PC2 (Fig 2). The ssp. *mongol*ica accessions with bigger berries tended to cluster together, mainly positive on PC2. Six accessions of ssp. *sinensis* with the smallest berries were negative on both PC1 and PC2. The hybrids were largely distributed between the above two groups. Some hybrids (including ZCY1, ZCY10, ZCY12, XZC26, SCY2, and SCY5) were close to the accessions from ssp. *sinensis*.

Fig 2. Two-dimensional scatter plot for the first two principal components (PC1 and PC2) based on the agronomic fruit characteristics of 78 sea buckthorn accessions. Numbers associated with

symbols are the variety codes as listed in Table 1. ▲ = ssp. *mongolica*; ● = ssp. *sinensis*; ◇ = hybrid.

Oil characterization in seeds and seedless parts

The oil characteristics of seeds and seedless parts (pulp and peel) among the 78 accessions are summarized in Tables 3 and Table 4. One special feature of sea buckthorn fruit was the high oil content in the pulp and peel (20.41%), in contrast to oil in seeds (8.82%). A higher coefficient of variation was observed in pulp oil content (42.72%) and varied over a wide range, from 3.46 to 38.56%. The pulp fraction of berries of ssp. *mongolica* had the highest oil content (24.68%) based on dry weight. The lowest pulp oil content (7.10%) on average was found in the berries of ssp. *sinensis*. In hybrids, the berries of ZJ2 contained 27.22% pulp oil, which slightly exceeded that of ssp. *mongolica* on average (S6 Table). Seed oil content varied from 3.88 to 12.75% with an average of 8.82%. The seeds of ssp. *mongolica* had the highest oil contents with an average of 9.46%, and those of the other two groups did not differ significantly.

Table 3. Oil characteristics of pulp and seeds of 78 sea buckthorn accessions.

Character	Pulp				Seed			
	**Min[a]**	**Max[b]**	**Mean ± SD[c]**	**CV[d] (%)**	**Min[a]**	**Max[b]**	**Mean ± SD[c]**	**CV[d](%)**
oil content	3.46	38.56	20.41 ± 8.72	42.72	3.88	12.75	8.82 ± 1.86	21.08
16:0	24.52	53.08	36.26 ± 4.83	13.32	3.84	11.77	6.55 ± 1.39	21.16
16:1n7	17.93	57.75	35.12 ± 7.64	21.76	tr[e]	tr[e]	tr[e]	
18:0	0.38	5.12	1.26 ± 0.70	55.58	1.41	4.58	2.16 ± 0.43	20.11

18:1n9	1.44	23.43	8.72 ± 4.72	54.13	3.05	25.95	13.25 ± 4.04	30.50
18:1n7	3.51	24.24	7.68 ± 4.09	53.28	0.45	2.38	1.20 ± 0.47	39.17
18:2n6	3.02	17.40	9.97 ± 3.18	31.91	34.22	52.75	42.17 ± 3.60	8.54
18:3n3	0.12	7.16	1.00 ± 1.03	102.83	21.37	47.16	34.67 ± 4.42	12.75

[a] Minimum value.

[b] Maximum value.

[c] Standard deviation.

[d] Coefficient of variation expressed in percentage.

[e] tr, trace (< 0.5%).

Table 4. Oil content and fatty acid composition in seeds and ~~the soft parts~~ of sea buckthorn berries of different origins[a].

	Pulp oil			Seed oil		
**Character**	**ssp. *mongolica***	**ssp. *sinensis***	**Hybrid**	**ssp. *mongolica***	**ssp. *sinensis***	**Hybrid**
oil content	24.68 ± 6.79 a	7.10 ± 3.28c	13.34 ± 4.85b	9.46 ± 1.56a	6.70 ± 1.32b	7.78 ±1.84b
16:0	37.68 ± 4.64a	29.39 ± 3.71b	34.62 ± 3.14a	6.52 ± 1.16	7.41 ± 1.55	6.38 ± 1.82
16:1n7	37.43 ±7.09a	23.65 ± 4.16b	32.55 ± 5.84a	tr[b]	tr[b]	tr[b]
18:0	1.08 ±0.69b	1.73 ± 0.64a	1.59 ± 0.57ab	2.13 ± 0.29	2.19 ± 0.44	2.23 ± 0.69
18:1n9	7.56 ±3.97b	16.67 ± 6.84a	9.33 ± 3.40b	12.62 ± 3.75b	16.37 ± 3.77a	13.96 ± 4.46ab
18:1n7	6.01 ±1.79c	16.68 ± 6.20a	9.32 ± 3.63b	1.07 ± 0.37b	1.80 ± 0.39a	1.37 ± 0.55b
18:2n6	9.55 ±2.76ab	8.34 ± 5.54b	11.53 ± 2.92a	42.10 ± 3.08	40.44 ± 4.06	42.87 ± 4.62
18:3n3	0.69 ±0.41b	3.54 ± 2.09a	1.07 ± 0.64b	35.56 ± 4.13a	31.78 ± 2.91b	33.20 ± 4.89 ab
MUFA	51.00 ±5.38b	57.00 ± 9.46a	51.20 ± 3.52b	13.69 ± 3.93b	18.18 ± 4.09a	15.33 ± 4.90ab
PUFA	10.24 ±2.98	11.89 ± 7.54	12.60 ±3.37	77.66 ± 4.31a	72.22 ±5.54b	76.06 ± 6.23ab

[a] Values with different lowercase letters (a–c) are significantly different at $p < 0.05$.

[b] tr, trace (< 0.5%).

For sea buckthorn, the FA composition in seed oil differed significantly from that in pulp oil. The proportions of linoleic (18:2n6), *α*-linolenic (18:3n3), oleic (18:1n9), palmitic (16:0), stearic (18:0) and vaccenic (18:1n7) acids were found from high to low in seed oil of most accessions (Table 4). Linoleic acid varied from 34.22 to 52.75% with an average of 42.17%. The proportion of *α*-linolenic acid varied from 21.37 to 47.16% with an average of 34.67%. High CV values were observed in oleic (30.50%) and vaccenic (39.17%) acids. Furthermore, the level of palmitoleic acid (16:1n7, < 0.5%) was extremely low in seed oil. The FA composition of sea buckthorn seeds were similar among berries of the two different subspecies and hybrid accessions. Small variations were found in the proportion of linoleic acid in seed oil (40.44 – 42.87%). Its proportion in hybrids were slightly higher than in ssp. *mongolica* (42.87% vs 42.10%), and had the highest value of the samples from the two different subspecies and hybrid accessions. *α*-Linolenic acid showed a little variation with a bigger proportion in ssp. *mongolica* than in ssp. *sinensis* (35.56% vs 31.78%). A higher proportion of palmitic (7.41% vs 6.38%) and oleic (16.37% vs 13.96%) acids and a lower proportion of stearic acid (2.19% vs 2.23%) were discovered between the accessions of ssp. *sinensis* and hybrids. The polyunsaturated fatty acids (PUFA) ratio in hybrids (76.06%) was slightly lower than it was in ssp. *mongolica* (77.66%) and higher than it was in ssp. *sinensis* (72.22%). Some hybrids (including ZJ1, Za1-2, Za13-25, Za05-6, LFZ, and ZCY12) contained a high proportion of PUFA (> 80%) in seed oil, which was more than the average level of ssp. *mongolica* accessions (S6 Table).

In pulp oil, the dominant FAs were palmitoleic, palmitic, linoleic, oleic, and vaccenic acids (Table 3). Major differences were observed in the proportion of palmitoleic (17.93-57.75%), oleic (1.44-23.43%) and vaccenic (3.51-24.24%) acids. The special feature of pulp oil is high proportions (> 35%) of palmitoleic and palmitic acids. Compared to ssp. *sinensis*, ssp. *mongolica* contained a higher proportion of palmitoleic and palmitic acids in the berry pulp ($p < 0.05$) (Table 4). In particular, the proportions of oleic and vaccenic acids were highest in ssp. *sinensis*, much higher than those in ssp. *mongolica* and hybrid accessions. The relative levels of *α*-linolenic and stearic acids in pulp of ssp. *sinensis* were higher than ssp. *mongolica* ($p < 0.05$) (Table 4). For hybrids, the proportions of most fatty acids were between ssp. *mongolica* and ssp. *sinensis* accessions, except for linoleic acid. Similar to the results in seed oils, the hybrids had the highest proportions of linoleic acid (11.53%) and PUFA (12.60%). The monounsaturated fatty acids (MUFA) ratio in pulp oil of ssp. *sinensis* (57.00%) was highest and that of ssp. *mongolica* (51.00%) was almost equal to hybrids (51.20%). In hybrids, the pulp oil of SCY2 contained 39.16% palmitoleic acid, and the content of MUFA was 60.77%, which was higher than it was in ssp. *sinensis* (S6 Table).

Correlations among the agronomic traits and oil characters

Canonical analyses allow direct comparisons of two data matrices. All sea buckthorn accessions were represented in a two-dimensional space using CCA between phenotypic traits and oil characteristics (Fig 3). For berries of the two different subspecies and hybrid accessions, phenotypic characters (BLD, HBW, BSI, and BTD)

of berries and oil traits in pulp showed close correlation (r = 0.8725, p = 0.0000). Based on CCA, accessions of ssp. *mongolica* were clustered on the upper side (mainly positive on D1 and D2), those of ssp. *sinensis* on the other, and the hybrids in the middle in Fig 3A. The positioning of samples in the first dimension was mostly related to differences in their berry characteristics that were primarily provided by a marker of BLD. The second dimension indicated differences in oil contents and FA compositions of pulp oil among sea buckthorn accessions. Differences between pulp oil traits were primarily related to percentages of oil content, 16:0 and 16:1n7, which were highest in ssp. *mongolica*, followed by hybrids, and lowest in ssp. *sinensis*. For seeds of 78 accessions, phenotypic characters (SL, SW, ST, and HSW) and seed oil traits were correlated (r = 0.7482, p = 0.0000). The positioning of samples was staggered (Fig 3B), which reflected that all seed samples had relatively little variation among phenotypic traits and oil characteristics. These results verified the previous analysis (Table 2 and Table 3).

Fig 3. Canonical correspondence analysis of phenotypic traits (A. berry; B. seed) and oil characteristics (A. pulp oil; B. seed oil) of sea buckthorn germplasms. D1, Dimension 1; D2, Dimension 2. ▲ = ssp. *mongolica*; ● = ssp. *sinensis*; ◇ = hybrid.

SSR diversity

Twenty pairs of RNA-Seq SSR primers with good amplification and band stability were used in 78 accessions of sea buckthorn. A total of 69 bands were amplified using the 23

primer pairs, of which 59 were polymorphic, accounting for 85.51% of all. The number of amplified bands per locus ranged from 2 to 5, averaging 3, and the number of effective alleles (Ne) ranged from 1.0392 to 3.1049, averaging 1.6602 (Table 6). SB2, SB3, SB5, SB6, SB8, SB13, SB16 and SB23 were informative SSR loci, each revealing more than four effective alleles distributed among all of the accessions. Compared with the observed allele number (Na), the number of effective alleles and their average values were lower, which was caused by the uneven distribution of gene frequencies in SSR loci. In genetic diversity analysis, observed heterozygosity (Ho) ranged from 0.0385 to 0.7949, with an average of 0.2965; expected heterozygosity (He) ranged from 0.0377 to 0.6779, with an average of 0.3291, and the Shannon index (Is) ranged from 0.0950 to 1.2152, with an average of 0.5681. The value of polymorphism information content (PIC), regarded as discriminating power, varied from 0.0370 to 0.6174, with an average of 0.2845. Loci SB06 (PIC = 0.6174) and SB08 (PIC = 0.5820) showed higher effectiveness because of their high informativity, which could be used to construct the fingerprint map of sea buckthorn germplasm. The characteristics of these 23 loci in genetic diversity analysis of sea buckthorn germplasm are shown in Table 5.

Table 5. Characterization of 20 polymorphic SSR markers in the 78 sea buckthorn accessions.

Loci code	Na	Ne	Ho	He	PIC	Is
SB1	3	1.2745	0.2436	0.2154	0.2025	0.3956

SB2	4	1.1382	0.1282	0.1214	0.1166	0.2791
SB3	4	2.2372	0.4615	0.5530	0.4627	0.9090
SB4	2	1.5006	0.2692	0.3336	0.2779	0.5160
SB5	4	2.1129	0.3333	0.5267	0.4735	0.9288
SB6	4	3.1049	0.7051	0.6779	0.6174	1.2152
SB7	2	1.0799	0.0769	0.0740	0.0712	0.1630
SB8	5	2.8490	0.3846	0.6490	0.5820	1.1890
SB9	2	1.1509	0.1410	0.1311	0.1225	0.2550
SB10	3	1.5350	0.2949	0.3485	0.3114	0.6253
SB11	2	1.9287	0.1667	0.4815	0.3656	0.6745
SB12	3	1.2430	0.2179	0.1955	0.1753	0.3687
SB13	4	2.1644	0.4231	0.5380	0.4392	0.8687
SB14	2	1.9987	0.3077	0.4997	0.3750	0.6928
SB15	2	1.0662	0.0641	0.0620	0.0601	0.1418
SB16	4	1.4567	0.1923	0.3135	0.2956	0.6427
SB17	2	1.4175	0.3590	0.2945	0.2512	0.4706
SB18	2	1.0392	0.0385	0.0377	0.0370	0.0950

SB19	3	1.0804	0.0641	0.0744	0.0724	0.1804
SB20	2	1.1803	0.1667	0.1528	0.1411	0.2868
SB21	3	1.9123	0.7308	0.4771	0.3802	0.7318
SB22	3	1.2905	0.2564	0.2251	0.2025	0.4084
SB23	4	2.4239	0.7949	0.5874	0.5102	1.0284

Na, observed number of alleles; Ne, effective number of alleles; Ho, observed heterozygosity; He, expected heterozygosity; PIC, polymorphism information content; Is, Shannon's information index.

Genetic relationships among sea buckthorn germplasm

The sea buckthorn germplasm in this study originated from ssp. *mongolica* (52 accessions), ssp. *sinensis* (6 accessions) and hybrids (20 accessions). Using 23 polymorphic SSR markers, the UPGMA dendrogram based on the proportion of shared alleles was constructed to assess the genetic relationships between the 78 accessions (Fig 4). The results showed that all the accessions could be divided into two groups (I and II). The accessions of ssp. *sinensis* (JD, ZGSJ, MHC, ZGSJ^{wild}, JPDH and ZXY) were clustered into group I. These accessions had closer relationships, despite great geographic differences. The second group was divided into 3 subgroups, namely, IIa, IIb, and IIc. The 20 hybrid accessions were all clustered into IIa. Subgroup IIb and IIc contained all the accessions of ssp. *mongolica* (introduced from Russia and Mongolia). Subgroup IIb included 6 accessions, namely WCF, LS1, QYSK, FX, SR, MZ14. The rest accessions of ssp. *mongolica* were clustered into IIc. Among them, KTN, WLGM,

HS4, HS9, HS10, HS12, HS14, HS18, HS20, HS22, WCF, FX and MZ14 composed one sub-subgroup. SJ3, ZYH, SD, NR, FL, XE2, XE3, JYH and YLSD12 showed close relationships. Other 23 accessions clustered into the third sub-subgroup. Overall, the relationship between ssp. *mongolica* and ssp. *sinensis* was relatively distant. The hybrids are close to ssp. *mongolica* which their female parents belonged to.

Fig. 4. UPGMA dendrogram of sea buckthorn germplasm based on SSR data (sample abbreviations described in Table 1). ▲ = ssp. *mongolica*; ● = ssp. *sinensis*; ◇ = hybrid.

Discussion

Morphological characteristics, biochemical traits, and microsatellite markers have been used for germplasm identification and genetic diversity analysis in many horticultural plants [26–27]. The diversity at morphological, biochemical, and molecular levels of 78 sea buckthorn accessions, composed of 52 from ssp. *mongolica*, 6 from ssp. *sinensis*, and 20 hybrids, were investigated.

The morphological characterization of plant materials with desired traits is an essential step for the effective use of germplasm [28]. Here, 8 important agronomic traits were measured among 78 sea buckthorn accessions, and a considerable amount of variation in morphological traits was found. The berry sizes of berries from the two different subspecies and hybrid accessions were significantly different according to the HBW value (p = 0.000). Compared to ssp. *sinensis* berries, ssp. *mongolica* berries were much bigger on average. The berry size of hybrid accessions were between the two

subspecies. In PC analysis, we plotted 2D plots with PC1 and PC2 scores of phenotypes (Fig 2). PC1 was mainly related with BTD and HBW, which explained the largest portion of the variance in 78 accessions. The distribution of 78 accessions on PC1 and PC2 was consistent with their agronomic characters (Fig 2). These results estimating morphological traits are valuable tools for identifying variation among plant germplasm [26].

For biochemical traits, oil content and FA composition in seeds and seedless parts were selected for their important roles in human health. The oil of sea buckthorn seems to be a good source of unsaturated fatty acids. The seed oil is rich in PUFA, including linoleic and α-linolenic acids. The proportion of PUFA did not differ significantly among berries from three origins, despite the differences in some morphological characteristics and in growth conditions. These results were consistent with the previous studies [10]. The results of the present study and previous investigations also suggested that the berries of ssp. *mongolica* were a good source of palmitic and palmitoleic acids in pulp oil and those of ssp. *sinensis* were a good source of oleic acid, both in seeds and pulp [29]. Although carefully selected for intersubspecies crosses, some hybrids displayed elite oil traits. For example, the proportion of MUFA in pulp of SCY2 and of PUFA in seeds of 6 accessions (including ZJ1, Za1-2, Za13-25, Za05-6, LFZ, and ZCY12) exceeded the average level of ssp. *mongolica* accessions, the subspecies of one of their parents belonged to. These results demonstrate the effectiveness of traditional cross breeding in the improvement of native accessions (ssp. *sinensis*), even though it is time-consuming and has low efficiency.

Previous studies found that berry size is a useful indicator of Vc, sugars and acids in population identification [19, 30]. The nutrients in the seedless fraction were more concentrated in the small berries of ssp. *sinensis* than in the large berries of ssp. *mongolica* [29]. In the present study, we analyzed the correlation between agronomic characteristics and oil traits at different levels (seed and pulp) by CCA. The results showed phenotypic characteristics (BLD, HBW, BSI, and BTD) of berries and oil traits in pulp were positively correlated ($r = 0.8725$, $p = 0.0000$). BLD, as a promising marker, provided the primary difference in CCA. Our results illustrated that berry size had different correlations with various biochemical characteristics in sea buckthorn.

Variation of phenotypic traits among germplasms may be attributed to differences in genetic backgrounds, geographical location, climate, harvest period and berry maturity, while molecular markers are independent of environmental condition and growth stage [31]. Twenty polymorphic SSR markers were used to identify 78 sea buckthorn accessions. The selected 23 SSR markers detected 2–5 alleles, and their PIC values ranged from 0.1166 to 0.6155 and had an average of 0.3249. The PIC mean value was significantly lower than that of RAPD, ISSR and SRAP markers previously reported [15−16, 32], suggesting that the gene sequences of these SSR markers were conserved in sea buckthorn germplasm.

Based on UPGMA, the 78 accessions were classified into two groups. There is a large genetic distance between accessions of ssp. *sinensis* and ssp. *mongolica*. The hybrids were in between and rather close to ssp. *mongolica* accessions. Coincidentally, these hybrids were also between ssp. *sinensis* and ssp. *mongolica* accessions on the

PCA plot based on 8 agronomic characters. This result illustrated that the diversity of morphological characters could reflect genetic diversity and be used as markers in agronomy. Ruan et al. [15] assessed 14 Chinese, Russian and Mongolian sea buckthorn accessions using RAPD markers and obtained similar results. In previous publication, the genetic relationship of 31 sea buckthorn accessions (also contained in this study) were analyzed based on 17 RNA-Seq SSRs [14]. However, the accessions of ssp. *mongolica* clustered in one group and those of ssp. *sinensis* and hybrids were divided in the other one. That revealed the genetic diversity relied on the diversity of genotypes and genetic backgrounds.

With the continuous development of high-throughput sequencing technology, transcriptome databases have become a powerful resource for SSR mining. More and more RNA-Seq SSRs have been developed and applied to the study of species genetic diversity and population genetic structure [33–34]. The SSRs obtained by transcriptomes are associated with many important quantitative traits [35].

The results in present study yielded useful knowledge regarding the diversity and genetic relationships of sea buckthorn germplasm in northern China, and could therefore facilitates further studies, including selection of mapping populations and promising candidates, marker-trait association analysis based on establishing the consistency of the traits , and characterizing parents used in future breeding programs.

Conclusion

In the present study, 8 phenotypic characteristics, oil traits in seeds and seedless parts,

and 23 SSR markers successfully distinguished all 78 sea buckthorn accessions. In PC analysis, BTD and HBW in the first PC were the most important characteristics for distinguishing the accessions. The agronomic traits of berries were closely correlated with the oil content and FA composition in pulp by CCA. This information will be valuable for germplasm identification and genotypic diversity analysis in *Hippophae rhamnoides*.

Supporting information

S1 Fig. 78 berry samples used in this study. Numbers are the variety codes as listed in Table 1.

(TIF)

S2 Fig. Total ion flow chromatography of 37 FAMEs Mix (A) and FAMEs in pulp oil in MHC (B).

(TIF)

S1 Table. Samples of sea buckthorn grouped according to different genetic backgrounds.

(DOCX)

S2 Table. Characterization of hybrids of sea buckthorn accessions studied.

(DOCX)

S3 Table. Geographical and climatic conditions at different sample collection sites of sea buckthorn in northern China.

(DOCX)

S4 Table. Primer sequences, annealing temperature, and estimated allelic size of 20 SSR markers.

(DOCX)

S5 Table. Descriptive statistics for morphological traits of berries and seeds among the sea buckthorn accessions studied.

(DOCX)

S6 Table. The morphological characteristics and oil traits of pulp and seeds of 78 sea buckthorn accessions studied.

(XLSX)

S7 Table. Fruit traits and Vc contents of large berry accessions of sea buckthorn in two experimental fields (located in Suiling and Dengkou).

(DOCX)

S8 Table. Allele combinations obtained at the 20 microsatellite loci in 78 sea buckthorn accessions.

(TXT)

Acknowledgements

The authors are grateful to Hai Guo (Jiuchenggong Breeding Base of Sea Buckthorn) and Jun Zhang (Institute of Selection and Breeding of *Hippophae*) for the collection of plant materials.

References

1. Bartish IV, Jeppsson N, Nybom H, Swenson U. Phylogeny of *Hippophae* (Elaeagnaceae) inferred from parsimony analysis of chloroplast DNA and morphology. Syst Bot. 2002; 27:41–54.
2. Swenson U and Bartish IV. Taxonomic synopsis of *Hippophae* (Elaeagnaceae). Nord J Bot. 2002; 22:369–374.
3. Teleszko M, Wojdyło A, Rudzińska M, Oszmiański J, Golis T. Analysis of Lipophilic and Hydrophilic Bioactive Compounds Content in Sea Buckthorn (*Hippophaë rhamnoides* L.) Berries. J Agric Food Chem. 2015; 63:4120−4129.
4. Pop MR, Weesepoel Y, Socaciu C, Pintea A, Vincken JP, Gruppen H. Carotenoid composition of berries and leaves from six Romanian sea buckthorn (*Hippophae rhamnoides* L.) varieties. Food Chem. 2014; 147:1−9.
5. Raffo A, Paoletti F, Antonelli M. Changes in sugar, organic acid, flavonol and carotenoid composition during ripening of berries of three seabuckthorn (*Hippophae rhamnoides* L.) cultivars. Eur Food Res Technol. 2004; 219:360−368.
6. Bal ML, Meda V, Naik NS, Satya S. Sea buckthorn: A potential source of valuable nutrients for nutraceuticals and cosmoceuticals. Food Res Int. 2011; 44:1718−1727.
7. Suryakumar G, Gupta A. Medicinal and therapeutic potential of Sea buckthorn (*Hippophae rhamnoides* L.). J Ethnopharmacol. 2011; 138:268−278.
8. Grey C, Widén C, Adlercreutz P, Rumpunen K, Duan RD. Antiproliferative effects of sea buckthorn (*Hippophae rhamnoides* L.) extracts on human colon and liver cancer cell lines. Food Chem.2010; 120: 1004−1010.
9. Xu YJ, Kaur M, Dhillon SR, Tappia SP, Dhalla SN. Health benefits of sea buckthorn for the prevention of cardiovascular diseases. J Func Foods. 2011; 3:2−12.

10. Yang B and Kallio H. Composition and Physiological Effects of Sea Buckthorn Lipids, Trends Food Sci Technol. 2002; 13:160–167.

11. Yang B and Kallio H. Fatty acid composition of lipids in sea buckthorn (*Hippophaë rhamnoides* L.) berries of different origins. J. Agric. Food Chem. 2001; 49:1939−1947.

12. Ruan CJ, Li H, Mopper S. Characterization and identification of ISSR markers associated with resistance to dried-shrink disease in sea buckthorn. Mol. Breeding. 2009; 24:255−268.

13. Ruan CJ, Teixeira da Silva JA, Jin H, Li H, Li DQ. Research and biotechnology in sea buckthorn (*Hippophae* spp.). Medicinal and Aromatic Plant Science and Biotechnology. 2007; 1: 47−60.

14. Li H, Ruan CJ, Wang L, Ding J, Tian XJ. Development of RNA-Seq SSR markers and application to genetic relationship analysis among sea buckthorn germplasm. J Amer Soc Hort Sci. 2017; 142(3):200-208.

15. Ruan CJ. Genetic relationships among some sea buckthorn cultivars from China, Russia and Mongolia using RAPD markers. Sci Hort. 2004; 101:417–426.

16. Li H, Ruan CJ, Teixeira da Silva J, Liu BQ. Associations of SRAP markers with dried-shrink disease resistance in a germplasm collection of sea buckthorn (*Hippophae* L.). Genome. 2010; 53:447–457.

17. Kalia RK, Rai MK, Kalia S, Singh R, Dhawan AK. Microsatellite markers: An overview of the recent progress in plants. Euphytica. 2011; 177:309–334.

18. Jain A, Chaudhary S, Sharma PC. Mining of microsatellites using next generation sequencing of seabuckthorn (*Hippophae rhamnoides* L.) transcriptome. Physiol. Mol. Biol. Plants. 2014; 20:115–123.

19. Patricia RR, Carmen GB, Beatriz CG, Jesús SG, Isabel T. Genotypic and phenotypic identification of olive cultivars from northwestern Spain and characterization of their extra

virgin olive oils in terms of fatty acid composition and minor compounds. Sci Hort. 2018; 232:269-279.

20. Tang X and Tigerstedt PMA. Variation of physical and chemical characters within an elite sea buckthorn (*Hippophae rhamnoides* L.) breeding population. Sci. Hort. 2001; 88(3):203–214.

21. Christie WW. A simple procedure for rapid transmethylation of glycerolipids and cholesteryl esters. J Lipid Res. 1982; 23:1072-1075.

22. Peakall R and Smouse PE. GENALEX 6: Genetic analysis in Excel. Population genetic software for teaching and research. Mol Ecol Notes. 2006; 6:288–295.

23. Peakall R and Smouse PE. GenAlEx 6.5: Genetic analysis in Excel. Population genetic software for teaching and research – An update. Bioinformatics. 2012; 28:2537–2539.

24. Liu K and Muse SV. PowerMarker: An integrated analysis environment for genetic marker analysis. Bioinformatics. 2005; 21:2128–2129.

25. Tamura SK, Peterson GD, Filipski A, Kumar S. MEGA6: Molecular evolutionary genetics analysis version 6.0. Mol Biol Evol. 2013; 30:2725–2729.

26. Lee ON and Park HY. Assessment of genetic diversity in cultivated radishes (*Raphanus sativus*) by agronomic traits and SSR markers. Sci Hort. 2017; 223:19-30.

27. Goodarzi S, Khadivi A, Abbasifar A, Akramian M. Phenotypic, pomological and chemical variations of the seedless barberry (*Berberis vulgaris* L. var. asperma). Sci Hort. 2018; 238:38–50.

28. Santos RC, Pires JL, Correa RX. Morphological characterization of leaf, flower, fruit and seed traits among Brazilian *Theobroma* L. species. Genet. Resour. Crop Evol. 2012; 59:327–345.

29. Kallio H, Yang B, Peippo P, Tahvonen R, Pan R. Triacylglycerols, glycerophospholipids, tocopherols and tocotrienols in sea buckthorn *Hippophae rhamnoides* L. ssp. *sinensis* and ssp.

mongolica berries and seeds. J. Agric. Food Chem. 2002; 50:3004–3009.

30. Kallio H, Yang B, Peippo P. Effects of Different Origins and Harvesting Time on Vitamin C, Tocopherols, and Tocotrienols in Sea Buckthorn (*Hippophae1 rhamnoides*) Berries. J. Agric. Food Chem. 2002; 50:6136−6142.

31. Ali M, Rajewski J, Baenziger P, Gill K, Eskridge KM, Dweikat I. Assessment of genetic diversity and relationship among a collection of US sweet sorghum germplasm by SSR markers. Mol. Breed. 2008; 21:497–509.

32. Li, H., C.J. Ruan, and J.A. Teixeira da Silva. 2009. Identification and genetic relationship based on ISSR analysis in a germplasm collection of sea buckthorn (*Hippophae* L.) from China and other countries. Sci. Hort. 123:263–271.

33. Liu YL, Zhang PF, Song ML, Hou JL, Qing M, Wang WQ, Liu CS. Transcriptome analysis and development of SSR molecular markers in *Glycyrrhiza uralensis* Fisch. PLoS One. 2015; 10:e0143017. doi:10.1371/journal.pone.0143017

34. Zhang LW, Li YR, Tao AF, Fang PP, Qi JM. Development and characterization of 1,906 EST-SSR markers from unigenes in jute (*Corchorus* spp.), PLoS One. 2015; 10(10):e0140861. doi:10.1371/journal.pone.0140861

35. Ramchiary N, Nguyen VD, Li X, Hong CP, Dhandapani V, Choi SR, et al. Genic microsatellite markers in *Brassica rapa*: Development, characterization, mapping, and their utility in other cultivated and wild *Brassica* relatives. DNA Res. 2011; 18:305–320.

Figure

Click here to access/download;Figure;Fig_1.tif

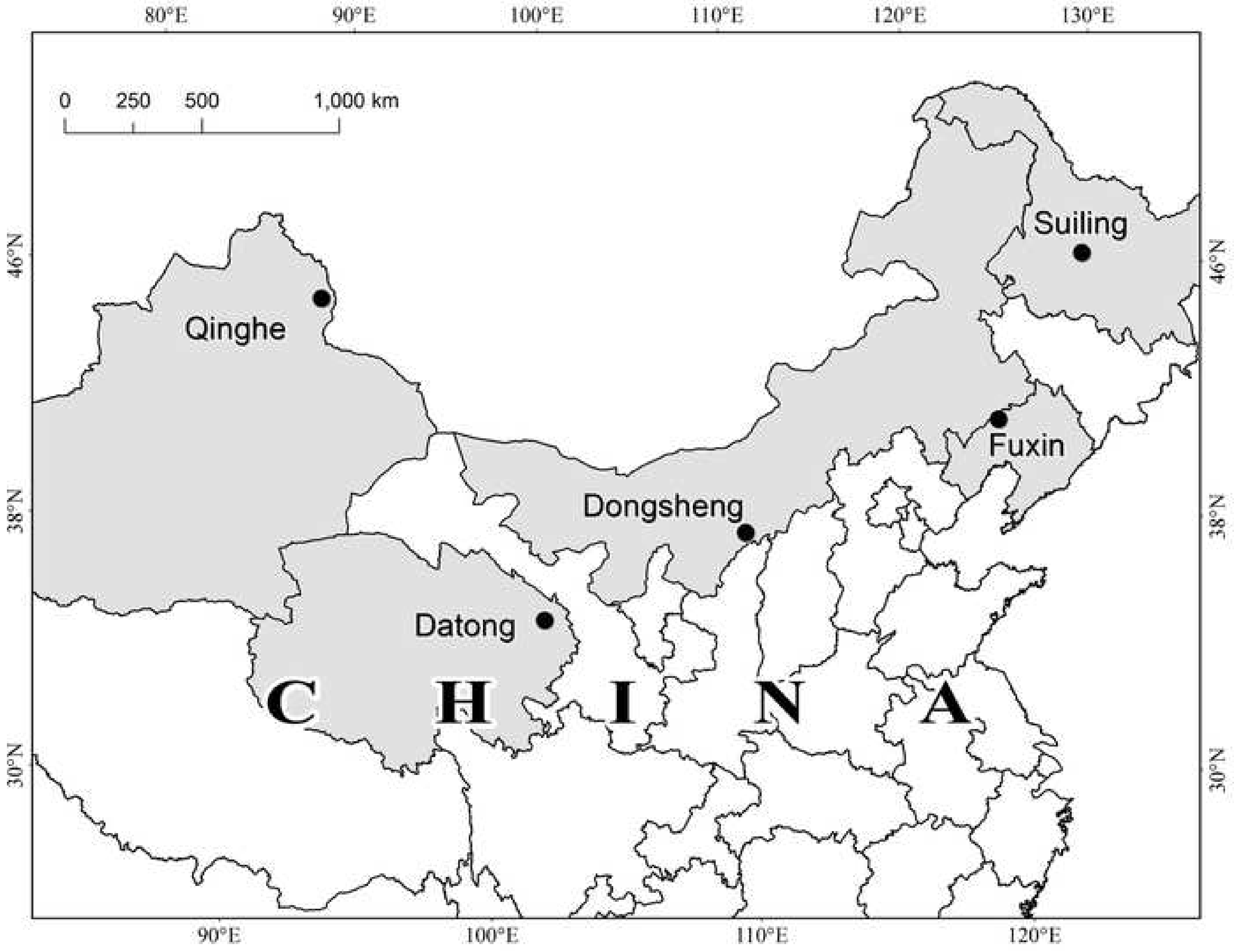

Figure

Click here to access/download;Figure;Fig_2.tif

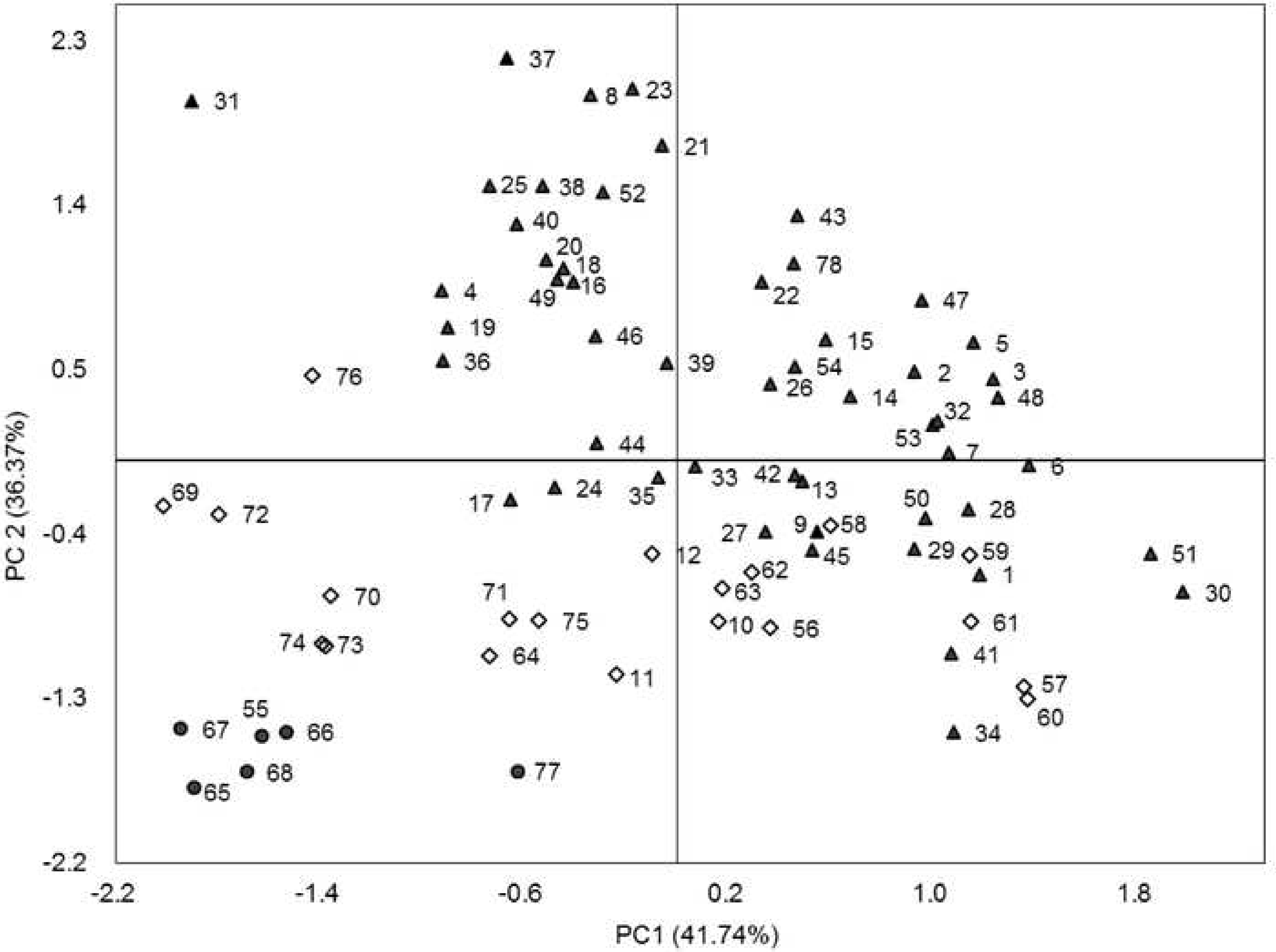

 Click here to access/download;Figure;Fig_3.tif

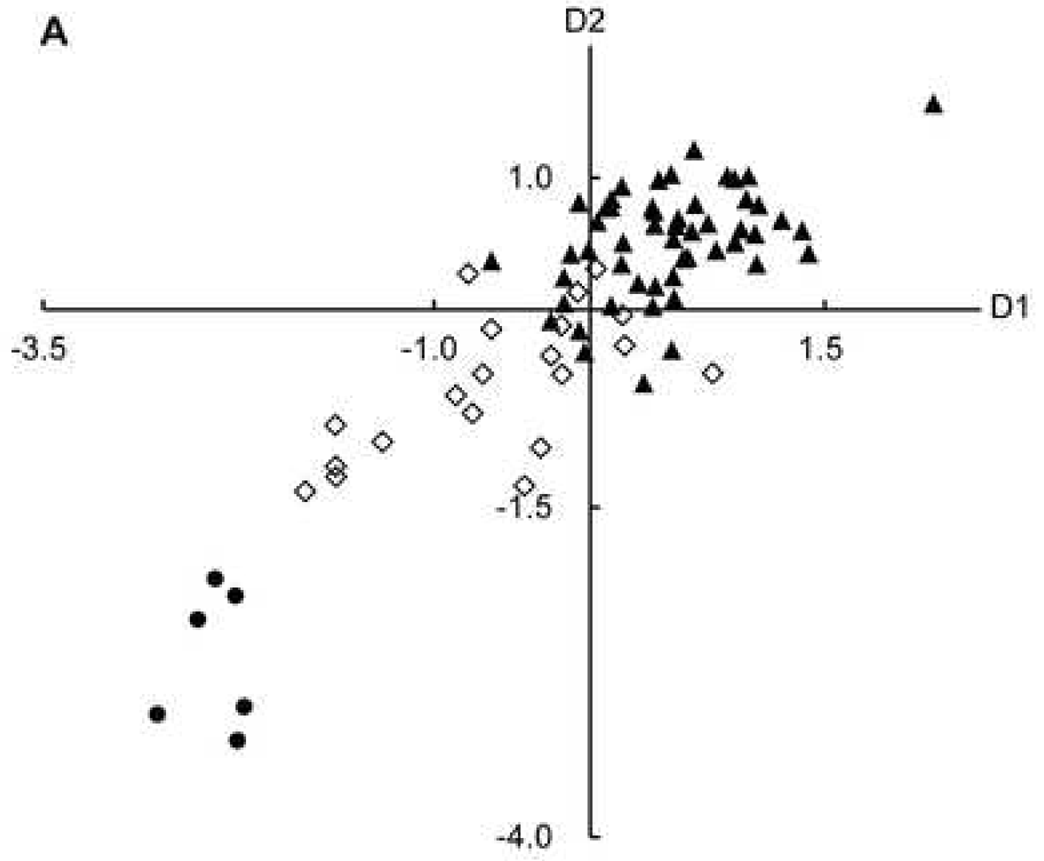

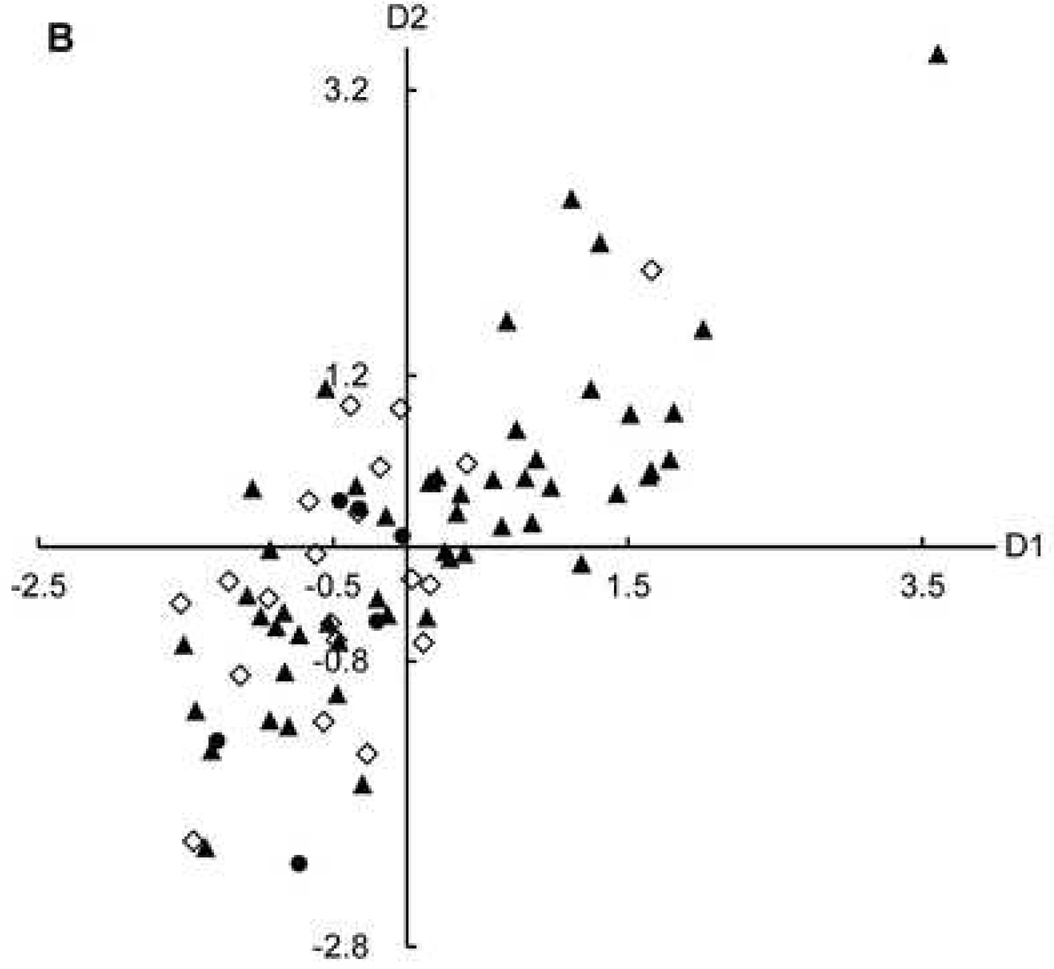

Click here to access/download;Figure;Fig_4.tif

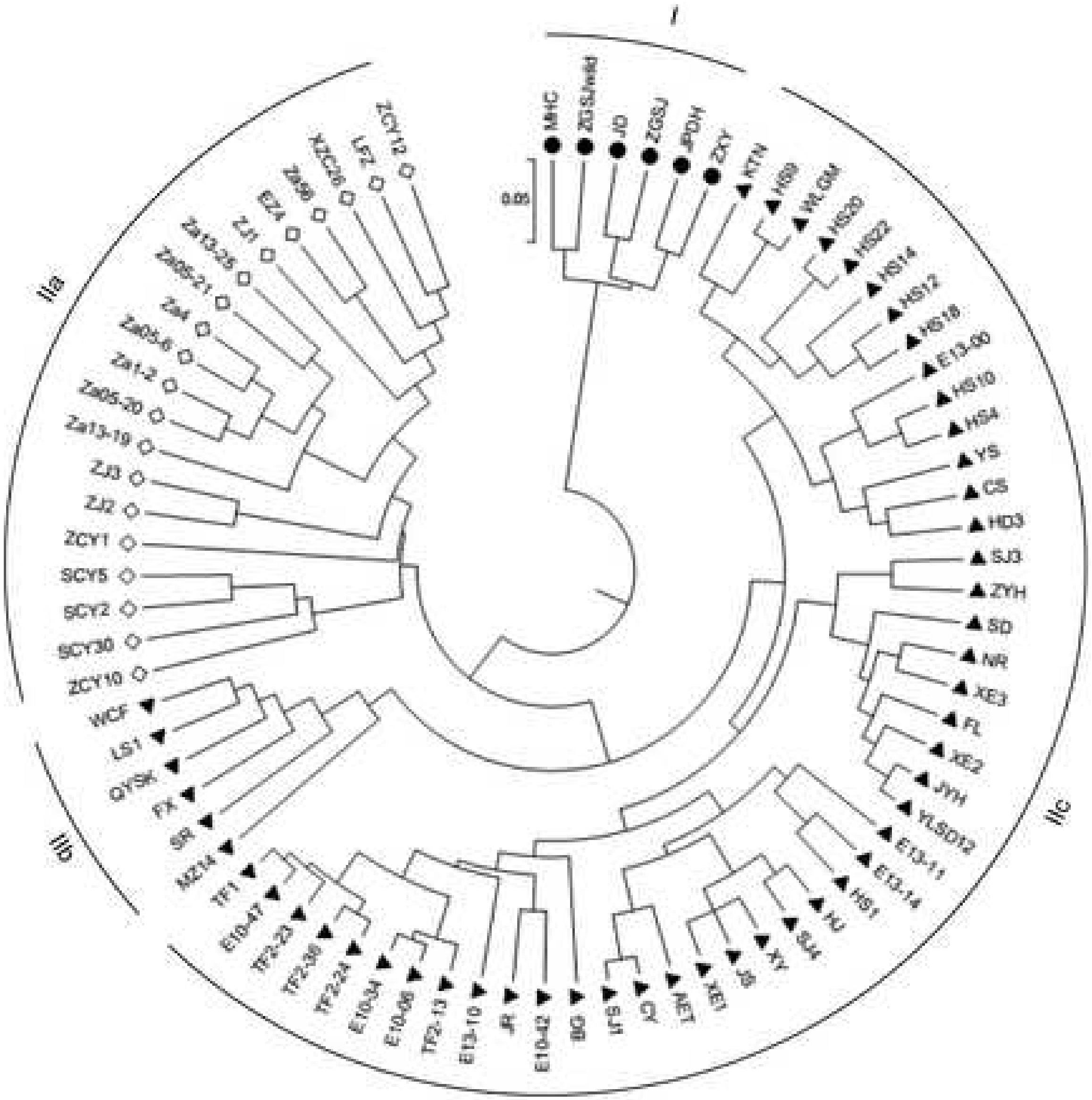

Click here to access/download
Supporting Information - Compressed/ZIP File Archive
Supporting Information.zip

Diversity in sea buckthorn (*Hippophae rhamnoides* L.) accessions with different origins based on morphological characteristics, oil traits, and microsatellite markers

He Li[1,2], Chengjiang Ruan[2]*, Jian Ding[2], Jingbin Li[2], Li Wang[2], Xingjun Tian[1,3]*

[1] School of Life Science, Nanjing University, Nanjing, P.R. China

[2] Key Laboratory of Biotechnology and Bioresources Utilization, Dalian Minzu University, Dalian, P.R. China

[3] Co-Innovation Center for Sustainable Forestry in Southern China, Nanjing Forestry University, Nanjing, P.R. China

* Corresponding authors

E-mail: ruan@dlnu.edu.cn (CR); tianxj@nju.edu.cn (XT)

Abstract

Sea buckthorn (*Hippophae rhamnoides*) is an ecologically and economically important species. Here, we assessed the diversity of 78 accessions cultivated in northern China using 8 agronomic characteristics, oil traits (including oil content and fatty acid composition) in seeds and pulp, and SSR markers at 23 loci. The 78 accessions included 52 from ssp. *mongolica*, 6 from ssp. *sinensis*, and 20 hybrids. To assess the phenotypic diversity of these accessions, 8 agronomic fruit traits were recorded and analyzed using principal component analysis (PCA). The first two PCs accounted for approximately 78% of the variation among accessions. The oil contents were higher in pulp (3.46-38.56%) than in seeds (3.88-8.82%), especially in ssp. *mongolica* accessions. The polyunsaturated fatty acids (PUFA) ratio was slightly lower in seed oil of hybrids (76.06%) than in ssp. *mongolica* (77.66%) and higher than in ssp. *sinensis* (72.22%). The monounsaturated fatty acids (MUFA) ratio of pulp oil of ssp. *sinensis* (57.00%) was highest, and that of ssp. *mongolica* (51.00%) was approximately equal to the ratio in the hybrids (51.20%). Using canonical correspondence analysis (CCA), we examined the correlation between agronomic traits and oil characteristics in pulp and seeds, respectively. Oil traits in pulp from different origins were correlated with morphological groupings (r = 0.8725, p = 0.0000). To assess the genotypic diversity, 23 SSR markers (including 17 loci previously reported) were used among the 78 accessions with 69 polymorphic amplified fragments obtained and an average PIC value of 0.2845. All accessions were classified into two groups based on the UPGMA method. The accessions of ssp. *sinensis* and ssp. *mongolica* were genetically distant.

The hybrid accessions were close to ssp. *mongolica* accessions. The 8 agronomic traits, oil characters in seed and pulp oils, and 23 SSR markers successfully distinguished the 78 accessions. These results will be valuable for cultivar identification and genetic diversity analysis in cultivated sea buckthorn.

Introduction

Sea buckthorn (*Hippophae rhamnoides* L.) is a winter hardy shrub that is naturally distributed throughout Asia and Europe. It is an economically valuable species, divided into eight subspecies. Of them, the ssp. *sinensis* and *mongolica* mainly distributed in Asia where they are abundant and commercially cultivated [1−2]. The fruits of sea buckthorn are rich in a variety of phytochemicals with physiological properties, such as lipids, carotenoids, ascorbic acid, tocopherols, and flavonoids [3−5]. The main applications for the fruits include food, cosmetics, and pharmaceutical products [6−7]. One of the most requested products for therapeutic practices is sea buckthorn oil, which is extracted from both seeds and pulp. The applications of sea buckthorn oil include healing of the skin, mucosa, and immune systems, especially in cancer and cardiovascular disease therapy [8−9].

Two important parameters in analyzing sea buckthorn oil quality are oil content and fatty acid composition (referred to here as ‘oil traits’ for simplicity). Sea buckthorn seed and pulp oils are considered the most valuable products of the berries with a unique fatty acid (FA) composition [10]. The seed oil contains omega-3 (α-linolenic acid) and omega-6 (linoleic acid) FAs, and the pulp oil is characterized by a high concentration

of FAs from the omega-7 group (e.g., palmitoleic acid). The seed oil is rich in unsaturated fatty acids (commonly 30-40% linoleic acid and 20-35% linolenic acid) [10]. The soft parts (pulp and peel) of the berries have a FA composition that differs from the seeds that is characterized by a high level of palmitoleic acid (16–54%), which is very uncommon in plants. The oil traits of sea buckthorn berries varies greatly according to their origin, based on the climatic and geological conditions of the growing areas [11].

Sea buckthorn adapts well to extreme conditions, including drought, salinity, alkalinity, and temperatures [12]. The vigorous vegetative reproduction and the strong, complex root system with nitrogen-fixing nodules make it an optimal pioneer plant for soil and water conservation. For these reasons, sea buckthorn was cultivated widely in arid and semiarid areas of China [13]. Due to small berries and thorns of native cultivars (ssp. *sinensis*), which have little economic value, the breeding of sea buckthorn has undergone different stages of development in China, such as introduction, domestication, seedling selection and artificial hybridization for elite accessions. The cultivars of ssp. *mongolica* (introduced from Russia and Mongolia), ssp. *sinensis* (China origin) and hybrids (ssp. *mongolica* × ssp. *sinensis*) are abundant in northern China [14]. However, as a perennial woody plant, traditional cross breeding that takes a long time and has low efficiency cannot meet the needs of modern production in sea buckthorn. It is essential for economic production to utilize molecular marker-assisted breeding (MAB) in sea buckthorn, especially to breed those accessions associated with desirable oil traits. An essential step in this process is the genetic analysis of sea

buckthorn germplasm. At present, molecular markers are mainly used for the analysis of genetic diversity, the taxonomic and geographic origin of cultivars, sex determination and population genetic structure in sea buckthorn [14−16]. SSR (simple sequence repeat, microsatellite) markers, with 1- to 6-bp DNA regions repeated in tandem, have been used in these analysis for their advantages of codominance, random distribution throughout the genome, easy detection, and high polymorphism and reproducibility [17]. Currently, an increasing number of microsatellite markers are being developed in sea buckthorn using high-throughput sequencing techniques for transcriptome datasets (RNA-Seq), which have become valuable resources for SSR discovery [14, 18].

The diversity analysis helps understand the relationships between germplasm characters and genotype will improve the sea buckthorn germplasm to achieve higher production with higher quality for the important traits were correlated with the promising germplasm [19].

In present study, 78 accessions of sea buckthorn with variation of fruit traits were selected as materials. The aim of this study is to report the phenotypic characteristics and oil traits in pulp and seeds, and genetic diversity of the 78 sea buckthorn accessions in northern China, providing the identification foundation for MAB in sea buckthorn.

Materials and methods

Plant materials

Berries and leaves of 78 sea buckthorn accessions originated from ssp. *mongolica* (52 accessions), ssp. *sinensis* (6 accessions) and hybrids (ssp. *mongolica* × ssp. *sinensis*, 20

accessions) were collected from the end of July to mid-September in 2015. These samples are from 235 individuals (2−5 ramet plants each accession) in different growth sites. Table 1 summarizes information on the plant materials. Three research institutes located in northern China, the Institute of Selection and Breeding of *Hippophae* (42°26′N, 121°28′E; 380 m) in Fuxin, the Research Institute of Berry (47°14′N, 127°06′E; 202 m) in Suiling and the Jiuchenggong Breeding Base of Sea Buckthorn (39°40′N, 110°09′E; 1400 m) in Dongsheng, provided 76 accessions of sea buckthorn samples of all (Fig 1, S1 and S2 Table). The other two accessions, Quyisike and Zhongguoshaji*wild*, were harvested from cultivated fields in Qinghe (46°40′N, 90°22′E; 1218 m) and Datong (36°53′N, 101°35′E; 2800 m) (Fig 1 and S1 Table). These areas with various geographical and climatic conditions ranged between latitudes 36°53′N–47°14′N, longitudes 90°22′E–127°06′E, and altitudes 202–2800 m (S3 Table).

The young leaves of each plant were kept at −80 °C for use. The berries of each accession were pooled and frozen as quickly as possible at −20 °C. When all plant materials were harvested, the berries were transferred to –50 °C for storage until analysis.

Fig 1. Five cultivated lands of the 78 sea buckthorn accessions used in this study.

Table 1. Accessions of sea buckthorn usedc for the study.

No.	Accession name	Abbrev.[a]	Trees (no.)[b]	Collection site	ssp.[c]	No.	Accession name	Abbrev.[a]	Trees (no.)[b]	Collection site	ssp.[c]
1	Zhuangyuanhuang	ZYH	5	Fuxin	M	40	E13-10	E13-10	3	Suiling	M
2	Wucifeng	WCF	5	Fuxin	M	41	E13-11	E13-11	3	Suiling	M

3	Liusha-1	LS1	5	Fuxin	M	42	E13-14	E13-14	3	Suiling	M
4	Siberia rumianes	SR	4	Fuxin	M	43	HS-1	HS1	3	Suiling	M
5	Fangxiang	FX	2	Fuxin	M	44	HS-4	HS4	3	Suiling	M
6	Yalishanda-12	YLSD12	4	Fuxin	M	45	HS-9	HS9	3	Suiling	M
7	Jiuyuehuang	JYH	2	Fuxin	M	46	HS-10	HS10	3	Suiling	M
8	Nanren	NR	2	Fuxin	M	47	HS-12	HS12	3	Suiling	M
9	Botanical garden	BG	2	Fuxin	M	48	HS-14	HS14	3	Suiling	M
10	Zajiao-1	ZJ1	2	Fuxin	H	49	HS-18	HS18	3	Suiling	M
11	Zajiao-2	ZJ2	2	Fuxin	H	50	HS-20	HS20	3	Suiling	M
12	Zajiao-3	ZJ3	2	Fuxin	H	51	HS-22	HS22	3	Suiling	M
13	MZ-14	MZ14	3	Suiling	M	52	Xin’e-1	XE1	3	Suiling	M
14	Shoudu	SD	3	Suiling	M	53	Xin’e-2	XE2	3	Suiling	M
15	Fenlan	FL	3	Suiling	M	54	Xin’e-3	XE3	3	Suiling	M
16	Aertai	AET	3	Suiling	M	55	Zhongguoshaji	ZGSJ	3	Suiling	S
17	Chengse	CS	3	Suiling	M	56	EZ-4	EZ4	3	Suiling	H
18	Chuyi	CY	3	Suiling	M	57	Za-56	Za56	3	Suiling	H
19	Hunjin	HJ	3	Suiling	M	58	Za1-2	Za1-2	3	Suiling	H
20	Jinse	JS	3	Suiling	M	59	Za05-6	Za05-6	3	Suiling	H
21	Juren	JR	3	Suiling	M	60	Za05-20	Za05-20	3	Suiling	H
22	Xiangyang	XY	3	Suiling	M	61	Za05-21	Za05-21	3	Suiling	H
23	Yousheng	YS	3	Suiling	M	62	Za4	Za4	3	Suiling	H
24	Katuni	KTN	3	Suiling	M	63	Za13-19	Za13-19	3	Suiling	H
25	Wulangemu	WLGM	3	Suiling	M	64	Za13-25	Za13-25	3	Suiling	H
26	TF1	TF1	3	Suiling	M	65	Juda	JD	3	Dongsheng	S
27	TF2-13	TF2-13	3	Suiling	M	66	Jianpingdahuang	JPDH	3	Dongsheng	S
28	TF2-23	TF2-23	3	Suiling	M	67	Manhanci	MHC	3	Dongsheng	S
29	TF2-24	TF2-24	3	Suiling	M	68	Zhongxiongyou	ZXY	3	Dongsheng	S
30	TF2-36	TF2- 36	3	Suiling	M	69	Liaofuza	LFZ	3	Dongsheng	H
31	Suiji-1	SJ1	3	Suiling	M	70	Zaciyou-1	ZCY1	3	Dongsheng	H
32	Suiji-3	SJ3	3	Suiling	M	71	Zaciyou-10	ZCY10	3	Dongsheng	H

33	Suiji-4	SJ4	3	Suiling	M	72	Zaciyou-12	ZCY12	3	Dongsheng	H
34	HD-3	HD3	3	Suiling	M	73	Xinzaci-26	XZC26	3	Dongsheng	H
35	E10-06	E10-06	3	Suiling	M	74	Shiciyou-2	SCY2	3	Dongsheng	H
36	E10-34	E10-34	3	Suiling	M	75	Shiciyou-5	SCY5	3	Dongsheng	H
37	E10-42	E10-42	3	Suiling	M	76	Shiciyou-30	SCY30	3	Dongsheng	H
38	E10-47	E10-47	3	Suiling	M	77	Zhongguoshaji^{wild}	ZGSJ^{wild}	3	Datong	S
39	E13-00	E13-00	3	Suiling	M	78	Qiuyisike	QYSK	3	Qinghe	M

[a] Abbrev., abbreviation.

[b] Trees (no.) = number of trees.

[c] ssp., subspecies; M, ssp. *mongolica*; S, ssp. *sinensis*; H, hybrid (ssp. *mongolica* ♀ × ssp. *sinensis* ♂).

Morphological characteristics of fruit

Hundred berry weight (HBW) was the weight of 100 fresh berries after they were picked from bushes. Hundred seed weight (HSW) was the weight of 100 seeds after air drying at room temperature (25 °C) for 2 weeks [20]. There were three biological replicates for each measurement. The transverse and longitudinal diameters of berries (BTD and BLD) and the length, width and thickness of seeds (SL, SW and ST) were measured by micrometer calipers with over 20 measurements for each, on average. The berry shape indices (BSI) were estimated by the ratio of BLD to BTD. The data of minimum (Min), maximum (Max), mean ± SD (standard deviation), and coefficient of variation (CV%) were reported.

Oil extraction and FA analysis in seeds and pulp

The methods of lipid extraction, transesterification (methylation) and purification of

methyl esters of the lipid extracts were described by Yang and Kallio [11]. Briefly, the seeds and pulp isolated from freeze-dried berries and lipids from the samples were extracted with chloroform/methanol (2:1, v/v) with mechanical homogenization of the tissues. The purified oils were filtered before the solvent was removed on a rotary evaporator. The lipids were weighed, and the oil contents (percentages) in seeds and pulp were calculated. Three biological replicates were taken for analysis. Lipids were stored in chloroform at −20 °C until analysis.

The oil (10 mg) was transesterified by sodium methoxide catalysis [11, 21]. It was dissolved in sodium-dried diethyl ether (1ml) and methyl acetate (20 μl). Then 1 M sodium methoxide in dry methanol (20 μl) was added, and the solution was agitated briefly and set still for 5 min at room temperature. The reaction is stopped by adding a saturated solution of oxalic acid in diethyl ether (30 μl) with brief agitation. The mixture is centrifuged at 1500 g for 2 min and the supernatant was dried in a gentle stream of nitrogen. Fresh hexane (1 ml) was added and the solution was filtered with microporous filtering films (0.22 μm) for analysis.

FAMEs were analyzed with a gas chromatography-tandem mass spectrometry (GC/MS/MS) system (model AxION® iQT™, PekinElmer, Shelton, CT, USA). Chromatographic separation was achieved using a DB-23 capillary column (60 m × 0.25 mm × 0.25μm; Agilent Technologies, Santa Clara, CA, USA) with the following temperature program: initial temperature 50 °C, hold for 1 min, heated to 175 °C at 25 °C/min, then heated to 215 °C at 3 °C/min and hold for 10 min, heated to 230 °C at 3 °C/min and hold for 5 min. The inlet was operated in split mode (1:20) at a

temperature of 250 °C with helium as the carrier gas at constant flow of 1.0 mL/min. The transfer line temperature was 215 °C, and the MS ion source was set to 230 °C. MS detection was carried out in electron impact (EI) ionization mode, scanning all masses from 45–400 amu. FAME components were identified based on mass spectral comparison with an external standard (Supelco 37 Component FAME Mix, Sigma-Aldrich, St. Louis, MO, USA) and previous studies [10−11]. The main fatty acid composition was expressed as a weight percentage of the total fatty acids from three replicates. The data of minimum, maximum, mean ± SD, and coefficient of variation were reported.

Statistical analysis

The data analysis for morphological traits and oil characteristics were performed with SPSS® 24.0 (IBM®). The following parameters were evaluated: mean, minimum value, maximum value, standard deviation (SD) and coefficient of variation (CV%). One-way ANOVA was used in the comparison of all traits among subsp. of *sinensis*, subsp. of *mongolica* and hybrids. Pearson correlation coefficients were calculated to analyze the relationship between pairs of 8 agronomic traits. Principal component analysis (PCA) was used to determine relationships among the accessions. In addition, a canonical correspondence analysis (CCA) was applied to the data between morphological characteristics and oil traits in different tissues (seeds and pulp).

DNA extraction and SSR analysis

Total genomic DNA was extracted from young leaves using the TaKaRa MiniBEST Plant Genomic DNA Extraction Kit (TaKaRa, Beijing, China) based on the manufacturer's protocol. Purity and quantity of extracted DNA were evaluated by gel electrophoresis and NanoDrop 2000 Spectrophotometer (Thermo Fisher Scientific, Waltham, MA, USA). Twenty-three polymorphic microsatellite loci (SSR) developed using RNA-Seq were evaluated and loci SB1-SB17 were previously reported [14] (S4 Table). PCR amplification was performed in 20 μL volumes containing 40 ng of DNA template, 1× PCR buffer, 1.5 mM $MgCL_2$, 0.15 mM of each dNTP (Takara, Dalian, China), 1.5 U of Taq polymerase (Takara, Dalian, China) and 0.5 μM of each primer. The PCR conditions included an initial denaturation at 94 °C for 2 min, 35 cycles of 30 s at 94 °C for denaturation, 30 s at 54–60 °C for annealing and 45 s at 72 °C for extension, with a final extension 7 min at 72 °C using a C1000 Touch™ Thermal Cycler (Bio-Rad, Berkeley, CA, USA). PCR products were electrophoresed on 8% non-denaturing polyacrylamide gels using a SE 600 Ruby Standard Dual Cooled Vertical Unit (GE Healthcare Life Sciences, Pittsburgh, PA, USA) and visualized by silver staining.

The microsatellites were scored as codominant markers for genetic diversity analysis. The number of alleles (Na), effective number of alleles (Ne), observed and expected heterozygosity (Ho and He), Shannon's information index (Is) and polymorphic information content (PIC) for each of the genic SSR markers were calculated using GenAlEx 6.5 [22−23] and PowerMarker version 3.25 [24] software packages. A genetic similarity matrix based on the proportion of shared alleles was

generated, and a UPGMA tree was constructed using PowerMarker. The dendrogram was displayed using MEGA 6 software [25] to reveal genetic relationships between the 78 sea buckthorn accessions.

Results

Morphological characterization of berries and seeds

Descriptive statistics analysis of 8 agronomic fruit traits for the 78 sea buckthorn accessions is shown in Table 2, S5 and S6 Table. Relatively high CV values were observed in HBW, BLD, and HSW (> 20%). The highest coefficient of variation was observed in HBW (39.12%), which varied from 8.52 to 69.74 g. Analysis of variance (ANOVA, $p < 0.05$) showed that HBW of ssp. *mongolica* berries was 47.69 ± 11.03 g, which was much higher than ssp. *sinensis* berries (10.73 ± 1.54 g) and hybrids (31.44 ± 13.84 g). In hybrids, the HBW values were high in EZ4, Za56, Za1-2, Za05-6 and Za05-21(> 45 g), which were approximately the size of ssp. *mongolica* berries on average (S6 Table). BTD varied from 5.54 to 10.80 mm and BLD varied from 4.83 to 14.25 mm. In addition, BLD of berries from ssp. *mongolica* was higher than BTD, which was the opposite in berries of ssp. *sinensis*. According to BSI values, the berry shapes of the three groups were significantly different ($p = 0.000$): oblong berries of ssp. *mongolica* (1.35 ± 0.20), oblate for those of ssp. *sinensis* (0.90 ± 0.05) and circular for those of hybrids (1.08 ± 0.11). HSW varied from 0.61 to 2.19 g with an average of 1.45 g. Similar to HBW, there were significant differences for HSW among seeds from ssp. *mongolica*, ssp. *sinensis*, and hybrids ($p = 0.000$). SL varied from 2.00 to 3.49 mm

and SW varied from 2.98 to 7.43 mm. ST varied from 1.54 to 2.73 mm with an average of 1.93 mm. Overall, the agronomic characters of seeds (HSW, SL, SW, and ST) showed relatively low coefficients of variation, ranging between 11.50–24.33%; however, the berries (HBW, BTD, BLD, and BSI) had high coefficients of variation.

Table 2. Fruit traits of sea buckthorn berries of two different subspecies and hybrid accessions[a].

**Trait name**	**Abbrev.[b]**	**ssp. *mongolica***	**ssp. *sinensis***	**Hybrid**
Hundred berry weight (g)	HBW (g)	47.69 ±11.03a	10.73 ± 1.54c	31.44 ±13.84b
Berry transverse diameter (mm)	BTD (mm)	8.17 ± 0.99a	5.84 ± 0.23b	7.61 ± 1.24a
Berry longitudinal diameter (mm)	BLD (mm)	10.90 ± 1.48a	5.20 ± 0.19c	8.15 ± 1.18b
Berry shape index (%)	BSI (%)	1.35 ± 0.20	0.90 ± 0.05	1.08 ± 0.11
Hundred seed weight (g)	HSW (g)	1.60 ± 0.28a	0.79 ± 0.23c	1.28 ± 0.25b
Seed length (mm)	SL (mm)	5.91 ± 0.68a	3.31 ± 0.27c	4.64 ± 0.56b
Seed width (mm)	SW (mm)	2.76 ± 0.27a	2.18 ± 0.18c	2.52 ± 0.22b
Seed thickness (mm)	ST (mm)	1.98 ±0.18a	1.67 ± 0.16 b	1.86 ± 0.26a

[a] Values with different lower case letters (a–c) are significantly different at $p < 0.05$.

[b] Abbrev., Abbreviation.

In previous mutilocation trials in Suiling (47°14′N, 127°06′E; 202 m) and Dengkou (40°43′N, 106°30′E; 1053m, Inner Mongolia), the fruit characteristics of 11 large

berry accessions (AET, CS, CY, HJ, JS, JR, XY, YS, KTN, WLGM and SJ1) were comparatively analyzed (S7 Table). The HBWs of them in Suiling (38.33–67.59 g) were higher than those in Dengkou (32.87–63.85). For all the introduced cultivars, the HBWs in two experimental fields were lower than those in their country of origin, Russia. The phenotypic characteristics of sea buckthorn berries showed differences due to their origins, berry parts analyzed, climate and growing conditions. In this study, the 78 accessions were selected for their adaptabilities to growth sites.

PCA was performed using fruit characteristics (Fig 2). The first two principal components explained 78.11% of the total morphological variance. The first principle component (PC) accounted for 41.74% of the variance. It was associated with BTD, HBW, ST, HSW, and SW in descending order. Therefore, these traits were important attributes for the classification of sea buckthorn accessions. The second PC accounted for 36.37%, which is correlated with BSI, SL, and BLD in descending order. The plot shows the distribution of 78 sea buckthorn accessions on PC1 and PC2 (Fig 2). The ssp. *mongol*ica accessions with bigger berries tended to cluster together, mainly positive on PC2. Six accessions of ssp. *sinensis* with the smallest berries were negative on both PC1 and PC2. The hybrids were largely distributed between the above two groups. Some hybrids (including ZCY1, ZCY10, ZCY12, XZC26, SCY2, and SCY5) were close to the accessions from ssp. *sinensis*.

Fig 2. Two-dimensional scatter plot for the first two principal components (PC1 and PC2) based on the agronomic fruit characteristics of 78 sea buckthorn accessions. Numbers associated with

symbols are the variety codes as listed in Table 1. ▲ = ssp. *mongolica*; ● = ssp. *sinensis*; ◇ = hybrid.

Oil characterization in seeds and seedless parts

The oil characteristics of seeds and seedless parts (pulp and peel) among the 78 accessions are summarized in Tables 3 and Table 4. One special feature of sea buckthorn fruit was the high oil content in the pulp and peel (20.41%), in contrast to oil in seeds (8.82%). A higher coefficient of variation was observed in pulp oil content (42.72%) and varied over a wide range, from 3.46 to 38.56%. The pulp fraction of berries of ssp. *mongolica* had the highest oil content (24.68%) based on dry weight. The lowest pulp oil content (7.10%) on average was found in the berries of ssp. *sinensis*. In hybrids, the berries of ZJ2 contained 27.22% pulp oil, which slightly exceeded that of ssp. *mongolica* on average (S6 Table). Seed oil content varied from 3.88 to 12.75% with an average of 8.82%. The seeds of ssp. *mongolica* had the highest oil contents with an average of 9.46%, and those of the other two groups did not differ significantly.

Table 3. Oil characteristics of pulp and seeds of 78 sea buckthorn accessions.

Character	Pulp				Seed			
	**Min[a]**	**Max[b]**	**Mean ± SD[c]**	**CV[d] (%)**	**Min[a]**	**Max[b]**	**Mean ± SD[c]**	**CV[d](%)**
oil content	3.46	38.56	20.41 ± 8.72	42.72	3.88	12.75	8.82 ± 1.86	21.08
16:0	24.52	53.08	36.26 ± 4.83	13.32	3.84	11.77	6.55 ± 1.39	21.16
16:1n7	17.93	57.75	35.12 ± 7.64	21.76	tr[e]	tr[e]	tr[e]	
18:0	0.38	5.12	1.26 ± 0.70	55.58	1.41	4.58	2.16 ± 0.43	20.11

18:1n9	1.44	23.43	8.72 ± 4.72	54.13	3.05	25.95	13.25 ± 4.04	30.50
18:1n7	3.51	24.24	7.68 ± 4.09	53.28	0.45	2.38	1.20 ± 0.47	39.17
18:2n6	3.02	17.40	9.97 ± 3.18	31.91	34.22	52.75	42.17 ± 3.60	8.54
18:3n3	0.12	7.16	1.00 ± 1.03	102.83	21.37	47.16	34.67 ± 4.42	12.75

[a] Minimum value.

[b] Maximum value.

[c] Standard deviation.

[d] Coefficient of variation expressed in percentage.

[e] tr, trace (< 0.5%).

Table 4. **Oil content and fatty acid composition in seeds and the soft parts of sea buckthorn berries of different origins[a].**

	**Pulp oil**			**Seed oil**		
**Character**	**ssp. *mongolica***	**ssp. *sinensis***	**Hybrid**	**ssp. *mongolica***	**ssp. *sinensis***	**Hybrid**
oil content	24.68 ± 6.79 a	7.10 ± 3.28c	13.34 ± 4.85b	9.46 ± 1.56a	6.70 ± 1.32b	7.78 ±1.84b
16:0	37.68 ± 4.64a	29.39 ± 3.71b	34.62 ± 3.14a	6.52 ± 1.16	7.41 ± 1.55	6.38 ± 1.82
16:1n7	37.43 ±7.09a	23.65 ± 4.16b	32.55 ± 5.84a	tr[b]	tr[b]	tr[b]
18:0	1.08 ±0.69b	1.73 ± 0.64a	1.59 ± 0.57ab	2.13 ± 0.29	2.19 ± 0.44	2.23 ± 0.69
18:1n9	7.56 ±3.97b	16.67 ± 6.84a	9.33 ± 3.40b	12.62 ± 3.75b	16.37 ± 3.77a	13.96 ± 4.46ab
18:1n7	6.01 ±1.79c	16.68 ± 6.20a	9.32 ± 3.63b	1.07 ± 0.37b	1.80 ± 0.39a	1.37 ± 0.55b
18:2n6	9.55 ±2.76ab	8.34 ± 5.54b	11.53 ± 2.92a	42.10 ± 3.08	40.44 ± 4.06	42.87 ± 4.62
18:3n3	0.69 ±0.41b	3.54 ± 2.09a	1.07 ± 0.64b	35.56 ± 4.13a	31.78 ± 2.91b	33.20 ± 4.89 ab
MUFA	51.00 ±5.38b	57.00 ± 9.46a	51.20 ± 3.52b	13.69 ± 3.93b	18.18 ± 4.09a	15.33 ± 4.90ab
PUFA	10.24 ±2.98	11.89 ± 7.54	12.60 ±3.37	77.66 ± 4.31a	72.22 ±5.54b	76.06 ± 6.23ab

[a] Values with different lowercase letters (a–c) are significantly different at $p < 0.05$.

[b] tr, trace (< 0.5%).

For sea buckthorn, the FA composition in seed oil differed significantly from that in pulp oil. The proportions of linoleic (18:2n6), α-linolenic (18:3n3), oleic (18:1n9), palmitic (16:0), stearic (18:0) and vaccenic (18:1n7) acids were found from high to low in seed oil of most accessions (Table 4). Linoleic acid varied from 34.22 to 52.75% with an average of 42.17%. The proportion of α-linolenic acid varied from 21.37 to 47.16% with an average of 34.67%. High CV values were observed in oleic (30.50%) and vaccenic (39.17%) acids. Furthermore, the level of palmitoleic acid (16:1n7, < 0.5%) was extremely low in seed oil. The FA composition of sea buckthorn seeds were similar among berries of the two different subspecies and hybrid accessions. Small variations were found in the proportion of linoleic acid in seed oil (40.44 – 42.87%). Its proportion in hybrids were slightly higher than in ssp. *mongolica* (42.87% vs 42.10%), and had the highest value of the samples from the two different subspecies and hybrid accessions. α-Linolenic acid showed a little variation with a bigger proportion in ssp. *mongolica* than in ssp. *sinensis* (35.56% vs 31.78%). A higher proportion of palmitic (7.41% vs 6.38%) and oleic (16.37% vs 13.96%) acids and a lower proportion of stearic acid (2.19% vs 2.23%) were discovered between the accessions of ssp. *sinensis* and hybrids. The polyunsaturated fatty acids (PUFA) ratio in hybrids (76.06%) was slightly lower than it was in ssp. *mongolica* (77.66%) and higher than it was in ssp. *sinensis* (72.22%). Some hybrids (including ZJ1, Za1-2, Za13-25, Za05-6, LFZ, and ZCY12) contained a high proportion of PUFA (> 80%) in seed oil, which was more than the average level of ssp. *mongolica* accessions (S6 Table).

In pulp oil, the dominant FAs were palmitoleic, palmitic, linoleic, oleic, and vaccenic acids (Table 3). Major differences were observed in the proportion of palmitoleic (17.93-57.75%), oleic (1.44-23.43%) and vaccenic (3.51-24.24%) acids. The special feature of pulp oil is high proportions (> 35%) of palmitoleic and palmitic acids. Compared to ssp. *sinensis*, ssp. *mongolica* contained a higher proportion of palmitoleic and palmitic acids in the berry pulp ($p < 0.05$) (Table 4). In particular, the proportions of oleic and vaccenic acids were highest in ssp. *sinensis*, much higher than those in ssp. *mongolica* and hybrid accessions. The relative levels of α-linolenic and stearic acids in pulp of ssp. *sinensis* were higher than ssp. *mongolica* ($p < 0.05$) (Table 4). For hybrids, the proportions of most fatty acids were between ssp. *mongolica* and ssp. *sinensis* accessions, except for linoleic acid. Similar to the results in seed oils, the hybrids had the highest proportions of linoleic acid (11.53%) and PUFA (12.60%). The monounsaturated fatty acids (MUFA) ratio in pulp oil of ssp. *sinensis* (57.00%) was highest and that of ssp. *mongolica* (51.00%) was almost equal to hybrids (51.20%). In hybrids, the pulp oil of SCY2 contained 39.16% palmitoleic acid, and the content of MUFA was 60.77%, which was higher than it was in ssp. *sinensis* (S6 Table).

Correlations among the agronomic traits and oil characters

Canonical analyses allow direct comparisons of two data matrices. All sea buckthorn accessions were represented in a two-dimensional space using CCA between phenotypic traits and oil characteristics (Fig 3). For berries of the two different subspecies and hybrid accessions, phenotypic characters (BLD, HBW, BSI, and BTD)

of berries and oil traits in pulp showed close correlation (r = 0.8725, p = 0.0000). Based on CCA, accessions of ssp. *mongolica* were clustered on the upper side (mainly positive on D1 and D2), those of ssp. *sinensis* on the other, and the hybrids in the middle in Fig 3A. The positioning of samples in the first dimension was mostly related to differences in their berry characteristics that were primarily provided by a marker of BLD. The second dimension indicated differences in oil contents and FA compositions of pulp oil among sea buckthorn accessions. Differences between pulp oil traits were primarily related to percentages of oil content, 16:0 and 16:1n7, which were highest in ssp. *mongolica*, followed by hybrids, and lowest in ssp. *sinensis*. For seeds of 78 accessions, phenotypic characters (SL, SW, ST, and HSW) and seed oil traits were correlated (r = 0.7482, p = 0.0000). The positioning of samples was staggered (Fig 3B), which reflected that all seed samples had relatively little variation among phenotypic traits and oil characteristics. These results verified the previous analysis (Table 2 and Table 3).

Fig 3. Canonical correspondence analysis of phenotypic traits (A. berry; B. seed) and oil characteristics (A. pulp oil; B. seed oil) of sea buckthorn germplasms. D1, Dimension 1; D2, Dimension 2. ▲ = ssp. *mongolica*; ● = ssp. *sinensis*; ◇ = hybrid.

SSR diversity

Twenty pairs of RNA-Seq SSR primers with good amplification and band stability were used in 78 accessions of sea buckthorn. A total of 69 bands were amplified using the 23

primer pairs, of which 59 were polymorphic, accounting for 85.51% of all. The number of amplified bands per locus ranged from 2 to 5, averaging 3, and the number of effective alleles (Ne) ranged from 1.0392 to 3.1049, averaging 1.6602 (Table 6). SB2, SB3, SB5, SB6, SB8, SB13, SB16 and SB23 were informative SSR loci, each revealing more than four effective alleles distributed among all of the accessions. Compared with the observed allele number (Na), the number of effective alleles and their average values were lower, which was caused by the uneven distribution of gene frequencies in SSR loci. In genetic diversity analysis, observed heterozygosity (Ho) ranged from 0.0385 to 0.7949, with an average of 0.2965; expected heterozygosity (He) ranged from 0.0377 to 0.6779, with an average of 0.3291, and the Shannon index (Is) ranged from 0.0950 to 1.2152, with an average of 0.5681. The value of polymorphism information content (PIC), regarded as discriminating power, varied from 0.0370 to 0.6174, with an average of 0.2845. Loci SB06 (PIC = 0.6174) and SB08 (PIC = 0.5820) showed higher effectiveness because of their high informativity, which could be used to construct the fingerprint map of sea buckthorn germplasm. The characteristics of these 23 loci in genetic diversity analysis of sea buckthorn germplasm are shown in Table 5.

Table 5. Characterization of 20 polymorphic SSR markers in the 78 sea buckthorn accessions.

Loci code	Na	Ne	Ho	He	PIC	Is
SB1	3	1.2745	0.2436	0.2154	0.2025	0.3956

SB2	4	1.1382	0.1282	0.1214	0.1166	0.2791
SB3	4	2.2372	0.4615	0.5530	0.4627	0.9090
SB4	2	1.5006	0.2692	0.3336	0.2779	0.5160
SB5	4	2.1129	0.3333	0.5267	0.4735	0.9288
SB6	4	3.1049	0.7051	0.6779	0.6174	1.2152
SB7	2	1.0799	0.0769	0.0740	0.0712	0.1630
SB8	5	2.8490	0.3846	0.6490	0.5820	1.1890
SB9	2	1.1509	0.1410	0.1311	0.1225	0.2550
SB10	3	1.5350	0.2949	0.3485	0.3114	0.6253
SB11	2	1.9287	0.1667	0.4815	0.3656	0.6745
SB12	3	1.2430	0.2179	0.1955	0.1753	0.3687
SB13	4	2.1644	0.4231	0.5380	0.4392	0.8687
SB14	2	1.9987	0.3077	0.4997	0.3750	0.6928
SB15	2	1.0662	0.0641	0.0620	0.0601	0.1418
SB16	4	1.4567	0.1923	0.3135	0.2956	0.6427
SB17	2	1.4175	0.3590	0.2945	0.2512	0.4706
SB18	2	1.0392	0.0385	0.0377	0.0370	0.0950

SB19	3	1.0804	0.0641	0.0744	0.0724	0.1804
SB20	2	1.1803	0.1667	0.1528	0.1411	0.2868
SB21	3	1.9123	0.7308	0.4771	0.3802	0.7318
SB22	3	1.2905	0.2564	0.2251	0.2025	0.4084
SB23	4	2.4239	0.7949	0.5874	0.5102	1.0284

Na, observed number of alleles; Ne, effective number of alleles; Ho, observed heterozygosity; He, expected heterozygosity; PIC, polymorphism information content; Is, Shannon's information index.

Genetic relationships among sea buckthorn germplasm

The sea buckthorn germplasm in this study originated from ssp. *mongolica* (52 accessions), ssp. *sinensis* (6 accessions) and hybrids (20 accessions). Using 23 polymorphic SSR markers, the UPGMA dendrogram based on the proportion of shared alleles was constructed to assess the genetic relationships between the 78 accessions (Fig 4). The results showed that all the accessions could be divided into two groups (I and II). The accessions of ssp. *sinensis* (JD, ZGSJ, MHC, ZGSJ^{wild}, JPDH and ZXY) were clustered into group I. These accessions had closer relationships, despite great geographic differences. The second group was divided into 3 subgroups, namely, IIa, IIb, and IIc. The 20 hybrid accessions were all clustered into IIa. Subgroup IIb and IIc contained all the accessions of ssp. *mongolica* (introduced from Russia and Mongolia). Subgroup IIb included 6 accessions, namely WCF, LS1, QYSK, FX, SR, MZ14. The rest accessions of ssp. *mongolica* were clustered into IIc. Among them, KTN, WLGM,

HS4, HS9, HS10, HS12, HS14, HS18, HS20, HS22, WCF, FX and MZ14 composed one sub-subgroup. SJ3, ZYH, SD, NR, FL, XE2, XE3, JYH and YLSD12 showed close relationships. Other 23 accessions clustered into the third sub-subgroup. Overall, the relationship between ssp. *mongolica* and ssp. *sinensis* was relatively distant. The hybrids are close to ssp. *mongolica* which their female parents belonged to.

Fig. 4. UPGMA dendrogram of sea buckthorn germplasm based on SSR data (sample abbreviations described in Table 1). ▲ = ssp. *mongolica*; ● = ssp. *sinensis*; ◇ = hybrid.

Discussion

Morphological characteristics, biochemical traits, and microsatellite markers have been used for germplasm identification and genetic diversity analysis in many horticultural plants [26–27]. The diversity at morphological, biochemical, and molecular levels of 78 sea buckthorn accessions, composed of 52 from ssp. *mongolica*, 6 from ssp. *sinensis*, and 20 hybrids, were investigated.

The morphological characterization of plant materials with desired traits is an essential step for the effective use of germplasm [28]. Here, 8 important agronomic traits were measured among 78 sea buckthorn accessions, and a considerable amount of variation in morphological traits was found. The berry sizes of berries from the two different subspecies and hybrid accessions were significantly different according to the HBW value (p = 0.000). Compared to ssp. *sinensis* berries, ssp. *mongolica* berries were much bigger on average. The berry size of hybrid accessions were between the two

subspecies. In PC analysis, we plotted 2D plots with PC1 and PC2 scores of phenotypes (Fig 2). PC1 was mainly related with BTD and HBW, which explained the largest portion of the variance in 78 accessions. The distribution of 78 accessions on PC1 and PC2 was consistent with their agronomic characters (Fig 2). These results estimating morphological traits are valuable tools for identifying variation among plant germplasm [26].

For biochemical traits, oil content and FA composition in seeds and seedless parts were selected for their important roles in human health. The oil of sea buckthorn seems to be a good source of unsaturated fatty acids. The seed oil is rich in PUFA, including linoleic and α-linolenic acids. The proportion of PUFA did not differ significantly among berries from three origins, despite the differences in some morphological characteristics and in growth conditions. These results were consistent with the previous studies [10]. The results of the present study and previous investigations also suggested that the berries of ssp. *mongolica* were a good source of palmitic and palmitoleic acids in pulp oil and those of ssp. *sinensis* were a good source of oleic acid, both in seeds and pulp [29]. Although carefully selected for intersubspecies crosses, some hybrids displayed elite oil traits. For example, the proportion of MUFA in pulp of SCY2 and of PUFA in seeds of 6 accessions (including ZJ1, Za1-2, Za13-25, Za05-6, LFZ, and ZCY12) exceeded the average level of ssp. *mongolica* accessions, the subspecies of one of their parents belonged to. These results demonstrate the effectiveness of traditional cross breeding in the improvement of native accessions (ssp. *sinensis*), even though it is time-consuming and has low efficiency.

Previous studies found that berry size is a useful indicator of Vc, sugars and acids in population identification [19, 30]. The nutrients in the seedless fraction were more concentrated in the small berries of ssp. *sinensis* than in the large berries of ssp. *mongolica* [29]. In the present study, we analyzed the correlation between agronomic characteristics and oil traits at different levels (seed and pulp) by CCA. The results showed phenotypic characteristics (BLD, HBW, BSI, and BTD) of berries and oil traits in pulp were positively correlated (r = 0.8725, p = 0.0000). BLD, as a promising marker, provided the primary difference in CCA. Our results illustrated that berry size had different correlations with various biochemical characteristics in sea buckthorn.

Variation of phenotypic traits among germplasms may be attributed to differences in genetic backgrounds, geographical location, climate, harvest period and berry maturity, while molecular markers are independent of environmental condition and growth stage [31]. Twenty polymorphic SSR markers were used to identify 78 sea buckthorn accessions. The selected 23 SSR markers detected 2–5 alleles, and their PIC values ranged from 0.1166 to 0.6155 and had an average of 0.3249. The PIC mean value was significantly lower than that of RAPD, ISSR and SRAP markers previously reported [15−16, 32], suggesting that the gene sequences of these SSR markers were conserved in sea buckthorn germplasm.

Based on UPGMA, the 78 accessions were classified into two groups. There is a large genetic distance between accessions of ssp. *sinensis* and ssp. *mongolica*. The hybrids were in between and rather close to ssp. *mongolica* accessions. Coincidentally, these hybrids were also between ssp. *sinensis* and ssp. *mongolica* accessions on the

PCA plot based on 8 agronomic characters. This result illustrated that the diversity of morphological characters could reflect genetic diversity and be used as markers in agronomy. Ruan et al. [15] assessed 14 Chinese, Russian and Mongolian sea buckthorn accessions using RAPD markers and obtained similar results. In previous publication, the genetic relationship of 31 sea buckthorn accessions (also contained in this study) were analyzed based on 17 RNA-Seq SSRs [14]. However, the accessions of ssp. *mongolica* clustered in one group and those of ssp. *sinensis* and hybrids were divided in the other one. That revealed the genetic diversity relied on the diversity of genotypes and genetic backgrounds.

With the continuous development of high-throughput sequencing technology, transcriptome databases have become a powerful resource for SSR mining. More and more RNA-Seq SSRs have been developed and applied to the study of species genetic diversity and population genetic structure [33−34]. The SSRs obtained by transcriptomes are associated with many important quantitative traits [35].

The results in present study yielded useful knowledge regarding the diversity and genetic relationships of sea buckthorn germplasm in northern China, and could therefore facilitates further studies, including selection of mapping populations and promising candidates, marker-trait association analysis based on establishing the consistency of the traits , and characterizing parents used in future breeding programs.

Conclusion

In the present study, 8 phenotypic characteristics, oil traits in seeds and seedless parts,

and 23 SSR markers successfully distinguished all 78 sea buckthorn accessions. In PC analysis, BTD and HBW in the first PC were the most important characteristics for distinguishing the accessions. The agronomic traits of berries were closely correlated with the oil content and FA composition in pulp by CCA. This information will be valuable for germplasm identification and genotypic diversity analysis in *Hippophae rhamnoides*.

Supporting information

S1 Fig. 78 berry samples used in this study. Numbers are the variety codes as listed in Table 1.

(TIF)

S2 Fig. Total ion flow chromatography of 37 FAMEs Mix (A) and FAMEs in pulp oil in MHC (B).

(TIF)

S1 Table. Samples of sea buckthorn grouped according to different genetic backgrounds.

(DOCX)

S2 Table. Characterization of hybrids of sea buckthorn accessions studied.

(DOCX)

S3 Table. Geographical and climatic conditions at different sample collection sites of sea buckthorn in northern China.

(DOCX)

S4 Table. Primer sequences, annealing temperature, and estimated allelic size of 20 SSR markers.

(DOCX)

S5 Table. Descriptive statistics for morphological traits of berries and seeds among the sea buckthorn accessions studied.

(DOCX)

S6 Table. The morphological characteristics and oil traits of pulp and seeds of 78 sea buckthorn accessions studied.

(XLSX)

S7 Table. Fruit traits and Vc contents of large berry accessions of sea buckthorn in two experimental fields (located in Suiling and Dengkou).

(DOCX)

S8 Table. Allele combinations obtained at the 20 microsatellite loci in 78 sea buckthorn accessions.

(TXT)

Acknowledgements

The authors are grateful to Hai Guo (Jiuchenggong Breeding Base of Sea Buckthorn) and Jun Zhang (Institute of Selection and Breeding of *Hippophae*) for the collection of plant materials.

References

1. Bartish IV, Jeppsson N, Nybom H, Swenson U. Phylogeny of *Hippophae* (Elaeagnaceae) inferred from parsimony analysis of chloroplast DNA and morphology. Syst Bot. 2002; 27:41–54.

2. Swenson U and Bartish IV. Taxonomic synopsis of *Hippophae* (Elaeagnaceae). Nord J Bot. 2002; 22:369–374.

3. Teleszko M, Wojdyło A, Rudzińska M, Oszmiański J, Golis T. Analysis of Lipophilic and Hydrophilic Bioactive Compounds Content in Sea Buckthorn (*Hippophaë rhamnoides* L.) Berries. J Agric Food Chem. 2015; 63:4120−4129.

4. Pop MR, Weesepoel Y, Socaciu C, Pintea A, Vincken JP, Gruppen H. Carotenoid composition of berries and leaves from six Romanian sea buckthorn (*Hippophae rhamnoides* L.) varieties. Food Chem. 2014; 147:1−9.

5. Raffo A, Paoletti F, Antonelli M. Changes in sugar, organic acid, flavonol and carotenoid composition during ripening of berries of three seabuckthorn (*Hippophae rhamnoides* L.) cultivars. Eur Food Res Technol. 2004; 219:360−368.

6. Bal ML, Meda V, Naik NS, Satya S. Sea buckthorn: A potential source of valuable nutrients for nutraceuticals and cosmoceuticals. Food Res Int. 2011; 44:1718−1727.

7. Suryakumar G, Gupta A. Medicinal and therapeutic potential of Sea buckthorn (*Hippophae rhamnoides* L.). J Ethnopharmacol. 2011; 138:268−278.

8. Grey C, Widén C, Adlercreutz P, Rumpunen K, Duan RD. Antiproliferative effects of sea buckthorn (*Hippophae rhamnoides* L.) extracts on human colon and liver cancer cell lines. Food Chem.2010; 120: 1004−1010.

9. Xu YJ, Kaur M, Dhillon SR, Tappia SP, Dhalla SN. Health benefits of sea buckthorn for the prevention of cardiovascular diseases. J Func Foods. 2011; 3:2−12.

10. Yang B and Kallio H. Composition and Physiological Effects of Sea Buckthorn Lipids, Trends Food Sci Technol. 2002; 13:160–167.

11. Yang B and Kallio H. Fatty acid composition of lipids in sea buckthorn (*Hippophaë rhamnoides* L.) berries of different origins. J. Agric. Food Chem. 2001; 49:1939−1947.

12. Ruan CJ, Li H, Mopper S. Characterization and identification of ISSR markers associated with resistance to dried-shrink disease in sea buckthorn. Mol. Breeding. 2009; 24:255−268.

13. Ruan CJ, Teixeira da Silva JA, Jin H, Li H, Li DQ. Research and biotechnology in sea buckthorn (*Hippophae* spp.). Medicinal and Aromatic Plant Science and Biotechnology. 2007; 1: 47−60.

14. Li H, Ruan CJ, Wang L, Ding J, Tian XJ. Development of RNA-Seq SSR markers and application to genetic relationship analysis among sea buckthorn germplasm. J Amer Soc Hort Sci. 2017; 142(3):200-208.

15. Ruan CJ. Genetic relationships among some sea buckthorn cultivars from China, Russia and Mongolia using RAPD markers. Sci Hort. 2004; 101:417–426.

16. Li H, Ruan CJ, Teixeira da Silva J, Liu BQ. Associations of SRAP markers with dried-shrink disease resistance in a germplasm collection of sea buckthorn (*Hippophae* L.). Genome. 2010; 53:447–457.

17. Kalia RK, Rai MK, Kalia S, Singh R, Dhawan AK. Microsatellite markers: An overview of the recent progress in plants. Euphytica. 2011; 177:309–334.

18. Jain A, Chaudhary S, Sharma PC. Mining of microsatellites using next generation sequencing of seabuckthorn (*Hippophae rhamnoides* L.) transcriptome. Physiol. Mol. Biol. Plants. 2014; 20:115–123.

19. Patricia RR, Carmen GB, Beatriz CG, Jesús SG, Isabel T. Genotypic and phenotypic identification of olive cultivars from northwestern Spain and characterization of their extra

virgin olive oils in terms of fatty acid composition and minor compounds. Sci Hort. 2018; 232:269-279.

20. Tang X and Tigerstedt PMA. Variation of physical and chemical characters within an elite sea buckthorn (*Hippophae rhamnoides* L.) breeding population. Sci. Hort. 2001; 88(3):203–214.

21. Christie WW. A simple procedure for rapid transmethylation of glycerolipids and cholesteryl esters. J Lipid Res. 1982; 23:1072-1075.

22. Peakall R and Smouse PE. GENALEX 6: Genetic analysis in Excel. Population genetic software for teaching and research. Mol Ecol Notes. 2006; 6:288–295.

23. Peakall R and Smouse PE. GenAlEx 6.5: Genetic analysis in Excel. Population genetic software for teaching and research – An update. Bioinformatics. 2012; 28:2537–2539.

24. Liu K and Muse SV. PowerMarker: An integrated analysis environment for genetic marker analysis. Bioinformatics. 2005; 21:2128–2129.

25. Tamura SK, Peterson GD, Filipski A, Kumar S. MEGA6: Molecular evolutionary genetics analysis version 6.0. Mol Biol Evol. 2013; 30:2725–2729.

26. Lee ON and Park HY. Assessment of genetic diversity in cultivated radishes (*Raphanus sativus*) by agronomic traits and SSR markers. Sci Hort. 2017; 223:19-30.

27. Goodarzi S, Khadivi A, Abbasifar A, Akramian M. Phenotypic, pomological and chemical variations of the seedless barberry (*Berberis vulgaris* L. var. asperma). Sci Hort. 2018; 238:38–50.

28. Santos RC, Pires JL, Correa RX. Morphological characterization of leaf, flower, fruit and seed traits among Brazilian *Theobroma* L. species. Genet. Resour. Crop Evol. 2012; 59:327–345.

29. Kallio H, Yang B, Peippo P, Tahvonen R, Pan R. Triacylglycerols, glycerophospholipids, tocopherols and tocotrienols in sea buckthorn *Hippophae rhamnoides* L. ssp. *sinensis* and ssp.

mongolica berries and seeds. J. Agric. Food Chem. 2002; 50:3004–3009.

30. Kallio H, Yang B, Peippo P. Effects of Different Origins and Harvesting Time on Vitamin C, Tocopherols, and Tocotrienols in Sea Buckthorn (*Hippophae1 rhamnoides*) Berries. J. Agric. Food Chem. 2002; 50:6136−6142.

31. Ali M, Rajewski J, Baenziger P, Gill K, Eskridge KM, Dweikat I. Assessment of genetic diversity and relationship among a collection of US sweet sorghum germplasm by SSR markers. Mol. Breed. 2008; 21:497–509.

32. Li, H., C.J. Ruan, and J.A. Teixeira da Silva. 2009. Identification and genetic relationship based on ISSR analysis in a germplasm collection of sea buckthorn (*Hippophae* L.) from China and other countries. Sci. Hort. 123:263–271.

33. Liu YL, Zhang PF, Song ML, Hou JL, Qing M, Wang WQ, Liu CS. Transcriptome analysis and development of SSR molecular markers in *Glycyrrhiza uralensis* Fisch. PLoS One. 2015; 10:e0143017. doi:10.1371/journal.pone.0143017

34. Zhang LW, Li YR, Tao AF, Fang PP, Qi JM. Development and characterization of 1,906 EST-SSR markers from unigenes in jute (*Corchorus* spp.), PLoS One. 2015; 10(10):e0140861. doi:10.1371/journal.pone.0140861

35. Ramchiary N, Nguyen VD, Li X, Hong CP, Dhandapani V, Choi SR, et al. Genic microsatellite markers in *Brassica rapa*: Development, characterization, mapping, and their utility in other cultivated and wild *Brassica* relatives. DNA Res. 2011; 18:305–320.

Response to Reviewer' Comments letter to PLOS ONE

The authors thank the additional editor and two reviewers for their careful reading, comments and suggestion. We revised our manuscript in the best way as we could. Revised portions are marked in red in the revised manuscript. For the individual comments see our reply below.

Additional Editor Comments:

1) The use of term varieties, cultivars, subspecies and hybrids have been without much explanation. For example, what is the basis of assigning hybrid status to a particular cultivar? More clarity is required in explanation of the material. How these varieties were assigned varietal status?

Response: The Reviewer 1 gave the definition of 'variety' that "a variety must be recognizable by its characteristics, recognizably different from any other variety and remain unchanged through the process of propagation". The 'cultivar' refers to a variety of a plant developed from a natural species and maintained under cultivation. The authors accepted the reviewers' advice that the term 'accessions' would be appropriate according to the plant materials in present study.

A subspecies of a plant is one of the types that a particular species is divided into. In this study, ssp. *sinensis* and ssp. *mongolica* are two subspecies of *Hippophae rhamnoides*.

The hybrid accessions in this study generated by hybridization experiment in control between ssp. *mongolica* and ssp. *sinensis* at specialized experimental fields and selected for their desirable traits. After a complex process of identification of experts, some hybrids may became a new 'cultivar'.

2) Generation of morphological dataset is also not mentioned clearly. You have 76 varieties growing at three locations. You need to provide environmental parameters for each location. Are all 76 growing at each location? If all the varieties are not growing at same location, many of morphological traits will be influenced by environmental factors. Did you do any multilocation trials to see the influence of environment on these traits? Did you try to collect data during different years and see if the data is consistent or showing variation. A statistical analysis of such data only will generate confidence in morphological data. Even a multilocation trial of a subset will provide information on reliability of data. Please include such data.

Response: The environmental parameters for each location were provided in the S3 Table of revised manuscript. All the accessions are not growing at same location. However, they could adapt to the environment of their cultivated lands well. We had ever performed some multi-location trials to see the influence of environment on fruit characteristics that was supplemented in the 'Results' of revised manuscript (S7 Table).

'In previous mutilocation trials in Suiling (47°14′N, 127°06′E; 202 m) and Dengkou (40°43′N, 106°30′E; 1053m, Inner Mongolia), the fruit characteristics of 11 large berry accessions (AET, CS, CY, HJ, JS, JR, XY, YS, KTN, WLGM and SJ1) were comparatively analyzed (S7 Table). The

HBWs of them in Suiling (38.33–67.59 g) were higher than those in Dengkou (32.87–63.85). For all the introduced cultivars, the HBWs in two experimental fields were lower than those in their country of origin, Russia. The phenotypic characteristics of sea buckthorn berries showed differences due to their origins, berry parts analyzed, climate and growing conditions. In this study, the 78 accessions were selected for their adaptabilities to growth sites.'

3) The sequencing data has been published earlier and 17 of SSR are coming from that data. Only 3 new markers have been used in the present study. This undermines the amount of data presented in this MS. You have to clearly mention these facts in the MS and the abstract. In my opinion more data needs to be generated. I suggest another 25-30 SSRs should be used for analyzing the diversity.

Response: We have mentioned it in the MM and the abstract of the revised manuscript. We screened 3 new SSR loci (SB21-23) with polymorphism from 20 SSR primer pairs during the revision of the manuscript. These information has been supplemented in revised manuscript. It is difficult to develop more RNA-Seq SSRs. On one hand, the genic sequences used for developing SSR markers were rather conserved in the sea buckthorn germplasm. On the other hand, the species and subspecies of sea buckthorn germplasm used in this study are limited to facilitate more polymorphism at SSR loci.

4) There is no comparison given between the varieties used in previous publication and the present one. Are you using common varities? If you are than SSR data must be same and must have been presented in previous MS already. This has not been mentioned in the MS.

Response: In previous publication, 31 accessions (contained in the present one) were used for the validation of developed SSR markers. They included 6 accessions of ssp. *sinensis*, 14 accessions of ssp. *mongolica* and 11 hybrid accessions. They were selected according to their genetic origins and cultivated lands. In present study, the accessions were selected based on various fruit traits. The results of genetic relationship were different from that in the previous publication. That was supplemented in the discussion of revised manuscript.

'In previous publication, the genetic relationship of 31 sea buckthorn accessions (also contained in the 78 accessions) were analyzed based on 17 RNA-Seq SSRs [14]. However, the accessions of ssp. *mongolica* clustered in one group and those of ssp. *sinensis* and hybrids were in the other one. That revealed the genetic diversity relied on the genotypes and genetic backgrounds.'

Reviewer #1:

Specific comments

1. I think that in such bio-prospection studies sampling strategy is very crucial. The sampling method needs to explain that how these accessions were sourced. The MS needs elaboration on –

• How many individuals of a "variety" from each site were collected?

• Are these the random collections of registered varieties from the cultivated field in the five regions OR sampled from the wild?

• It is also not clear that how the hybrids were distinguished from parents while making collections.
• Do these sites differ in climatic conditions?
• What is the link of “origin” with oil content? Did you expect that there are bound to be differences because of differences in the climatic conditions of area of collection/cultivation of the same “variety/hybrid”?

Importantly instead of the term varieties the term accessions would be appropriate, as the authors have mentioned it in Table 1. According to the definition by The International Union for the Protection of New Varieties of Plants, "a variety must be recognizable by its characteristics, recognizably different from any other variety and remain unchanged through the process of propagation".

Do these two subspecies hybridize freely in nature and such hybrids have been characterized? This needs some population analysis like by using STRUCTURE, or at least there should be a note on the characterization of hybrids (including the features), even if they are procured form some Research Institute.

Response: The part of ‘Plant materials’ in original text was revised according to above advice.
(1) 235 individuals (2−5 ramet plants each accession) in 5 growth sites were collected.
(2) These are registered accessions from the cultivated field and adapt to the local environment well.
(3) For the identification of the hybrid accessions, they are labelled and recorded with documents. Furthermore, most hybrid accessions and their parents are not in the same growth site. The parents of them are cultivated in the experimental field for hybridization.
(4) The growth sites differ in climatic conditions which are described in S3 Table.
(5) According to the results in this study, the oil contents in pulp and seeds are highest in ssp. *mongolica* accessions on average. That is the link of origin with oil content. In this study, we did not consider the difference in the climatic conditions of cultivated fields for the sea buckthorn accessions we selected adapted local environment well.
(6) The authors agreed the opinion that the term ‘accessions’ would be appropriate and all the term ‘varieties’ were revised to the term ‘accessions’ in the revised manuscript.
(7) These two subspecies hybridized by experiments in control which were performed in specialized experimental fields. The authors accepted the advice about the note on the characterization of hybrids which was described in S2 Table

2. I don’t understand the usage of term pulp/peel in the MS (also see page 15, line 251). As the entire fleshy region was separately used for extraction of oil from the "berries" (see Methods), the use of term pulp would be appropriate. One cannot expect to remove the epidermal peel especially during the mechanical homogenization process.
Response: The authors accepted the advice and the phrase ‘pulp/peel’ in the original text was replaced to ‘pulp’ in the revised manuscript.

3. How the present study for the genetic diversity analysis of 78 cultivars is different from other previous studies? May be highlighted in the introduction. Authors may also highlight that trait: i.e. Oil yield was correlated with the “promising” accessions.
Response: The related content has been supplemented in the introduction of the revised manuscript.

‘The diversity analysis helps understand the relationships between germplasm characters and genotype will improve the sea buckthorn germplasm to achieve higher production with higher quality for the important traits were correlated with the promising germplasm [19].
In present study, 78 accessions of sea buckthorn with large variation of fruit traits were selected as materials.’

4. Although attempt has been made of possible use of MAB in future, but it has not been justified with the discussion. For example, do the authors will depend on the same plants in the cultivated lands across the region or some mapping populations will be established. In former case GPS tagging of the individuals will be required for sourcing the material on regular basis and to establish the consistency of the trait.
Response: The results in present study yielded useful knowledge regarding the diversity and genetic relationships of sea buckthorn germplasm in northern China, and could therefore facilitates further studies, including selection of mapping populations and promising candidates, marker-trait association analysis based on establishing the consistency of the traits , and characterizing parents used in future breeding programs. The above information on possible use of MAB in future has been supplemented in the discussion of the revised manuscript.

Materials and Methods

5. Need to mention whether hundred-berry weight, hundred-seed weight and other dimensions were taken from mature or immature berries? In Supplementary figure 1 some samples are showing immature berries e.g. sample 65, 68 etc.
Response: The hundred-berry weight, hundred-seed weight and other dimensions should be taken from mature berries. So the berries of all accessions were collected from the end of July to mid-September, according to their ripening stages. But it is difficult to collected ripening fruits of 78 sea buckthorn accessions. The berries of several accession were harvested when they are approaching maturity. So the data error existed in the dimensions of several accessions. The authors admitted it and hope be understood at this point.

6. What do the ‘Berry Shape Indices’ refer to and what are its implications on the results/oil trait/ with genetic diversity. Provide any suitable reference if possible. (Page: 8, subsection: Morphological….)
Response: The berry shape index (BSI) is estimated by the ratio of BLD to BTD, also called length/width ratio in some studies, which indicates berry shape. According to the results in present study, the phenotypic characters (BLD, HBW, BSI, and BTD) of berries and oil traits in pulp showed close correlation (r = 0.8725, p = 0.0000) using CCA. The relevant literature is below. The results of it showed that the morphological traits established were consistent with those derived from the SSR markers in olive plant materials. The length/width ratio was one of the morphological traits of endocarp in that study.

Patricia RR, Carmen GB, Beatriz CG, Jesús SG, Isabel T. Genotypic and phenotypic identification of olive cultivars from northwestern Spain and characterization of their extra virgin olive oils in terms of fatty acid composition and minor compounds. Sci Hort. 2018; 232:269-279.

7. The usage of phrase ‘8 agronomic traits’ seems to be superfluous as these are the traits of berries itself. How the seed width is different from the seed thickness? The difference is not apparent. Table 2 and 3; as well as in text.

Response: For sea buckthorn, the traits of berries (including seeds) are very important for their economic value. The seed thickness could be regarded as the ‘height’ of seeds, which is a parameter of oilseed, e.g. olive.

8. The usage of abbreviation has not been followed see table 2 and 3. Table 2 is not necessary, may be omitted or shifted to Supplementary Data. In Tables SD is not mentioned.

Response: The authors accepted the advice. Table 2 was shifted to S5 Table. The data of ‘Mean ± SD’ was given.

9. The reference is missing for the SB18-SB20 SSRs; in the text (Page 10, line 181).

Response: The SB18-SB20 SSRs were firstly reported in this study and no reference could be given for them.

Results

10. Results should be given in the format mean ± SD. Minimum and maximum can be given in supplementary tables.

Response: The authors accepted the advice and the results have been given in the format mean ± SD.

11. It is not clear from the table caption and content that whether values in the Table 4 is the minimum, maximum and mean values are representing the cumulative results of 78 varieties e.g. minimum in variety… and maximum in variety…. Need to mention in the results.

Response: The authors accepted the advice. The table caption in the Table 4 of original text is not clear because we want to use the abbreviation of ‘minimum, maximum’ but the notes were forgotten to give bellow the table. And the note has been added in the ‘Results’ in the revised manuscript.

12. The results of CCA are driving a correlation between phenotypic traits and oil characteristics. The authors may use the information for total oil content (pulp+seed) or oil content in pulp and seeds separately for drawing any correlation. That would possibly help as a descriptor for the potential crop in identifying the elite/superior “variety” and further can be linked to genetic diversity.

Response: Due to the difference in the FAs composition between pulp oil and seed oil, the oil contents in pulp and seeds were separately used for drawing any correlation. In practical production, the seed oil and pulp oil are also separately extracted for their different functions. Furthermore, during the course of CCA, the factors in each data matrix would be analyzed by pairwise correlation analysis which was sufficient for drawing any correlation.

Discussion

13. Page:28, Line:449-453. The link of this part of discussion is lacking with the previous text.

Response: In the part of ‘Introduction’, the superiority of SSR markers was mentioned. The significance of developing SSR markers with RNA-Seq technique was also mentioned in it. The SSR markers used in this study are developed by RNA-Seq. All these description was the link of this part of discussion.

14. In conclusion part authors are concluding that this information may be useful for cultivar identification but initially they started their work for the varieties. Taxonomically these two are different entities.
Response: The authors agreed this opinion. The phrase ‘cultivar identification’ was revised to ‘germplasm identification’ and all the terms ‘varieties’ were replaced to ‘accessions’ in the revised manuscript according to the taxonomical definition.

Some suggestion:
1. The sequence of S1 and S2 table can be reversed as per the citation in the text.
Response: The good advice mentioned above is accepted by the authors. The tables were reversed in the revised manuscript.

2. Page:3, Line:54. Reference 1 is incorrect. The lead author here is Bartish I.V.
Response: The authors in reference 1 were corrected in the revised manuscript.

Bartish IV, Jeppsson N, Nybom H, Swenson U. Phylogeny of *Hippophae* (Elaeagnaceae) inferred from parsimony analysis of chloroplast DNA and morphology. Syst Bot. 2002; 27:41–54.

3. Page:3, Line:56-57. ….flavonoids [3-7]; ….products [8-10]. Here over-citation may be avoided.
Response: The authors accepted the advice and the references cited in the two sentences were cut down in the revised manuscript.

4. Page:3, Line:59. ‘Sea buckthorn oil’ instead of ‘sea buckthorn oils’
Response: The phrase was corrected in the revised manuscript.

5. Page: 4, Line 74. Add a reference to the statement. The plant is able to avoid cold and is not resistant, because the leaves are shed under extreme cold condition in this plant. Even the species is not resistant to alkali too.
Response: The authors agreed this opinion and this sentence was revised to ‘Sea buckthorn adapts well to extreme conditions, including drought, salinity, alkalinity, and temperatures [12]’ in the revised manuscript.

12. Ruan CJ, Li H, Mopper S. Characterization and identification of ISSR markers associated with resistance to dried-shrink disease in sea buckthorn. Mol. Breeding. 2009; 24:255−268.

6. Page:4. Line:85. Use full form at first place ‘MAB’.
Response: The sentence was corrected in the revised manuscript and the full form ‘molecular marker-assisted breeding’ was used at first place ‘MAB’.

7. Page:5. Line:110. What was the premise of including two known elite varieties in the study? Any supportive reference(s) for the statement, and also mention the context in which these varieties are elite.

Response: The premise of elite accessions include high yield, good agronomic traits and strong adaptability to environment, etc. Some Chinese references support that Quyisike and Zhongguoshaji^{wild} are elite cultivars. The word ‘elite’ in the sentence was deleted in the revised manuscript for no English reference supported it.

8. Page:12. Line:204-205. May be included in Material and Methods.

Response: The authors accepted the advice and the sentence ‘Minimum, maximum, mean, standard deviation (SD), and coefficient of variation (CV%) were recorded.’ was added in Material and Methods of the revised manuscript.

Reviewer #2:

1. The authors mention that 76 varieties were used. There is no mention of the different species they belonged to in M&M, although it has been mentioned later in the text and table. Incorporate that information in the M&M.

Response: The good advice mentioned above is accepted by the authors. The related information has been added in M&M of the revised manuscript.

2. Are these 76 different varieties or just different accessions? At many places they are being referred to as ‘cultivars’ also. Please correct accordingly in the text wherever mentioned.

Response: After careful consideration, the authors thought ‘accessions’ would be appropriate. The 'varieties' has been replaced into ‘accessions’ in the revised manuscript.

3. How variable are the climatic conditions of the three research institutes?

Response: The climatic conditions of different growth sites of sea buckthorn samples has been added in S3 Table of the revised manuscript, with the caption ‘Geographical and climatic conditions at different sample collection sites of sea buckthorn in northern China’.

4. Line 109: ‘………provided 76 varieties’. Does this mean that all the 76 were grown at all the 3 fields? There is no clarity on this aspect in the M&M. Most quantitative traits exhibit a huge variation across environments. To study the phenotypic variations it would have been much informative if all the 76 varieties were grown together across all the three fields. Why was that not considered?

Response: Among the 76 accessions of sea buckthorn samples, 12 were grown in the Institute of Selection and Breeding of *Hippophae*, 52 were grown in the Research Institute of Berry and 12 were grown in the Jiuchenggong Breeding Base of Sea Buckthorn. These accessions are able to adapt to local climate and screened to be excellent germplasm.

The authors agreed the opinion that most quantitative traits exhibit a huge variation across environments. We did the comparative analysis on fruit morphological traits of the same cultivars grown in different cultivated fields in our early studies and the data was complemented in the ‘Results’ (S5 Table) of the revised manuscript. The aim in this study is to further screen the elite

accessions from the 78 accession with good adaption to the environments of cultivated fields and prepare for the next step of MAB. In the follow-up study, the continuous observation of the environmental factors would be considered.

5. There is no mention of how these varieties were grown in the field, and data from how many plants were considered for the morphological and oil analysis. For eg. for hundred berry weight (HBW), berries were collected from how many different plants?

Response: The information has been supplemented in the introduction of the revised manuscript. The sea buckthorn samples in this study are collected from 235 individuals (2−5 ramet plants each accession) in 5 growth sites. The berries of each accession were pooled and frozen as quickly as possible at −20 °C. When all plant materials were harvested, the berries were transferred to –50 °C for storage until analysis. The related information has been supplemented in the M&M and Table 1.

6. Line 137: For the oil extraction and FA analysis, the authors mention that ‘each sample was analyzed three times’. Why weren’t three biological replicates taken for this analysis?

Response: The authors are sorry for the incorrect expression. In this study**,** three biological replicates were taken for every analysis. The sentence is corrected in the revised manuscript.

7. Line 180-181: The authors have used 17 previously developed SSR markers and 3 newly developed SSR markers using RNA-Seq. What was the basis of selection of just 3 new markers from the RNA-Seq. Why weren’t more markers deployed for the genetic characterization?

Response: The authors obtained many SSR sequences using RNA-Seq method and designed the primers to screen those SSR loci with polymorphism in sea buckthorn cultivars. We reported 17 developed SSR markers at first. In subsequent experiments, we screened 3 new SSR markers which also showed polymorphic amplification in sea buckthorn germplasm. RNA-Seq SSR loci with polymorphism in sea buckthorn germplasm were difficult to develop for that SSR markers derived from expressed region of genome showed high conservation to some extent in our study. That’s why no more markers deployed for the genetic characterization in sea buckthorn at now.

8. Line 180: Please reframe the sentence. It appears that the authors have done RNA-seq to generate the 3 new SSR markers. Although, the RNA-Seq had been done in previous study from where the 17 SSR were also developed (Reference 17).

Response: The authors accepted the advice and the sentence has been revised into ‘The Twenty polymorphic microsatellite loci (SSR) developed using RNA-Seq were evaluated and loci SB1-SB17 were previously published [17]’ in the revised manuscript.

9. Instead of ‘different origins’ that has been used repeatedly by authors throughout the text and tables, I suggest use the two different species and hybrid accessions.

Response: The authors accepted the good advice. Some ‘different origins’ were replaced to the ‘two different subspecies and hybrid accessions’ and the others were deleted in the revised manuscript.

10. Line 340: ‘All the primers’. Reframe this line. All primers did not give 59 bands. A total of 59 bands were amplified.

Response: The sentence has been revised according to the advice in the revised manuscript.

‘A total of 69 bands were amplified using the 23 primer pairs, of which 59 were polymorphic, accounting for 85.51% of all.’

11. Line 341: ‘accounting for 86.44%’ . Incomplete sentence, 86.44% of what??
Response: The sentence has been revised according to the advice in the revised manuscript.

12. Line 372: the 3 subgroups have been referred incorrectly. They are IIa, IIb and IIc.
Response: The names of 3 subgroups were corrected in the revised manuscript.

13. Line 421: ‘in comparison of populations’. Statement not clear. Please reframe.
Response: The phrase has been replaced to ‘in population identification’ in the revised manuscript.

14. Line 436: ‘gene sequences’. Are all the SSR markers used genic in nature?
Response: SSR can be divided into genomic SSRs and genic SSRs because of the resource of sequences used for SSR identification. Genic SSRs derived from transcriptome or expressed sequence tag sequences are located in expressed genes. These markers can be linked with important phenotypic characteristics through quantitative trait loci analysis. In this study, all SSR markers are genic SSRs.

15. Table 1: Could just be described as the ‘Accessions of sea buckthorn used for the study’
Response: The authors accepted the good advice and the title of Table 1 was revised into the ‘Accessions of sea buckthorn used for the study’.
