# Supplementary material for: Diversity in sea buckthorn (Hippophae rhamnoides L.) accessions with different origins based on morphological characteristics, oil traits, and microsatellite markers
Source: PLoS One. 2020 Mar 13;15(3):e0230356. doi: 10.1371/journal.pone.0230356 (PMC7069629; doi:10.1371/journal.pone.0230356)
Supplement: Attachment — Submitted filename: Response to Reviewers.docx [file pone.0230356.s012.docx]

Response to Reviewer' Comments letter to PLOS ONE

The authors thank the additional editor and two reviewers for their careful reading, comments and suggestion. We revised our manuscript in the best way as we could. Revised portions are marked in red in the revised manuscript. For the individual comments see our reply below.

**Additional Editor Comments:**

1) The use of term varieties, cultivars, subspecies and hybrids have been without much explanation. For example, what is the basis of assigning hybrid status to a particular cultivar? More clarity is required in explanation of the material. How these varieties were assigned varietal status?

**Response:** The Reviewer 1 gave the definition of ‘variety’ that "a variety must be recognizable by its characteristics, recognizably different from any other variety and remain unchanged through the process of propagation". The ‘cultivar’ refers to a variety of a plant developed from a natural species and maintained under cultivation. The authors accepted the reviewers’ advice that the term ‘accessions’ would be appropriate according to the plant materials in present study.

A subspecies of a plant is one of the types that a particular species is divided into. In this study, ssp. *sinensis* and ssp. *mongolica* are two subspecies of *Hippophae* *rhamnoides*.

The hybrid accessions in this study generated by hybridization experiment in control between ssp. *mongolica* and ssp. *sinensis* at specialized experimental fields and selected for their desirable traits. After a complex process of identification of experts, some hybrids may became a new ‘cultivar’.

2) Generation of morphological dataset is also not mentioned clearly. You have 76 varieties growing at three locations. You need to provide environmental parameters for each location. Are all 76 growing at each location? If all the varieties are not growing at same location, many of morphological traits will be influenced by environmental factors. Did you do any multilocation trials to see the influence of environment on these traits? Did you try to collect data during different years and see if the data is consistent or showing variation. A statistical analysis of such data only will generate confidence in morphological data. Even a multilocation trial of a subset will provide information on reliability of data. Please include such data.

**Response:** The environmental parameters for each location were provided in the S3 Table of revised manuscript. All the accessions are not growing at same location. However, they could adapt to the environment of their cultivated lands well. We had ever performed some multi-location trials to see the influence of environment on fruit characteristics that was supplemented in the ‘Results’ of revised manuscript (S7 Table).

‘In previous mutilocation trials in Suiling (47°14′N, 127°06′E; 202 m) and Dengkou (40°43′N, 106°30′E; 1053m, Inner Mongolia), the fruit characteristics of 11 large berry accessions (AET, CS, CY, HJ, JS, JR, XY, YS, KTN, WLGM and SJ1) were comparatively analyzed (S7 Table). The HBWs of them in Suiling (38.33–67.59 g) were higher than those in Dengkou (32.87–63.85). For all the introduced cultivars, the HBWs in two experimental fields were lower than those in their country of origin, Russia. The phenotypic characteristics of sea buckthorn berries showed differences due to their origins, berry parts analyzed, climate and growing conditions. In this study, the 78 accessions were selected for their adaptabilities to growth sites.’

3) The sequencing data has been published earlier and 17 of SSR are coming from that data. Only 3 new markers have been used in the present study. This undermines the amount of data presented in this MS. You have to clearly mention these facts in the MS and the abstract. In my opinion more data needs to be generated. I suggest another 25-30 SSRs should be used for analyzing the diversity.

**Response:** We have mentioned it in the MM and the abstract of the revised manuscript. We screened 3 new SSR loci (SB21-23) with polymorphism from 20 SSR primer pairs during the revision of the manuscript. These information has been supplemented in revised manuscript. It is difficult to develop more RNA-Seq SSRs. On one hand, the genic sequences used for developing SSR markers were rather conserved in the sea buckthorn germplasm. On the other hand, the species and subspecies of sea buckthorn germplasm used in this study are limited to facilitate more polymorphism at SSR loci.

4) There is no comparison given between the varieties used in previous publication and the present one. Are you using common varities? If you are than SSR data must be same and must have been presented in previous MS already. This has not been mentioned in the MS.

**Response:** In previous publication, 31 accessions (contained in the present one) were used for the validation of developed SSR markers. They included 6 accessions of ssp. *sinensis*, 14 accessions of ssp. *mongolica* and 11 hybrid accessions. They were selected according to their genetic origins and cultivated lands. In present study, the accessions were selected based on various fruit traits. The results of genetic relationship were different from that in the previous publication. That was supplemented in the discussion of revised manuscript.

‘In previous publication, the genetic relationship of 31 sea buckthorn accessions (also contained in the 78 accessions) were analyzed based on 17 RNA-Seq SSRs [14]. However, the accessions of ssp. *mongolica* clustered in one group and those of ssp. *sinensis* and hybrids were in the other one. That revealed the genetic diversity relied on the genotypes and genetic backgrounds.’

**Reviewer #1:**

**Specific comments**

1. I think that in such bio-prospection studies sampling strategy is very crucial. The sampling method needs to explain that how these accessions were sourced. The MS needs elaboration on –

• How many individuals of a “variety” from each site were collected?

• Are these the random collections of registered varieties from the cultivated field in the five regions OR sampled from the wild?

• It is also not clear that how the hybrids were distinguished from parents while making collections.
• Do these sites differ in climatic conditions?

• What is the link of “origin” with oil content? Did you expect that there are bound to be differences because of differences in the climatic conditions of area of collection/cultivation of the same “variety/hybrid”?

Importantly instead of the term varieties the term accessions would be appropriate, as the authors have mentioned it in Table 1. According to the definition by The International Union for the Protection of New Varieties of Plants, "a variety must be recognizable by its characteristics, recognizably different from any other variety and remain unchanged through the process of propagation".

Do these two subspecies hybridize freely in nature and such hybrids have been characterized? This needs some population analysis like by using STRUCTURE, or at least there should be a note on the characterization of hybrids (including the features), even if they are procured form some Research Institute.

**Response:** The part of ‘Plant materials’ in original text was revised according to above advice.

(1) 235 individuals (2−5 ramet plants each accession) in 5 growth sites were collected.

(2) These are registered accessions from the cultivated field and adapt to the local environment well.

(3) For the identification of the hybrid accessions, they are labelled and recorded with documents. Furthermore, most hybrid accessions and their parents are not in the same growth site. The parents of them are cultivated in the experimental field for hybridization.

(4) The growth sites differ in climatic conditions which are described in S3 Table.

(5) According to the results in this study, the oil contents in pulp and seeds are highest in ssp. *mongolica* accessions on average. That is the link of origin with oil content. In this study, we did not consider the difference in the climatic conditions of cultivated fields for the sea buckthorn accessions we selected adapted local environment well.

(6) The authors agreed the opinion that the term ‘accessions’ would be appropriate and all the term ‘varieties’ were revised to the term ‘accessions’ in the revised manuscript.

(7) These two subspecies hybridized by experiments in control which were performed in specialized experimental fields. The authors accepted the advice about the note on the characterization of hybrids which was described in S2 Table

2. I don’t understand the usage of term pulp/peel in the MS (also see page 15, line 251). As the entire fleshy region was separately used for extraction of oil from the "berries" (see Methods), the use of term pulp would be appropriate. One cannot expect to remove the epidermal peel especially during the mechanical homogenization process.

**Response:** The authors accepted the advice and the phrase ‘pulp/peel’ in the original text was replaced to ‘pulp’ in the revised manuscript.

3. How the present study for the genetic diversity analysis of 78 cultivars is different from other previous studies? May be highlighted in the introduction. Authors may also highlight that trait: i.e. Oil yield was correlated with the “promising” accessions.

**Response:** The related content has been supplemented in the introduction of the revised manuscript.

‘The diversity analysis helps understand the relationships between germplasm characters and genotype will improve the sea buckthorn germplasm to achieve higher production with higher quality for the important traits were correlated with the promising germplasm [19].

In present study, 78 accessions of sea buckthorn with large variation of fruit traits were selected as materials.’

4. Although attempt has been made of possible use of MAB in future, but it has not been justified with the discussion. For example, do the authors will depend on the same plants in the cultivated lands across the region or some mapping populations will be established. In former case GPS tagging of the individuals will be required for sourcing the material on regular basis and to establish the consistency of the trait.

**Response:** The results in present study yielded useful knowledge regarding the diversity and genetic relationships of sea buckthorn germplasm in northern China, and could therefore facilitates further studies, including selection of mapping populations and promising candidates, marker-trait association analysis based on establishing the consistency of the traits , and characterizing parents used in future breeding programs. The above information on possible use of MAB in future has been supplemented in the discussion of the revised manuscript.

**Materials and Methods**

5. Need to mention whether hundred-berry weight, hundred-seed weight and other dimensions were taken from mature or immature berries? In Supplementary figure 1 some samples are showing immature berries e.g. sample 65, 68 etc.

**Response:** The hundred-berry weight, hundred-seed weight and other dimensions should be taken from mature berries. So the berries of all accessions were collected from the end of July to mid-September, according to their ripening stages. But it is difficult to collected ripening fruits of 78 sea buckthorn accessions. The berries of several accession were harvested when they are approaching maturity. So the data error existed in the dimensions of several accessions. The authors admitted it and hope be understood at this point.

6. What do the ‘Berry Shape Indices’ refer to and what are its implications on the results/oil trait/ with genetic diversity. Provide any suitable reference if possible. (Page: 8, subsection: Morphological….)

**Response:** The berry shape index (BSI) is estimated by the ratio of BLD to BTD, also called length/width ratio in some studies, which indicates berry shape. According to the results in present study, the phenotypic characters (BLD, HBW, BSI, and BTD) of berries and oil traits in pulp showed close correlation (*r* = 0.8725, *p* = 0.0000) using CCA. The relevant literature is below. The results of it showed that the morphological traits established were consistent with those derived from the SSR markers in olive plant materials. The length/width ratio was one of the morphological traits of endocarp in that study.

Patricia RR, Carmen GB, Beatriz CG, Jesús SG, Isabel T. Genotypic and phenotypic identification of olive cultivars from northwestern Spain and characterization of their extra virgin olive oils in terms of fatty acid composition and minor compounds. Sci Hort. 2018; 232:269-279.

7. The usage of phrase ‘8 agronomic traits’ seems to be superfluous as these are the traits of berries itself. How the seed width is different from the seed thickness? The difference is not apparent. Table 2 and 3; as well as in text.

**Response:** For sea buckthorn, the traits of berries (including seeds) are very important for their economic value. The seed thickness could be regarded as the ‘height’ of seeds, which is a parameter of oilseed, e.g. olive.

8. The usage of abbreviation has not been followed see table 2 and 3. Table 2 is not necessary, may be omitted or shifted to Supplementary Data. In Tables SD is not mentioned.

**Response:** The authors accepted the advice. Table 2 was shifted to S5 Table. The data of ‘Mean ± SD’ was given.

9. The reference is missing for the SB18-SB20 SSRs; in the text (Page 10, line 181).

**Response:** The SB18-SB20 SSRs were firstly reported in this study and no reference could be given for them.

**Results**

10. Results should be given in the format mean ± SD. Minimum and maximum can be given in supplementary tables.

**Response:** The authors accepted the advice and the results have been given in the format mean ± SD.

11. It is not clear from the table caption and content that whether values in the Table 4 is the minimum, maximum and mean values are representing the cumulative results of 78 varieties e.g. minimum in variety… and maximum in variety…. Need to mention in the results.

**Response:** The authors accepted the advice. The table caption in the Table 4 of original text is not clear because we want to use the abbreviation of ‘minimum, maximum’ but the notes were forgotten to give bellow the table. And the note has been added in the ‘Results’ in the revised manuscript.

12. The results of CCA are driving a correlation between phenotypic traits and oil characteristics. The authors may use the information for total oil content (pulp+seed) or oil content in pulp and seeds separately for drawing any correlation. That would possibly help as a descriptor for the potential crop in identifying the elite/superior “variety” and further can be linked to genetic diversity.

**Response:** Due to the difference in the FAs composition between pulp oil and seed oil, the oil contents in pulp and seeds were separately used for drawing any correlation. In practical production, the seed oil and pulp oil are also separately extracted for their different functions. Furthermore, during the course of CCA, the factors in each data matrix would be analyzed by pairwise correlation analysis which was sufficient for drawing any correlation.

**Discussion**

13. Page:28, Line:449-453. The link of this part of discussion is lacking with the previous text.

**Response:** In the part of ‘Introduction’, the superiority of SSR markers was mentioned. The significance of developing SSR markers with RNA-Seq technique was also mentioned in it. The SSR markers used in this study are developed by RNA-Seq. All these description was the link of this part of discussion.

14. In conclusion part authors are concluding that this information may be useful for cultivar identification but initially they started their work for the varieties. Taxonomically these two are different entities.

**Response:** The authors agreed this opinion. The phrase ‘cultivar identification’ was revised to ‘germplasm identification’ and all the terms ‘varieties’ were replaced to ‘accessions’ in the revised manuscript according to the taxonomical definition.

**Some suggestion:**

1. The sequence of S1 and S2 table can be reversed as per the citation in the text.

**Response:** The good advice mentioned above is accepted by the authors. The tables were reversed in the revised manuscript.

2. Page:3, Line:54. Reference 1 is incorrect. The lead author here is Bartish I.V.

**Response:** The authors in reference 1 were corrected in the revised manuscript.

Bartish IV, Jeppsson N, Nybom H, Swenson U. Phylogeny of *Hippophae* (Elaeagnaceae) inferred from parsimony analysis of chloroplast DNA and morphology. Syst Bot. 2002; 27:41–54.

3. Page:3, Line:56-57. ….flavonoids [3-7]; ….products [8-10]. Here over-citation may be avoided.

**Response:** The authors accepted the advice and the references cited in the two sentences were cut down in the revised manuscript.

4. Page:3, Line:59. ‘Sea buckthorn oil’ instead of ‘sea buckthorn oils’

**Response:** The phrase was corrected in the revised manuscript.

5. Page: 4, Line 74. Add a reference to the statement. The plant is able to avoid cold and is not resistant, because the leaves are shed under extreme cold condition in this plant. Even the species is not resistant to alkali too.

**Response:** The authors agreed this opinion and this sentence was revised to ‘Sea buckthorn adapts well to extreme conditions, including drought, salinity, alkalinity, and temperatures [12]’ in the revised manuscript.

12. Ruan CJ, Li H, Mopper S. Characterization and identification of ISSR markers associated with resistance to dried-shrink disease in sea buckthorn. Mol. Breeding. 2009; 24:255−268.

6. Page:4. Line:85. Use full form at first place ‘MAB’.

**Response:** The sentence was corrected in the revised manuscript and the full form ‘molecular marker-assisted breeding’ was used at first place ‘MAB’.

7. Page:5. Line:110. What was the premise of including two known elite varieties in the study? Any supportive reference(s) for the statement, and also mention the context in which these varieties are elite.

**Response:** The premise of elite accessions include high yield, good agronomic traits and strong adaptability to environment, etc. Some Chinese references support that Quyisike and Zhongguoshaji*^wild^* are elite cultivars. The word ‘elite’ in the sentence was deleted in the revised manuscript for no English reference supported it.

8. Page:12. Line:204-205. May be included in Material and Methods.

**Response:** The authors accepted the advice and the sentence ‘Minimum, maximum, mean, standard deviation (SD), and coefficient of variation (CV%) were recorded.’ was added in Material and Methods of the revised manuscript.

**Reviewer #2:**

1. The authors mention that 76 varieties were used. There is no mention of the different species they belonged to in M&M, although it has been mentioned later in the text and table. Incorporate that information in the M&M.

**Response:** The good advice mentioned above is accepted by the authors. The related information has been added in M&M of the revised manuscript.

2. Are these 76 different varieties or just different accessions? At many places they are being referred to as ‘cultivars’ also. Please correct accordingly in the text wherever mentioned.

**Response:** After careful consideration, the authors thought ‘accessions’ would be appropriate. The 'varieties' has been replaced into ‘accessions’ in the revised manuscript.

3. How variable are the climatic conditions of the three research institutes?

**Response:** The climatic conditions of different growth sites of sea buckthorn samples has been added in S3 Table of the revised manuscript, with the caption ‘Geographical and climatic conditions at different sample collection sites of sea buckthorn in northern China’.

4. Line 109: ‘………provided 76 varieties’. Does this mean that all the 76 were grown at all the 3 fields? There is no clarity on this aspect in the M&M. Most quantitative traits exhibit a huge variation across environments. To study the phenotypic variations it would have been much informative if all the 76 varieties were grown together across all the three fields. Why was that not considered?

**Response:** Among the 76 accessions of sea buckthorn samples, 12 were grown in the Institute of Selection and Breeding of *Hippophae*, 52 were grown in the Research Institute of Berry and 12 were grown in the Jiuchenggong Breeding Base of Sea Buckthorn. These accessions are able to adapt to local climate and screened to be excellent germplasm.

The authors agreed the opinion that most quantitative traits exhibit a huge variation across environments. We did the comparative analysis on fruit morphological traits of the same cultivars grown in different cultivated fields in our early studies and the data was complemented in the ‘Results’ (S5 Table) of the revised manuscript. The aim in this study is to further screen the elite accessions from the 78 accession with good adaption to the environments of cultivated fields and prepare for the next step of MAB. In the follow-up study, the continuous observation of the environmental factors would be considered.

5. There is no mention of how these varieties were grown in the field, and data from how many plants were considered for the morphological and oil analysis. For eg. for hundred berry weight (HBW), berries were collected from how many different plants?

**Response:** The information has been supplemented in the introduction of the revised manuscript. The sea buckthorn samples in this study are collected from 235 individuals (2−5 ramet plants each accession) in 5 growth sites. The berries of each accession were pooled and frozen as quickly as possible at −20 °C. When all plant materials were harvested, the berries were transferred to –50 °C for storage until analysis. The related information has been supplemented in the M&M and Table 1.

6. Line 137: For the oil extraction and FA analysis, the authors mention that ‘each sample was analyzed three times’. Why weren’t three biological replicates taken for this analysis?

**Response:** The authors are sorry for the incorrect expression. In this study**,** three biological replicates were taken for every analysis. The sentence is corrected in the revised manuscript.

7. Line 180-181: The authors have used 17 previously developed SSR markers and 3 newly developed SSR markers using RNA-Seq. What was the basis of selection of just 3 new markers from the RNA-Seq. Why weren’t more markers deployed for the genetic characterization?

**Response:** The authors obtained many SSR sequences using RNA-Seq method and designed the primers to screen those SSR loci with polymorphism in sea buckthorn cultivars. We reported 17 developed SSR markers at first. In subsequent experiments, we screened 3 new SSR markers which also showed polymorphic amplification in sea buckthorn germplasm. RNA-Seq SSR loci with polymorphism in sea buckthorn germplasm were difficult to develop for that SSR markers derived from expressed region of genome showed high conservation to some extent in our study. That’s why no more markers deployed for the genetic characterization in sea buckthorn at now.

8. Line 180: Please reframe the sentence. It appears that the authors have done RNA-seq to generate the 3 new SSR markers. Although, the RNA-Seq had been done in previous study from where the 17 SSR were also developed (Reference 17).

**Response:** The authors accepted the advice and the sentence has been revised into ‘The Twenty polymorphic microsatellite loci (SSR) developed using RNA-Seq were evaluated and loci SB1-SB17 were previously published [17]’ in the revised manuscript.

9. Instead of ‘different origins’ that has been used repeatedly by authors throughout the text and tables, I suggest use the two different species and hybrid accessions.

**Response:** The authors accepted the good advice. Some ‘different origins’ were replaced to the ‘two different subspecies and hybrid accessions’ and the others were deleted in the revised manuscript.

10. Line 340: ‘All the primers’. Reframe this line. All primers did not give 59 bands. A total of 59 bands were amplified.

**Response:** The sentence has been revised according to the advice in the revised manuscript.

‘A total of 69 bands were amplified using the 23 primer pairs, of which 59 were polymorphic, accounting for 85.51% of all.’

11. Line 341: ‘accounting for 86.44%’ . Incomplete sentence, 86.44% of what??

**Response:** The sentence has been revised according to the advice in the revised manuscript.

12. Line 372: the 3 subgroups have been referred incorrectly. They are IIa, IIb and IIc.

**Response:** The names of 3 subgroups were corrected in the revised manuscript.

13. Line 421: ‘in comparison of populations’. Statement not clear. Please reframe.

**Response:** The phrase has been replaced to ‘in population identification’ in the revised manuscript.

14. Line 436: ‘gene sequences’. Are all the SSR markers used genic in nature?

**Response:** SSR can be divided into genomic SSRs and genic SSRs because of the resource of sequences used for SSR identification. Genic SSRs derived from transcriptome or expressed sequence tag sequences are located in expressed genes. These markers can be linked with important phenotypic characteristics through quantitative trait loci analysis. In this study, all SSR markers are genic SSRs.

15. Table 1: Could just be described as the ‘Accessions of sea buckthorn used for the study’

**Response:** The authors accepted the good advice and the title of Table 1 was revised into the ‘Accessions of sea buckthorn used for the study’.
