# Supplementary material for: Diversity in sea buckthorn (Hippophae rhamnoides L.) accessions with different origins based on morphological characteristics, oil traits, and microsatellite markers
Source: PLoS One. 2020 Mar 13;15(3):e0230356. doi: 10.1371/journal.pone.0230356 (PMC7069629; doi:10.1371/journal.pone.0230356)
Supplement: Attachment — Submitted filename: Comments.docx [file pone.0230356.s014.docx]

General Comments:

The manuscript has a lot of issues with the language. Many such sentences have been highlighted in the document attached. Some of these appear as two half statements fused. At other places, the sentences lack clarity. The authors need to re-frame all such statements.

The Materials & Methods needs to be revised at places (please see suggestions). Most trait measures in various tables lack the unit of measurements. Please incorporate that.

**Specific comments:**

1. The text contradictorily mentions the deployment of 20 SSR primer pairs at certain places (line 357, 450, Header of Table 5) and at other places (Line 27, 41, 46, 197, 358, 373, 383, 451, 482) the use of 23 SSR primer pairs has been mentioned. The supplementary table (S4) gives sequence information for 23, while its header says 20 SSR primers. Table 5, gives information for 23 markers although the Header says 20. Please ensure that all these ambiguities are taken care of.
2. Abstract says 69 polymorphic bands, while in results 59 polymorphic bands are mentioned. This ambiguity also needs to be addressed.

**Introduction:**

1. Line 62: ‘*Two important parameters in……..oil quantity are oil content*’. Oil content cannot be a parameter of oil quality. So, this statement needs modification.

2. Line 78: ‘*Due to small berries………………….artificial hybridization for elite accessions*.’ The statement needs to be reframed.

3. Line 98: ‘*The diversity analysis…………………………germplasm’*. The authors appear to have have fused to incomplete sentences. This needs to be re-wrtitten.

All other such statements have been highlighted in the document attached.

**Materials & Methods**

1. Line 139: ‘*There were three biological replicates………measurement*’.

Do the authors mean that 300 berries were taken for the analysis? 100 berries from 2-5 plants/ accession is a good enough number for the analysis.

2. Line142: ‘*…with over 20 measurements…for each*’

This is not clear. Do the authors mean 20 berries per accession?? And how many plants did these berries belong to?

3. For the oil extraction, were the seeds and fruit pulp weighed prior to oil extraction to maintain some uniformity. This has not been mentioned in the M&M.

The oil contents in both seeds and fruit pulp as mentioned in Line 153 is expressed as percentage. Percentage of what? Seed/pulp weight? The authors need to clearly mention that in the M&M.

In the results (Line 278), the authors mention *‘….highest oil content (24.68%) based on dry weight*.’ This means that the weight of the pulp/seed was considered. But, this has not been clearly mentioned either in the M&M or in the Table 3. The units for oil characteristic (min and max) have not been mentioned in the table.

4. Line 197: *‘Twenty-three polymorphic microsatellite loci (SSR) developed using RNA-Seq was evaluated and loci SB1-SB17 were previously reported’*.

Please mention here the names of the SSR markers (SB1-SB23). Nowhere in the text have they been mentioned except for tables. Then it can be mentioned that SB1-17 were previously deployed (Ref. 14).

The authors need to clearly mention in the introduction itself that in a previous study, RNA seq analysis was done to generate SSR markers and these were tested on 31 accessions. The 17 SSR markers developed in that study have been utilized in the present endeavor for genetic diversity assessment of larger set of accessions. **This description in the ‘introduction’ will bring more clarity in the text. This previous study and its outcomes should be mentioned clearly in the ‘Introduction’ so that its extension in the present study can be deciphered.**

**Results:**

1. **Line 246:** ‘*In previous mutilocation trials in Suiling (47°14′N, 127°06′E; 202 m) and Dengkou*

*(40°43′N, 106°30′E; 1053m, Inner Mongolia), the fruit characteristics of 11 large……*’.

How many berries per accession were taken for this analysis? The data should be represented as ± SD in Table S7.

2. **Line 302:** ‘*Small variations were found in the proportion of linoleic acid in seed oil (40.44 – 42.87%). Its proportion in hybrids were slightly higher than in ssp. mongolica (42.87% vs 42.10%*.....’

Are these differences significant?

3. Table 4: How is the oil content being measured? Total oil per gram weight of seeds and pulp or some other measure?

**Tables and Figures**

**1.** **Table 1**: Since the authors have already mentioned that 2-5 ramet plants were collected per accession. The columns indicating the number of plants taken per accession can be removed from the Table.

**2.** **Table 3**: The units for the min. and max values of the oil characteristics have not been mentioned in the table. Similarly mention the units of measurement for each of the component in Table 4.

**3.** **Table 3 & 4:** The different fatty acid names should be included in the first column. Example: Oleic (18:1), Palmitic acid (16:0) etc.

**4.** **Table S1:** This table again classifies all the lines used as ‘cultivars’. Are these accessions or cultivars? Please check.

**5.** **Table S3** carries a different header than the one that has been listed at the end of the manuscript. Please change that.

6. **Table S7**: The header for this table has been titled as Table S5. Please correct. Also it mentions ‘two experimental fields’ although it has data from three places. So, please correct.
