# Supplementary material for: Diversity in sea buckthorn (Hippophae rhamnoides L.) accessions with different origins based on morphological characteristics, oil traits, and microsatellite markers
Source: PLoS One. 2020 Mar 13;15(3):e0230356. doi: 10.1371/journal.pone.0230356 (PMC7069629; doi:10.1371/journal.pone.0230356)
Supplement: Attachment — Submitted filename: Response to Reviewers.docx [file pone.0230356.s015.docx]

General Comments:

The manuscript has a lot of issues with the language. Many such sentences have been highlighted in the document attached. Some of these appear as two half statements fused. At other places, the sentences lack clarity. The authors need to re-frame all such statements.

**Response:** Thank you for the valuable suggestion. The sentences mentioned above are all re-framed with clarity. And the revised manuscript was professionally edited by American Journal Experts (AJE, ID: HS1GCXH7) for the improvement in English quality.

The Materials & Methods needs to be revised at places (please see suggestions). Most trait measures in various tables lack the unit of measurements. Please incorporate that.

**Response:** Thank you for the valuable suggestion. The places mentioned above in the Materials & Methods have been revised according to the reviewers’ suggestions. And the unit of measurements has been added in the tables of the revised manuscript.

**Specific comments:**

1. The text contradictorily mentions the deployment of 20 SSR primer pairs at certain places (line 357, 450, Header of Table 5) and at other places (Line 27, 41, 46, 197, 358, 373, 383, 451, 482) the use of 23 SSR primer pairs has been mentioned. The supplementary table (S4) gives sequence information for 23, while its header says 20 SSR primers. Table 5, gives information for 23 markers although the Header says 20. Please ensure that all these ambiguities are taken care of.

**Response:** Twenty-three SSR primer pairs were used in this study. The number ‘20’ at certain places (line 357, 450, Headers of Table 5 and S4 Table) have been replaced by 23 in the revised manuscript.

2. Abstract says 69 polymorphic bands, while in results 59 polymorphic bands are mentioned. This ambiguity also needs to be addressed.

**Response:** The number of polymorphic bands is 59. It has been revised in the abstract of the revised manuscript.

**Introduction:**

1. Line 62: ‘*Two important parameters in……..oil quantity are oil content*’. Oil content cannot be a parameter of oil quality. So, this statement needs modification.

**Response:** The authors agreed with this opinion. The oil content is a parameter of oil yield. The sentence has been revised as bellow.

‘Two important parameters in analyzing oil yield and quality are oil content and fatty acid (FA) composition (referred to here as ‘oil traits’ for simplicity).’

2. Line 78: ‘*Due to small berries………………….artificial hybridization for elite accessions*.’ The statement needs to be reframed.

**Response:** Thank you for the valuable suggestion. The statement is reframed as bellow.

‘Due to the small berries and thorns of native cultivars (ssp. *sinensis*), which result in little economic value, the breeding of sea buckthorn has undergone different stages of development in China, such as introduction, domestication, seedling selection and artificial hybridization for elite accessions.’

3. Line 98: ‘*The diversity analysis…………………………germplasm’*. The authors appear to have fused to incomplete sentences. This needs to be re-written.

All other such statements have been highlighted in the document attached.

**Response:** Thank you for the valuable suggestion. All such statements are re-written in the revised manuscript.

**Materials & Methods**

1. Line 139: ‘*There were three biological replicates………measurement*’.

Do the authors mean that 300 berries were taken for the analysis? 100 berries from 2-5 plants/ accession is a good enough number for the analysis.

**Response:** Yes. 300 berries were taken for the analysis. We collected more than 300 berries per accession and the analyses of other nutrients were performed in our research work, e.g. vitamin C, vitamin E and carotenoids.

2. Line142: ‘*…with over 20 measurements…for each*’

This is not clear. Do the authors mean 20 berries per accession?? And how many plants did these berries belong to?

**Response:** The authors agreed with this view. It means averaged 20 determinations were done for each character. These berries were selected from the berry samples randomly collected from 2-5 ramet plants per accession. This sentence mentioned above has been re-framed for clarity in the revised manuscript as bellow.

**‘**The transverse and longitudinal diameters of berries (BTD and BLD) and the length, width and thickness of seeds (SL, SW and ST) were measured over 20 times each (on average) by micrometer calipers.’

3. For the oil extraction, were the seeds and fruit pulp weighed prior to oil extraction to maintain some uniformity. This has not been mentioned in the M&M.

The oil contents in both seeds and fruit pulp as mentioned in Line 153 is expressed as percentage. Percentage of what? Seed/pulp weight? The authors need to clearly mention that in the M&M.

In the results (Line 278), the authors mention *‘….highest oil content (24.68%) based on dry weight*.’ This means that the weight of the pulp/seed was considered. But, this has not been clearly mentioned either in the M&M or in the Table 3. The units for oil characteristic (min and max) have not been mentioned in the table.

**Reponse:** Thank you for the valuable suggestion. The method of lipid extraction was described by Yang and Kallio (2001). Samples (1 g) of seeds and fruit pulp were isolated from freeze-dried berries and lipids from the samples were extracted with chloroform/methanol (2:1, v/v) with mechanical homogenization of the tissues. The oil contents (percentages) in seeds and fruit pulp were calculated (oil % in seeds and lyophilized fruit pulp). The fatty acid composition was also expressed as a weight percentage of the total fatty acids. The units (weight percentages) for oil characteristics in Table 3 and Table 4 have been added in the revised manuscript.

4. Line 197: *‘Twenty-three polymorphic microsatellite loci (SSR) developed using RNA-Seq was evaluated and loci SB1-SB17 were previously reported’*.

Please mention here the names of the SSR markers (SB1-SB23). Nowhere in the text have they been mentioned except for tables. Then it can be mentioned that SB1-17 were previously deployed (Ref. 14).

The authors need to clearly mention in the introduction itself that in a previous study, RNA seq analysis was done to generate SSR markers and these were tested on 31 accessions. The 17 SSR markers developed in that study have been utilized in the present endeavor for genetic diversity assessment of larger set of accessions. **This description in the ‘introduction’ will bring more clarity in the text. This previous study and its outcomes should be mentioned clearly in the ‘Introduction’ so that its extension in the present study can be deciphered.**

**Reponse:** Thank you for the valuable suggestion. The sentences mentioned above have been re-framed for clarity in the revised manuscript as bellow.

‘Twenty-three polymorphic microsatellite loci (SB1-SB23) developed using RNA-Seq were evaluated. Of these, 17 (SB1-SB17) had been deployed in a previous study by the group [14].’

And the authors added the statements of 17 RNA-Seq SSR markers developed in our previous study and mentioned these SSR markers have been utilized in the present endeavor for genetic diversity assessment of larger set of accessions in the revised manuscript.

‘In our previous study, 17 RNA-Seq SSR markers (SB1-SB17) were developed and validated on 31 accessions, which were utilized in the present study for genetic diversity assessment of larger set of accessions [14].’

**Results**

1. **Line 246:** ‘*In previous mutilocation trials in Suiling (47°14′N, 127°06′E; 202 m) and Dengkou (40°43′N, 106°30′E; 1053m, Inner Mongolia), the fruit characteristics of 11 large……*’.

How many berries per accession were taken for this analysis? The data should be represented as ± SD in Table S7.

**Reponse:** 300 berries of each cultivar were randomly sampled and divided into 3 groups (100 berries were divided into 1 group) to determine the hundred berry weight (HBW). 20 berries of each cultivar were randomly sampled to determine the transverse, longitudinal diameters of berries and berry shape indices (BTD, BLD and BSI). The data has been represented as ± SD in S7 Table in the revised manuscript.

2. **Line 302:** ‘*Small variations were found in the proportion of linoleic acid in seed oil (40.44 – 42.87%). Its proportion in hybrids were slightly higher than in ssp. mongolica (42.87% vs 42.10%*.....’

Are these differences significant?

**Reponse:** These differences are significant despite small variations. The content of seed oil in hybrids is lower than that in ssp. *mongolica*. However, the proportion of linoleic acid (an important polyunsaturated fatty acid) in seed oil is higher in hybrids than that in ssp. *mongolica*, which showed high oil quality of seed oil in hybrids.

3. Table 4: How is the oil content being measured? Total oil per gram weight of seeds and pulp or some other measure?

**Reponse:** The method of lipid extraction was described by Yang and Kallio (2001). Samples (1 g) of seeds and fruit pulp were isolated from freeze-dried berries and lipids from the samples were extracted with chloroform/methanol (2:1, v/v) with mechanical homogenization of the tissues. The oil contents (percentages) in seeds and fruit pulp were calculated (oil % in seeds and lyophilized fruit pulp).

**Tables and Figures**

**1.** **Table 1**: Since the authors have already mentioned that 2-5 ramet plants were collected per accession. The columns indicating the number of plants taken per accession can be removed from the Table.

**Reponse:** The columns indicating the number of plants taken per accession have been removed from Table 1 in the revised manuscript.

**2.** **Table 3**: The units for the min. and max values of the oil characteristics have not been mentioned in the table. Similarly mention the units of measurement for each of the component in Table 4.

**Reponse:** The units of the oil characteristics have been added in the headers of Table 3 and Table 4.

**3.** **Table 3 & 4:** The different fatty acid names should be included in the first column. Example: Oleic (18:1), Palmitic acid (16:0) etc.

**Reponse:** Thank you for the valuable suggestion. The different fatty acid names are included in the first column of Table 3 and Table 4 according to the examples.

**4.** **Table S1:** This table again classifies all the lines used as ‘cultivars’. Are these accessions or cultivars? Please check.

**Reponse:** The ‘accession’ has replaced the ‘cultivar’ in S1 Table of revised manuscript.

**5.** **Table S3** carries a different header than the one that has been listed at the end of the manuscript. Please change that.

**Reponse:** The header of S3 Table listed at the end of the manuscript has been changed in the revised manuscript.

6. **Table S7**: The header for this table has been titled as Table S5. Please correct. Also it mentions ‘two experimental fields’ although it has data from three places. So, please correct.

**Reponse:** The header for S7 Table has been corrected. The mutilocation trials were performed in two experimental fields (Suiling and Dengkou). Russia is the country of origin of those cultivars and the related data were provided by the units where they were introduced.
