# Supplementary material for: Diversity in sea buckthorn (Hippophae rhamnoides L.) accessions with different origins based on morphological characteristics, oil traits, and microsatellite markers
Source: PLoS One. 2020 Mar 13;15(3):e0230356. doi: 10.1371/journal.pone.0230356 (PMC7069629; doi:10.1371/journal.pone.0230356)
Supplement: Attachment — Submitted filename: Response to Reviewers.docx [file pone.0230356.s017.docx]

Response to Reviewer' Comments letter to PLOS ONE

The authors thank two reviewers for their careful reading, comments and suggestion. We revised our manuscript in the best way as we could. Revised portions are marked in red in the revised manuscript. For the individual comments see our reply below.

**Reviewer #1:** As most of my queries have been attended, the MS may accepted for publication provided the other reviewer has also reached a similar decision.

**Response:** The authors thank for your acceptance of the revised MS.

**Reviewer #2:** The authors have made the suggested changes and the manuscript should be accepted for publication after few minor changes as suggested below. These have also been highlighted in the manuscript attached.

1. Some text changes are suggested: Line 36, 77, 78, 84, 87, 362, 375, 452, 461, 462, 489.

**Response:** The authors thank for your suggestions. The text changes mentioned above have been corrected in the revised MS. The details are as follows.

Line 36: The word ‘approximately’ has been deleted.

Line 77,78: The sentence has been revised to ‘However, the fruits of native cultivars are small and thorny and of low economic value, which encourages the breeding of sea buckthorn has undergone different stages of development in China’.

Line 84: The word ‘a’ has been deleted.

Line 87: The word ‘associated’ has been deleted.

Line 362: The word ‘in’ has been revised to ‘among’.

Line 375: The sentence has been changed into ‘The characteristics of 23 polymorphic SSR markers in sea buckthorn accessions are shown in Table 5’.

Line 452: The word ‘identify’ has been revised to ‘investigate the genetic relationships among 78 sea buckthorn accessions’.

Line 461: The phrase ‘between ssp. *sinensis* and ssp. *mongolica* accessions’ has been changed into ‘between accessions of ssp. *sinensis* and ssp. *mongolica*’.

Line 462: The words ‘This result illustrated’ has been revised to ‘This uniformity indicated’.

Line 489: The word ‘*Hippophae*’ has been revised to ‘*H.*’.

2. Line 307, 345: Lacks clarity.

**Response:** The authors accepted the opinion and revised these sentences for clarity. The details are as follows.

Line 307: The sentence ‘and had the highest value of the samples from the two different subspecies and hybrid accessions’ has been revised to ‘and showed the highest mean value among the two different subspecies and hybrid accessions’.

Line 345: The sentence ‘that were primarily provided by a marker of BLD’ has been revised to ‘that were primarily provided by the phenotypic character of BLD’.

3. Suggest changing Table 5 title to: ‘Genetic diversity analyses of 78 accessions of sea buckthorn germplasm using 23 SSR markers’.

**Response:** The authors thank for your suggestion. The title of Table 5 has been changed into ‘Genetic diversity analyses of 78 accessions of sea buckthorn germplasm using 23 SSR markers’ in the revised MS.

4. As previously suggested to the authors, since for each of the traits multiple measurements were taken, please include +/- SD values for each trait/accession in Table S6.

**Response:** The authors thank for you suggestion. The SD values for each trait/accession have been added in Table S6.
